# A dual‐function RNA balances carbon uptake and central metabolism in *Vibrio cholerae*

**DOI:** 10.15252/embj.2021108542

**Published:** 2021-10-06

**Authors:** Kavyaa Venkat, Mona Hoyos, James RJ Haycocks, Liam Cassidy, Beatrice Engelmann, Ulrike Rolle‐Kampczyk, Martin von Bergen, Andreas Tholey, David C Grainger, Kai Papenfort

**Affiliations:** ^1^ Institute of Microbiology Friedrich Schiller University Jena Germany; ^2^ Institute of Microbiology and Infection University of Birmingham Birmingham UK; ^3^ Systematic Proteome Research & Bioanalytics University of Kiel Kiel Germany; ^4^ Helmholtz Centre for Environmental Research‐UFZ Leipzig Germany; ^5^ Microverse Cluster Friedrich Schiller University Jena Jena Germany

**Keywords:** citrate synthase, dual‐function RNA, Hfq, small protein, *Vibrio cholerae*, Microbiology, Virology & Host Pathogen Interaction, RNA Biology

## Abstract

Bacterial small RNAs (sRNAs) are well known to modulate gene expression by base pairing with *trans*‐encoded transcripts and are typically non‐coding. However, several sRNAs have been reported to also contain an open reading frame and thus are considered dual‐function RNAs. In this study, we discovered a dual‐function RNA from *Vibrio cholerae*, called VcdRP, harboring a 29 amino acid small protein (VcdP), as well as a base‐pairing sequence. Using a forward genetic screen, we identified VcdRP as a repressor of cholera toxin production and link this phenotype to the inhibition of carbon transport by the base‐pairing segment of the regulator. By contrast, we demonstrate that the VcdP small protein acts downstream of carbon transport by binding to citrate synthase (GltA), the first enzyme of the citric acid cycle. Interaction of VcdP with GltA results in increased enzyme activity and together VcdR and VcdP reroute carbon metabolism. We further show that transcription of *vcdRP* is repressed by CRP allowing us to provide a model in which VcdRP employs two different molecular mechanisms to synchronize central metabolism in *V. cholerae*.

## Graphical abstract

A screen for repressors of cholera toxin production identifies VcdPR as the first *V. cholerae* RNA that acts both as a non‐coding expression modulator and by encoding a small protein.

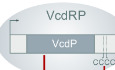

## Introduction

To make the most of their environment, bacterial control of nutrient uptake and utilization is precisely regulated. In addition, pathogenic microorganisms frequently couple the production of their virulence factors with nutrient availability and the overall metabolic status of the cell (Eisenreich *et al*, 2010). The control of metabolic functions has traditionally been attributed to the activities of regulatory proteins that switch on and off the transcription of relevant genes. However, this view has been challenged in the past few years as ever more RNA‐based regulators have been found to modulate microbial metabolism and, more specifically, carbon uptake and utilization (Bobrovskyy & Vanderpool, 2013; Papenfort & Vogel, 2014).

The largest class of bacterial RNA regulators is called small RNAs (sRNAs) (Waters & Storz, 2009). The sRNAs are typically 50–300 nucleotides (nt) in length and expressed from discrete genes, or are clipped off from the 3′ untranslated (UTR) region of mRNAs (Miyakoshi *et al*, 2015). The majority of sRNAs act by base pairing with either *cis*‐ or *trans*‐encoded mRNAs and frequently require the aid of RNA chaperone proteins, *e.g*., Hfq or ProQ, to accomplish RNA duplex formation and thus gene regulation (Ng Kwan Lim *et al*, 2021). A typical bacterium might encode ˜100–200 different sRNAs (Hör *et al*, 2020) regulating a wide range of microbial processes including virulence (Barquist *et al*, 2016), biofilm formation (Svenningsen, 2018), intercellular communication (Papenfort & Bassler, 2016), stress response (Fröhlich & Gottesman, 2018), iron acquisition (Salvail & Masse, 2012), and central metabolism (Bobrovskyy & Vanderpool, 2013), to name just a few.

A unique group of regulators among the larger class of sRNAs are the so‐called dual‐function RNAs. Dual‐function RNAs are chimeric transcripts that serve as both: base‐pairing riboregulators and mRNAs. Today, several dual RNAs with confirmed base‐pairing and protein‐coding functions have been reported. These include dual RNAs from Gram‐positive bacteria such as Pel from *Streptococcus pyogenes* (Mangold *et al*, 2004), SR1 from *Bacillus subtilis* (Gimpel & Brantl, 2016), Psm‐mec and RNAIII from *Staphylococcus aureus* (Kaito *et al*, 2011; Bronesky *et al*, 2016), as well as SgrS from Gram‐negative species (Wadler & Vanderpool, 2007). In the majority of cases, a regulatory function for the RNA element was detected first and only detailed analysis of the mechanistic principles underlying these regulators exposed the presence of a small protein‐coding open reading frame (ORF; Raina *et al*, 2018). Of note, several additional potential dual RNAs have been reported, *e.g*., PhrS from *Pseudomonas aeruginosa* (Sonnleitner *et al*, 2011), VR‐RNA from *Clostridium perfringens* (Shimizu *et al*, 2002), RivX from *S. pyogenes* (Roberts & Scott, 2007), and Scr5239 from *Streptomyces coelicolor* (Vockenhuber *et al*, 2015); however, the functions of their respective small proteins are currently unknown. In fact, genome‐wide screens aiming to discover dual‐function RNAs have not yet been performed. Hence, our knowledge of how these regulators work at the molecular level, and the biological processes they are involved in are still very limited.

In this work, we discovered a dual‐function RNA, VcdRP (*Vibrio cholerae* dual RNA and protein), from *V. cholerae*, the causative agent of cholera disease. *V. cholerae* colonizes and infects the intestines of humans, and production of the cholera toxin (CTX) is key to this process (Rivera‐Chavez & Mekalanos, 2019). Much like other enteric pathogens, *V. cholerae* relies on the availability of certain carbohydrates to express its virulence genes, a large part of which is integrated through the activity of the dual transcriptional regulator, CRP (Liang *et al*, 2007; Manneh‐Roussel *et al*, 2018). Activity of CRP is controlled by cAMP (cyclic‐adenosine‐monophosphate) and we show that both CRP and cAMP are required to inhibit *vcdRP* transcription. We further discovered that VcdRP over‐expression inhibits CTX production, which we attribute to the repression of mRNAs encoding the sugar transport proteins by VcdR. Specifically, our data demonstrate that VcdR employs a sequence of four consecutive cytosines located in the 3’ end of the regulator to base pair with the translation initiation sites of *ptsG*, *treB*, and *nagE*, producing the glucose‐, trehalose‐, and N‐acetylglucosamine‐specific components of the phosphotransferase carbohydrate transporter (PTS), respectively (Tchieu *et al*, 2001). In addition, VcdR also represses the *ptsHI* mRNA encoding two sugar non‐specific phosphocarrier proteins required for PTS‐dependent carbohydrate uptake (Gorke & Stulke, 2008). In contrast, the VcdP small protein does not interfere with carbohydrate transport. Instead, the small protein binds to and increases the activity of GltA (citrate synthase), which is the first enzyme of the citric acid cycle catalyzing the condensation of oxaloacetate and acetyl coenzyme A to produce citrate and coenzyme A. Mutation or over‐expression of VcdR and VcdP modulate central metabolism indicating that this dual‐function RNA acts to balance the metabolic flux between glycolysis and the citric acid cycle and thus optimize carbohydrate utilization in *V. cholerae*. Our study identifies the first dual‐function RNA in *V. cholerae* and highlights the intricate interplay of RNA‐based regulation and small protein function in these regulators.

## Results

### VcdRP inhibits CTX production in *V. cholerae*

**Figure 1:**
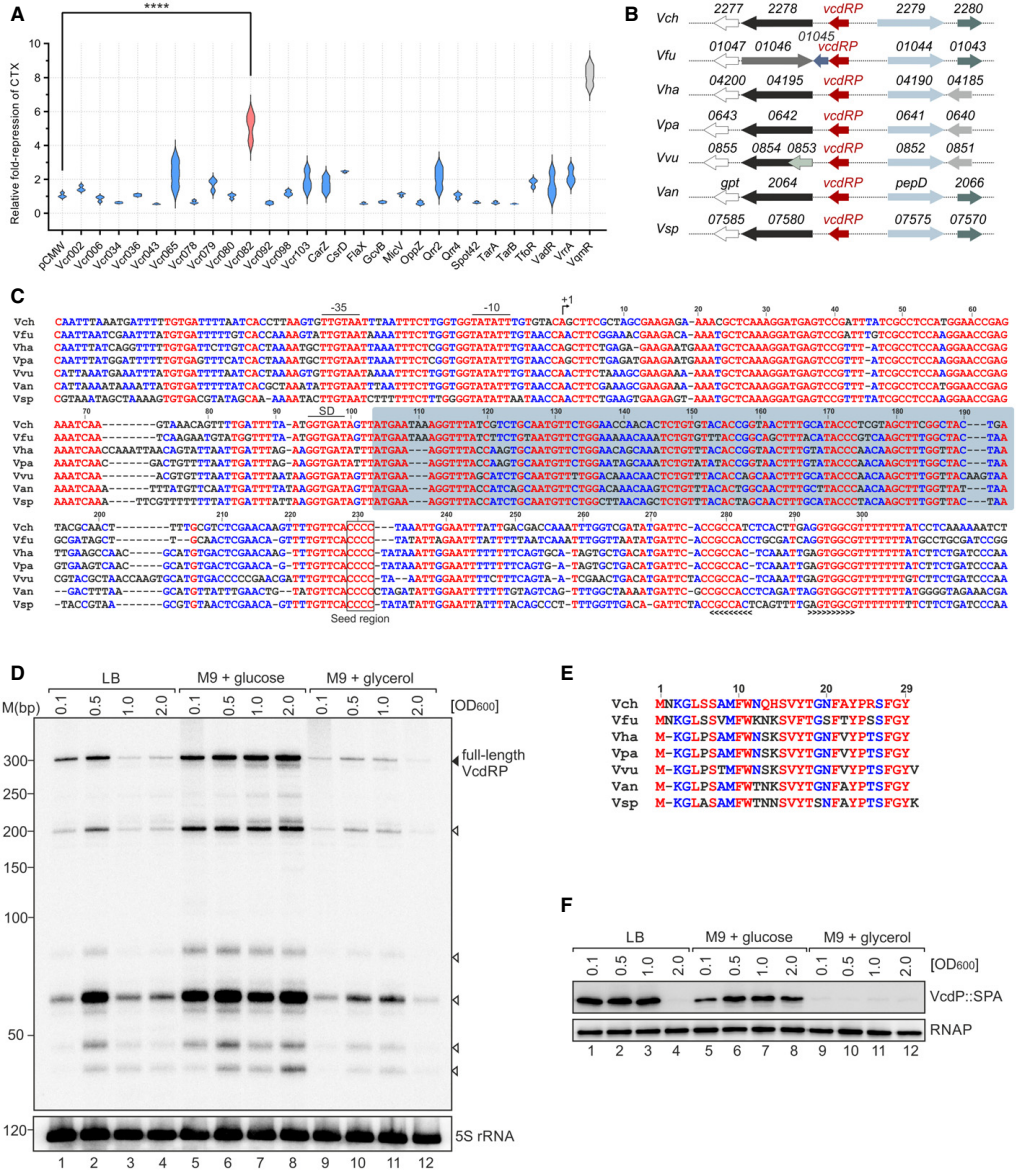
The VcdRP dual‐function RNA regulator inhibits CTX production AViolin plot of *V. cholerae* Δ*hapR* cells expressing the indicated sRNAs (*x‐axis*) plotted as relative fold repression (*y‐axis*) of secreted CTX protein fractions in comparison with a control strain (pCMW) grown under AKI conditions. Indicated in gray is the only known repressor of CTX to date (VqmR) and indicated in pink is the yet uncharacterized sRNA, Vcr082 (VcdRP).BGene synteny analysis of the genomic loci encoding *vcdRP* (formerly known as *vcr082*) in various *Vibrio* strains. Homologous genes are depicted using the same colors.CAlignment of *vcdRP* sequences, including the promoter regions from various *Vibrio* species: *V*. *cholerae* (Vch), *V*. *furnissi* (Vfu), *V. vulnificus* (Vvu), *V. harveyi* (Vha), *V. parahaemolyticus* (Vpa), *V*. *anguillarum* (Van), and *V. splendidus* (Vsp). The −35 box, −10 box, transcriptional start site (TSS; arrow), the start and stop positions of VcdP (blue box), the highly conserved base‐pairing region (four consecutive cytosines), and rho‐independent terminator (brackets) are indicated.DNorthern blot analysis of *V. cholerae* wild‐type strain examined for the expression of VcdRP monitored over bacterial growth in the indicated media. The solid triangle represents the band corresponding to the full‐length primary *vcdRP* transcript, whereas the open triangles correspond to the different processed isoforms. Probing with 5S rRNA confirmed equal loading.ESequence conservation of the 29 amino acid small protein, VcdP, in different *Vibrio* species.FWestern blot analysis of the production of chromosomally encoded VcdP::SPA, corresponding to the same growth conditions as the Northern blot in (D). The SPA epitope contains the 3×FLAG and the calmodulin binding peptide sequences separated by a TEV protease cleavage site (Zeghouf *et al*, 2004). Therefore, anti‐Flag antibody was used to examine the levels of VcdP. RNAP served as a loading control. Data information: In (A), *n* = 3 independent biological replicates. Statistical significance was determined using one‐way ANOVA and post hoc Dunnett’s test. **** corresponds to *P*‐value ≤ 0.0001. Source data are available online for this figure.

Spatio‐temporal control of CTX production is critical for virulence of *V. cholerae* and there are various transcriptional and post‐transcriptional regulators that govern CTX levels (Rivera‐Chavez & Mekalanos, 2019; Pant *et al*, 2020). For example, we have recently shown that the quorum sensing‐controlled VqmR sRNA inhibits *ctx* expression by blocking the translation of the *aphA* mRNA, encoding the initial activator of virulence gene production in *V. cholerae* (Herzog *et al*, 2019). We were thus interested to identify additional sRNAs affecting CTX production and developed a simple forward genetic screen in which we used Western blotting to monitor CTX levels in response to plasmid‐borne expression of 28 different sRNAs in *V. cholerae* (Papenfort *et al*, 2015; Huber *et al*, 2020). As expected, VqmR strongly reduced CTX production (˜8‐fold) when compared to a parental strain carrying a control plasmid (Fig 1A). Similarly, over‐expression of the yet uncharacterized Vcr082 sRNA, from hereon called VcdRP, led to a significant reduction of CTX (˜5‐fold; Fig 1A), which prompted us to further investigate this regulator.

### VcdRP is a dual RNA regulator

**Figure EV1:**
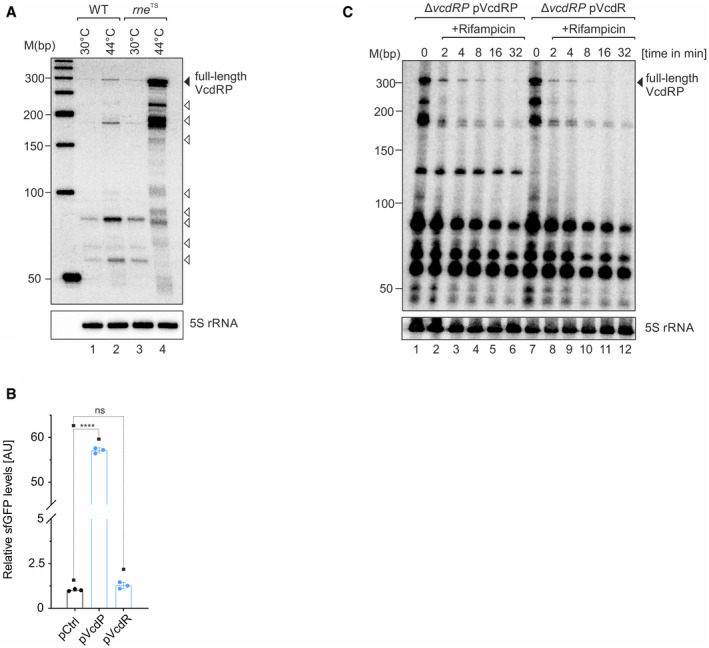
Effect of VcdR/P variants on translation and RNA stability (related to Fig 1) ARNase E‐mediated processing of VcdRP. *V. cholerae* wild‐type and *rne* ^TS^ strains were grown at 30°C to stationary phase (OD_600_ of 2.0). Cultures were divided into half and were allowed to continue growing at either 30 or 44°C for 30 min. The cleavage patterns of VcdRP was monitored on a Northern blot. The solid triangle refers to the band corresponding to the full‐length primary *vcdRP* transcript, whereas the open triangles correspond to the different processed isoforms. Probing with 5S rRNA confirmed equal loading.BRelative fluorescence intensities (*y*‐axis) of *E. coli* strains harboring an empty control plasmid (pCtrl) or translational fusions of *vcdRP* expression plasmids fused to *sfGFP*, expressing either only the 29 amino acid small protein (pVcdP) or a STOP codon introduced in the 3^rd^ codon of the ORF (pVcdR, *x*‐axis). Cells were grown in LB to OD_600_ of 1.0 and fluorophore production was measured. The fluorescence of pCtrl was set to 1.CNorthern blot analysis of *V. cholerae* Δ*vcdRP* strains harboring either pVcdRP or pVcdR plasmids examined for the stability of *vcdRP*. The cultures were grown in LB medium transcription was inhibited by the addition of rifampicin (f.c. 250 μg/ml) at OD_600_ of 1.0. RNA samples were harvested prior to, as well as at 2, 4, 8, 16, and 32 min post‐rifampicin treatment. The solid triangle represents the full‐length VcdRP transcript. Probing for 5S rRNA served as loading control. Data information: Data in (B) are presented as mean ± SD, *n* = 3 independent biological replicates. Statistical significance was determined using one‐way ANOVA and post hoc Tukey’s multiple comparisons test. The *P*‐value is summarized as follows ‐ ns for *P* > 0.05 and **** for *P* ≤ 0.0001. Source data are available online for this figure.

The *vcdRP* gene is located between the *vc2278* and *vc2279* genes on the negative strand of the larger *V. cholerae* chromosome (Fig 1B). The sRNA is present in numerous other *Vibrios* and carries several highly conserved sequence elements along with a putative Rho‐independent terminator at its 3′ end (Fig 1C). Northern blot analysis of total RNA isolated from *V. cholerae* revealed that full‐length VcdRP accumulates as a ˜306 nt transcript (Fig 1D), which is further processed by RNase E into several shorter isoforms (Fig EV1A and Hoyos *et al*, 2020). In rich media, expression of VcdRP is highest at low cell densities (OD_600_ of 0.1 and 0.5) and decreases at higher cell densities (Fig 1D; lanes 1, 2 versus 3, 4). In contrast, when cultivated in minimal media supplemented with glucose, VcdRP levels remained high at all cell densities (lanes 5–8) and we observed the inverse effect when we swapped the carbon source to glycerol (lanes, 9–12). Together, these experiments suggested that expression of VcdRP is controlled by the availability of carbohydrates in the media.

We have previously determined the transcriptional start site of *vcdRP* (Papenfort *et al*, 2015) and demonstrated that the VcdRP transcript interacts with Hfq *in vivo* (Huber *et al*, 2020). Whereas typical Hfq‐binding sRNAs range in size between 50–250 nts (Hershberg *et al*, 2003), the > 300 nts observed for VcdRP (Fig 1C and D) encouraged us to search for additional functions encoded by this regulator. Indeed, closer investigation of the *vcdRP* alignment allowed us to predict the presence of a conserved 29 amino acid small protein initiating translation at position 104 of the *vcdRP* transcript and terminating at position 193 (Fig 1C and E). A translational fusion of this predicted ORF to *gfp* resulted in high levels of the fluorescent protein and introduction of a STOP codon at the 3^rd^ codon abrogated this effect (Fig EV1B). In addition, we added the SPA epitope (Zeghouf *et al*, 2004) to the C‐terminus of the chromosomal ORF and validated protein production by Western blot. In line with our previous Northern blot analysis, in rich media, protein levels were highest a low cell densities and expression was increased in the presence of glucose and decreased when glycerol served as the main carbon source (Fig 1F). Of note, the STOP codon mutation at the 3^rd^ codon of VcdP also reduced the stability of the full‐length VcdRP transcript, while it did not affect stability of the shorter VcdRP isoforms (Fig EV1C). Taken together, our data suggest VcdRP is the first dual‐function RNA identified in *V. cholerae*, in which VcdR refers to the regulatory RNA element, while VcdP stands for the small protein.

### CRP inhibits transcription of *vcdRP*

**Figure 2:**
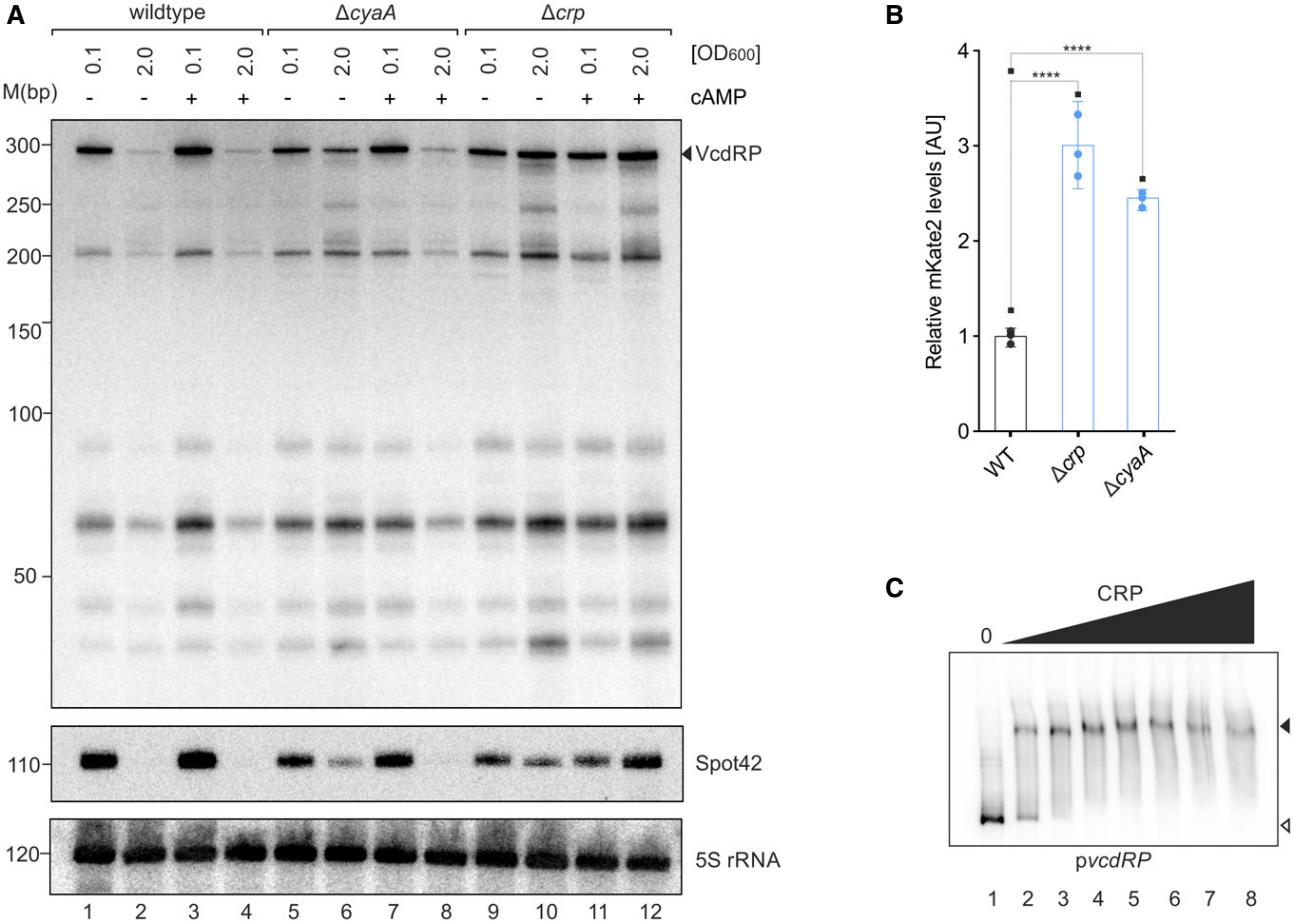
CRP‐mediated transcriptional control of *vcdRP* ANorthern blot analysis of the expression of VcdRP and Spot42 sRNA monitored at low cell density (OD_600_ of 0.1) and high cell density (OD_600_ of 2.0), in the absence (−) or presence (+) of cAMP (5 mM f.c.) supplemented externally in LB medium. The solid triangle indicates the band corresponding to the full‐length primary transcript. Probing with 5S rRNA confirmed equal loading.BRelative fluorescence intensities of *V. cholerae* wild‐type or mutants lacking *crp* or *cyaA* harboring a transcriptional reporter plasmid of *vcdRP* fused to *mKate2*. Cells were grown in LB to OD_600_ of 1.0 and fluorophore production was measured. The fluorescence of WT was set to 1.CElectrophoretic mobility shift assay (EMSA) showing that CRP protein binds to the *vcdRP* promoter sequence. Migration of the [^32^P] end‐labeled *vcdRP* promoter fragment in the absence and presence of increasing concentrations of purified CRP protein (indicated by the black triangle above the gel) was determined by native polyacrylamide gel electrophoresis and autoradiography. The open triangle indicates free DNA whereas the filled triangle indicates VcdRP in complex with CRP. Final concentrations of CRP used in each reaction from left to right (lanes 1–8) were as follows: 0, 0.35, 0.7, 1.4, 2.1, 2.8, 3.5, and 4.2 µM. Radiolabeled DNA and CRP were incubated at 37°C for 20 min in buffer containing 0.2 mM cAMP, before being loaded on a 7.5% non‐denaturing polyacrylamide gel. Data information: In (B), data are presented as mean ± SD, *n* = 3 independent biological replicates. Statistical significance was determined using one‐way ANOVA and post hoc Dunnett’s multiple comparisons test. The *P*‐value is summarized as follows ‐ **** for *P* ≤ 0.0001. Source data are available online for this figure.

Northern blot analysis revealed that carbohydrate utilization affects *vcdRP* production (Fig 1D). We thus speculated that *vcdRP* expression might be controlled by a transcriptional regulator involved in carbon metabolism, which prompted us to search for conserved sequence elements in the *vcdRP* promoter. Indeed, we were able to identify a sequence matching the reported binding site for the cyclic AMP (cAMP) receptor protein (CRP) in *V. cholerae* (Manneh‐Roussel *et al*, 2018). CRP is a dual transcriptional regulator, and DNA binding typically requires interaction of cAMP (produced by adenylate cyclase, CyaA) with CRP (Gorke & Stulke, 2008). To test the effect of CRP on *vcdRP* transcription, we used Northern blot analysis to compare VcdRP expression of *V. cholerae* wild‐type, Δ*cyaA*, and Δ*crp* strains cultivated in rich media to low (OD_600_ of 0.1) and high (OD_600_ of 2.0) cell densities. In contrast to wild‐type cells, expression of VcdRP in *cyaA*‐ or *crp*‐deficient cells was high under both growth conditions (Fig 2A, lane 2 versus lanes 6 and 10). For Δ*cyaA* cells, we were able to suppress elevated VcdRP levels at high cell densities by external addition of cAMP (lane 8), whereas cAMP had little or no effect on the expression of VcdRP in the *crp* mutant (lane 12). Of note, we obtained a highly similar expression pattern when we probed the same Northern blot for Spot 42 (Fig 2A), whose promoter has previously been documented to be repressed by CRP (Polayes *et al*, 1988). We confirmed these results using a plasmid‐borne transcriptional reporter of the *vcdRP* promoter fused to the *mKate2* fluorescent protein gene. In line with our expectation, mutation of either *crp* or *cyaA* resulted in increased mKate2 production (Fig 2B), whereas deletion of the CRP binding site in the *vcdRP* promoter abrogated this effect (Appendix Fig S1A and B). Finally, we performed EMSA (electrophoretic mobility shift assay) of the *vcdRP* promoter DNA with purified CRP, which demonstrated direct binding of CRP (Fig 2C). Taken together, our results indicate that CRP inhibits the expression of *vcdRP* by binding to a conserved sequence motif upstream of the −35 box in the promoter DNA.

### Transcriptomic analyses of VcdR and VcdP function

**Figure 3:**
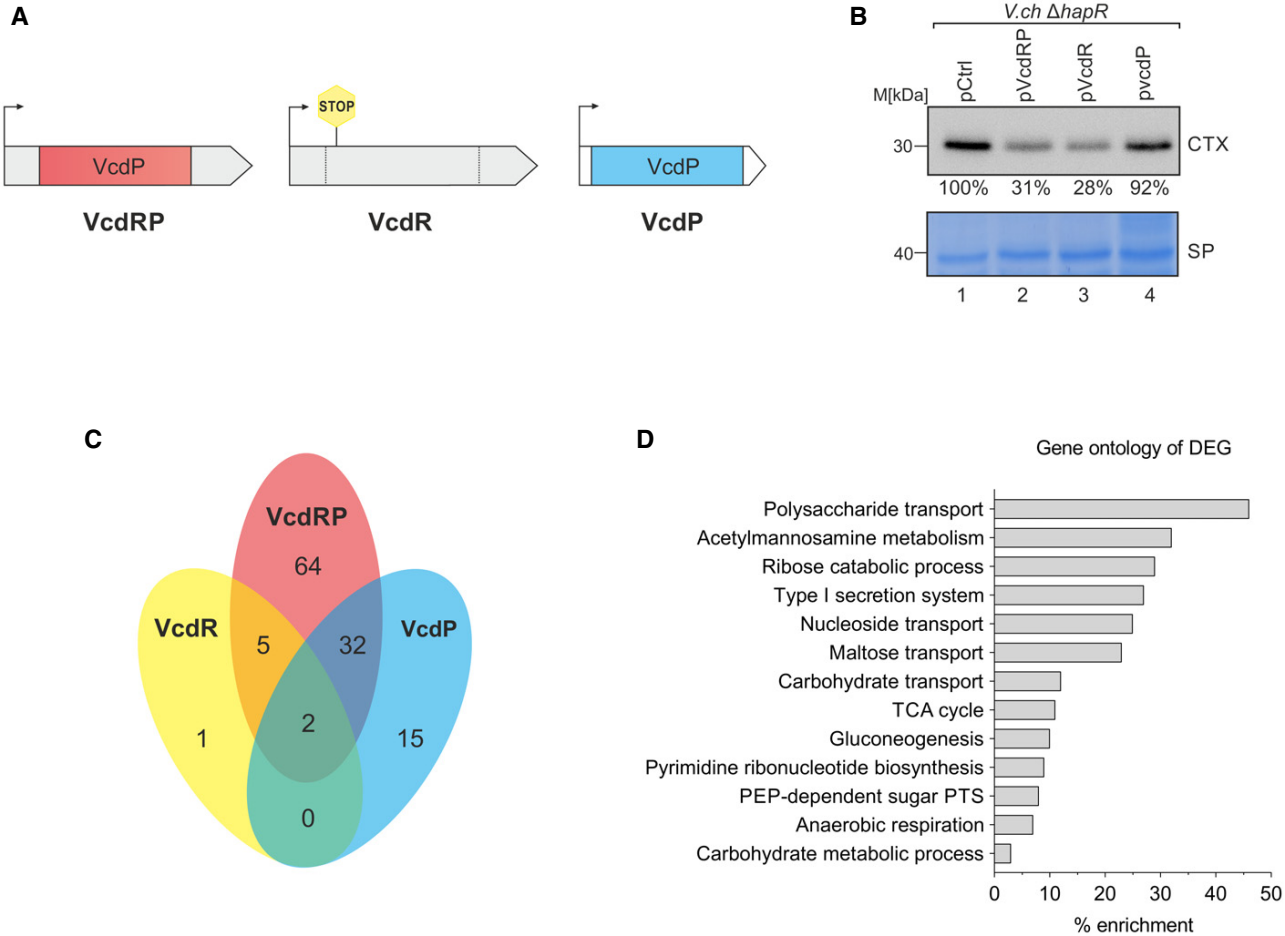
VcdRP regulates central metabolism genes AA schematic representation of the three VcdRP and VcdR/P variants used for the transcriptome analysis. Left to right: first, the native VcdRP variant which harbors both the small protein and the base‐pairing region. Next, a STOP codon was introduced in the 3^rd^ codon of *vcdP* ORF as a readout for VcdR devoid of VcdP. Finally, the sequence of the VcdP codons was scrambled such that the correct small protein is translated but the nucleotide sequence of the scrambled *vcdP* variant is significantly altered when compared to native *vcdRP*. This variant also carries an artificial 5′UTR and a terminator. The arrows indicate the TSS.BWestern blot analysis (top) of CTX levels detected in secreted protein fractions of *V. cholerae* Δ*hapR* cells grown under AKI conditions and carrying the indicated plasmids. Coomassie‐stained SDS gel (bottom) confirmed equal loading of the protein fractions. The normalized band intensities are indicated for each expression plasmid relative to pCtrl, which was set to 100%.CA Venn diagram of the differentially expressed genes (DEG) among VcdRP (red), VcdR (yellow), and VcdP (blue). RNA samples were harvested from *V. cholerae* Δ*vcdRP* strains carrying pBAD‐based plasmids depicted in (A), including an empty vector control. Genes with a total count cutoff > 10 in all samples, with an absolute fold‐change ≥ 2.0 and a FDR‐adjusted *P*‐value ≤ 0.05 were considered to be differentially expressed.DGene enrichment analysis of the DEG shown in (C) using gene ontology analysis. Data information: The differentially expressed genes in (C) are shown in Appendix Table S1 that is color‐coded. Source data are available online for this figure.

**Figure EV2:**
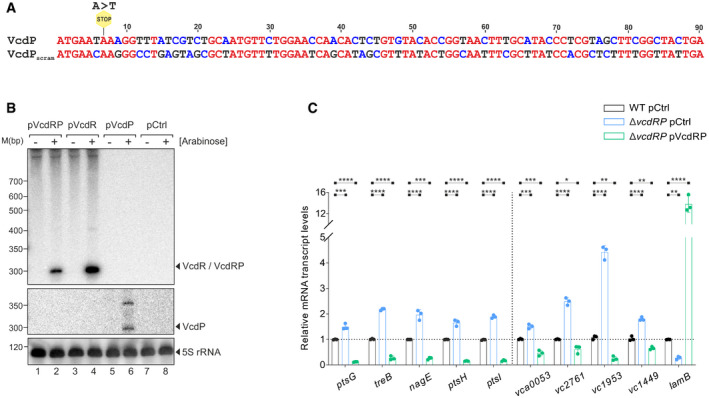
VcdR/P variants used for the transcriptome analysis (related to Fig 3) ATo disentangle the protein‐coding function from the base‐pairing activity of *vcdRP*, the 3^rd^ codon of the ORF was mutated from AAA to TAA (thus introducing a STOP codon, yellow). To generate the protein variant devoid of its RNA function, an over‐expression plasmid was created carrying only the codon‐modified *vcdP* ORF. The sequence of the VcdP codons were scrambled (VcdP_scram_) such that the correct protein is translated, but the sequence of the two VcdR base‐pairing regions is significantly changed.BNorthern blot analysis of *V. cholerae* Δ*vcdRP* strains carrying pBAD‐based plasmids (pVcdRP, pVcdR, and pVcdP) or an empty vector control (pCtrl). RNA samples were harvested before (−) and after (+) L‐arabinose induction for 15 min at OD_600_ of 0.1. The solid triangles indicate the expression of the different VcdR/P variants. Probing with 5S rRNA confirmed equal loading.CqRT‐PCR analyses of the indicated mRNA transcripts (x‐axis) measured on *V. cholerae* wild‐type and Δ*vcdRP* strains harboring an empty vector control (pCtrl) or Δ*vcdRP* carrying *vcdRP* expression plasmid (pVcdRP). The strains were grown in M9 medium supplemented with 0.4% glucose and 0.4% casaminoacids, and samples were collected at OD_600_ of 0.1. Relative fold changes were calculated with respect to wild‐type pCtrl set to 1. *recA* served as the reference housekeeping gene for all the measurements. Data information: Data in (C) are presented as mean ± SD, *n* = 3 independent biological replicates. Statistical significance was determined using one‐way ANOVA and post hoc Dunnett’s multiple comparisons test. The *P*‐values are summarized as follows: ns for *P* > 0.05, * for *P* ≤ 0.05, ** for *P* ≤ 0.01, *** for *P* ≤ 0.001, and **** for *P* ≤ 0.0001. Source data are available online for this figure.

Our previous results indicated that VcdRP could act as a dual‐function RNA (Fig 1), containing the VcdP small protein and a potential regulatory RNA element, VcdR. To study the role of VcdRP in *V. cholerae*, both in concert, as well as the distinct VcdP and VcdR elements, we compared the regulatory functions of *V. cholerae* carrying a control vector with those expressing VcdRP, VcdR, or VcdP from a multi‐copy plasmid. To express VcdR without VcdP, we introduced a STOP codon at the 3^rd^ position of the *vcdP* ORF (Figs 1C and 3A). For sole expression of VcdP, we maintained the protein sequences; however, we deleted the sequences flanking the *vcdP* ORF and scrambled its DNA sequence to avoid potential regulatory effects originating from the transcript (Figs 3A and EV2A). We discovered that VcdRP and VcdR both reduced CTX levels, whereas VcdP had no effect on the production of the protein (Fig 3B), suggesting that the RNA element in VcdRP is responsible for CTX inhibition.

To correlate these results with global transcriptomic changes occurring in response to VcdR/P, we next cloned all variants onto another plasmid under the control of the inducible pBAD promoter and confirmed expression by Northern blotting (Fig EV2B). RNA sequencing experiments obtained from these constructs revealed that pulse expression (15 min) of VcdRP resulted in differential regulation of 103 genes (84 upregulated/19 repressed). In contrast, VcdP modulated the expression of 49 genes (41 upregulated / 8 repressed), whereas VcdR led to a change in 8 genes (2 upregulated / 6 repressed) (Fig 3C and Appendix Table S1). As expected, the majority of genes regulated by either VcdP (34/49) or VcdR (7/8) were also differentially expressed in response to VcdRP. However, regulation of 64 genes was specific to VcdRP, suggesting that simultaneous expression of VcdR and VcdP could have a synergistic effect on the cell. Functional annotation of the genes differentially expressed in response to VcdRP showed a high proportion of genes with potential functions in metabolic processes and specifically carbon metabolism (Fig 3D), in line with the CRP‐mediated transcriptional control reported above.

To validate these results and to evaluate the regulatory effect of chromosomally produced VcdRP, we next compared the expression of 10 candidate genes (*ptsG*, *treB*, *nagE*, *ptsH*, *ptsI* for VcdR and *vca0053*, *vc2761*, *vc1953*, *vc1449*, *lamB* for VcdP) in wild‐type, Δ*vcdRP*, and VcdRP over‐expression cells (Fig EV2C). In all cases, we observed that genes differentially controlled by VcdR or VcdP in the RNA‐seq experiment (Appendix Table S1) showed the opposite regulatory trend in cells lacking *vcdRP*, whereas plasmid‐borne *vcdRP* complementation reversed this effect. In summary, we conclude that both VcdR and VcdP modulate the expression of several genes, which are frequently linked to carbon uptake and utilization by *V. cholerae*.

### Post‐transcriptional control of PTS‐coding mRNAs by VcdR

**Figure 4:**
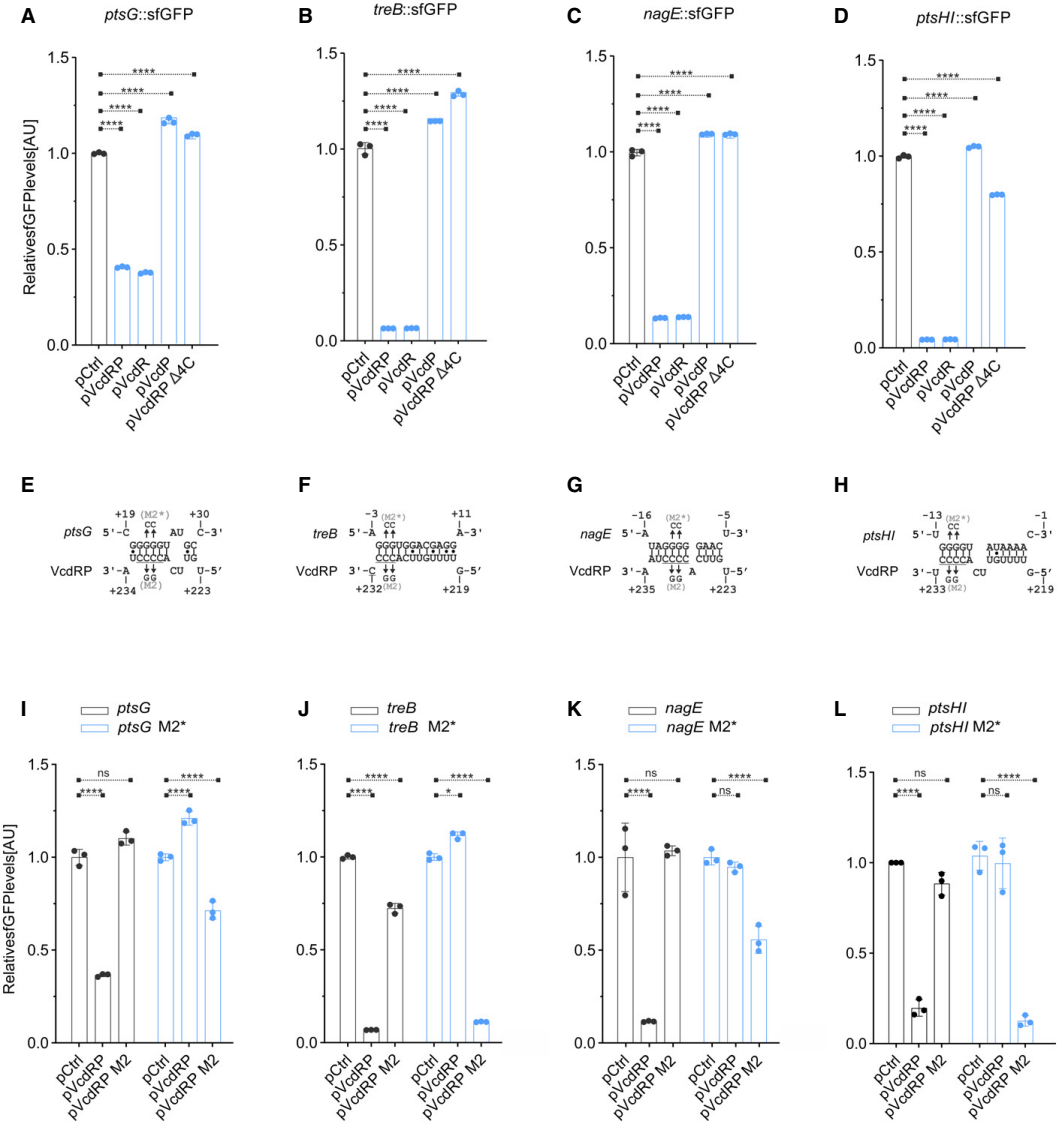
VcdR is a direct inhibitor of the synthesis of PTS sugar transport proteins A–DRelative fluorescence intensities (*y*‐axis) of *E. coli* strains harboring the gene‐specific translational reporters for *ptsG* (A), *treB* (B), *nagE* (C), and *ptsHI* (D), fused to *sfGFP*, combined with either an empty vector control (pCtrl) or *vcdRP* expression plasmids (pVcdRP, pVcdR, pVcdP, and pVcdRP Δ4C, *x*‐axis). Cells were grown in LB to OD_600_ of 1.0 and fluorophore production was measured. The fluorescence of pCtrl (for each reporter fusion) was set to 1.E–HPredicted base‐pairing sequences for *ptsG* (E), *treB* (F), *nagE* (G), *and ptsHI* (H) to VcdRP. The numbers indicate the distances relative to the TSS for VcdRP, and the start codons of the respective mRNA sequences. Underlined is the conserved stretch of four consecutive cytosines that corresponds to the seed region indicated in (1C). The M2* and M2 mutations refer to the site‐directed mutagenesis of two guanines/cytosines in positions 2 and 3 of the seed region to two cytosines/guanines in the corresponding mRNA/VcdRP, respectively.I–LRelative fluorescence intensities (*y*‐axis) of *E. coli* strains harboring the gene‐specific translational reporters as in (A–D) or the corresponding M2* variant for each gene, combined with an empty control plasmid or *vcdRP* expression plasmids (pVcdRP and pVcdRP M2) measured using plate reader (I–K) or by Western blot analysis (L). The fluorescence of pCtrl (for each reporter fusion) was set to 1. Data information: Data in (A–D) and (I–L) are presented as mean ± SD, *n* = 3 independent biological replicates. Statistical significance was determined using two‐way ANOVA and post hoc Tukey’s multiple comparisons test. The *P*‐value is summarized as follows—ns for *P* > 0.05, * for *P* ≤ 0.05, and **** for *P* ≤ 0.0001. Source data are available online for this figure.

Detailed analyses of the genes controlled by VcdR indicated 8 potential target genes, 6 which were downregulated (*ptsG*, *ptsH*, *ptsI*, *nagE*, *treB*, and *vc0177*) and 2 which were upregulated (*lamB* and *vc1779*) (Appendix Table S1). Interestingly, *ptsG*, *nagE*, and *treB* all encode PTS transporters, whereas *ptsH* and *ptsI* encode phosphocarrier proteins, which transfer phosphate to the PTS transporters. To test post‐transcriptional control of these transcripts by VcdR, we cloned their respective 5′ UTRs and translation initiation regions (TIRs) into a GFP‐based reporter plasmid designed to score post‐transcriptional control (Hoyos *et al*, 2020). Of note, *ptsH* and *ptsI* form an operon (Papenfort *et al*, 2015), and thus, we only included the *ptsH* 5′ UTR and TIR in these assays. Co‐transformation of these plasmids with a VcdRP over‐expression vector or a control plasmid in *E. coli* confirmed post‐transcriptional repression of *ptsG*, *nagE*, *treB*, and *ptsHI*; however, we were unable to validate direct regulation of *vc0177*, *lamB*, and *vc1779* (Fig 4A–D and Appendix Fig S2). Interestingly, introducing the STOP codon in the *vcdP* ORF did not change target repression, whereas over‐expression of VcdP failed to repress the reporters (Fig 4A–D).

**Figure EV3:**
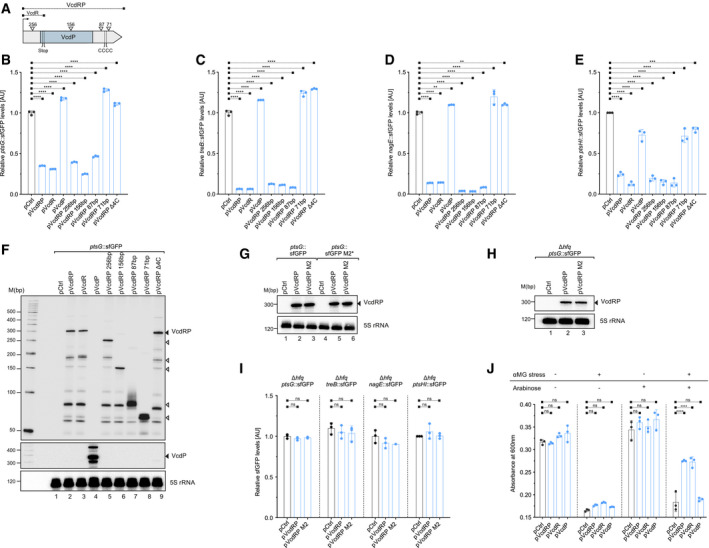
Target spectrum of and post‐transcriptional regulation by VcdR (related to Fig 4) AA schematic representation of the different truncation plasmids of VcdRP tested in (B‐F). These include pVcdRP, pVcdR, pVcdP, and pVcdRP Δ4C or plasmids truncated from the 5′ end maintaining the terminal 256, 156, 87, and 71 bp of *vcdRP*, respectively.B–ERelative fluorescence intensities of *E. coli* translational reporters of *ptsG* (B), *treB* (C), *nagE* (D), and *ptsHI* (E) fused to *sfGFP* harboring either an empty control vector (pCtrl) or VcdRP expression plasmids described in (A) Cells were grown in LB to OD_600_ of 1.0 and fluorophore production was measured using plate reader (B‐D) or by Western blotting (E). The fluorescence of pCtrl (for each reporter fusion) was set to 1.FRNA samples were harvested in parallel to (B) and the corresponding expression was examined by Northern blotting. The solid triangle represents the band corresponding to the full‐length primary *vcdRP* transcript, whereas the open triangles indicate the sizes of the respective truncations tested in (B). Probing with 5S rRNA confirmed equal loading.GNorthern blot analysis of *E. coli* translational reporter of *ptsG* or the corresponding M2* variant, combined with an empty control plasmid or *vcdRP* expression plasmids (pVcdRP and pVcdRP M2). The solid triangle represents the band corresponding to the full‐length primary *vcdRP* transcript. Probing with 5S rRNA confirmed equal loading.HNorthern blot analysis of a translational reporter of *ptsG* fused to *sfGFP* monitored in *E. coli* cells lacking *hfq*, combined with an empty control plasmid or *vcdRP* expression plasmids (pVcdRP and pVcdRP M2). The solid triangle represents the band corresponding to the full‐length primary *vcdRP* transcript. Probing with 5S rRNA confirmed equal loading.IRelative fluorescence intensities of translational reporters of *ptsG*, *treB*, *nagE*, and *ptsHI* fused to *sfGFP* monitored in *E. coli* cells lacking *hfq*, harboring either an empty control vector (pCtrl) or VcdRP expression plasmids (pVcdRP and pVcdRP M2). Cells were grown in LB to OD_600_ of 1.0, and fluorophore production was measured using plate reader (for *ptsG*, *treB*, and *nagE*) or by Western blotting (for *ptsHI*). The fluorescence of pCtrl (for each reporter fusion) was set to 1.JThe base‐pairing element of the dual regulator confers protection against the sugar analog αMG. *V*. *cholerae* Δ*vcdRP* strain harboring either an empty vector control (pCtrl) or inducible *vcdRP* expression plasmids (pVcdRP, pVcdR, pVcdP on *x*‐axis) was grown to early log phase at which 0.1% αMG and / or 0.2% arabinose was added. Absorbance at 600 nm (*y*‐axis) was measured after 5 h of growth without (−) or with (+) αMG and/or arabinose (indicated in the upper panel). Data information: Data in (B–E) and (I–J) are presented as mean ± SD, *n* = 3 independent biological replicates. Statistical significance was determined using one‐way or two‐way ANOVA and post hoc Dunnett’s multiple comparisons test. The *P*‐value is summarized as follows ‐ ns for *P* > 0.05, ** for *P* ≤ 0.01, *** for *P* ≤ 0.001 and **** for *P* ≤ 0.0001. Source data are available online for this figure.

**Figure EV4:**
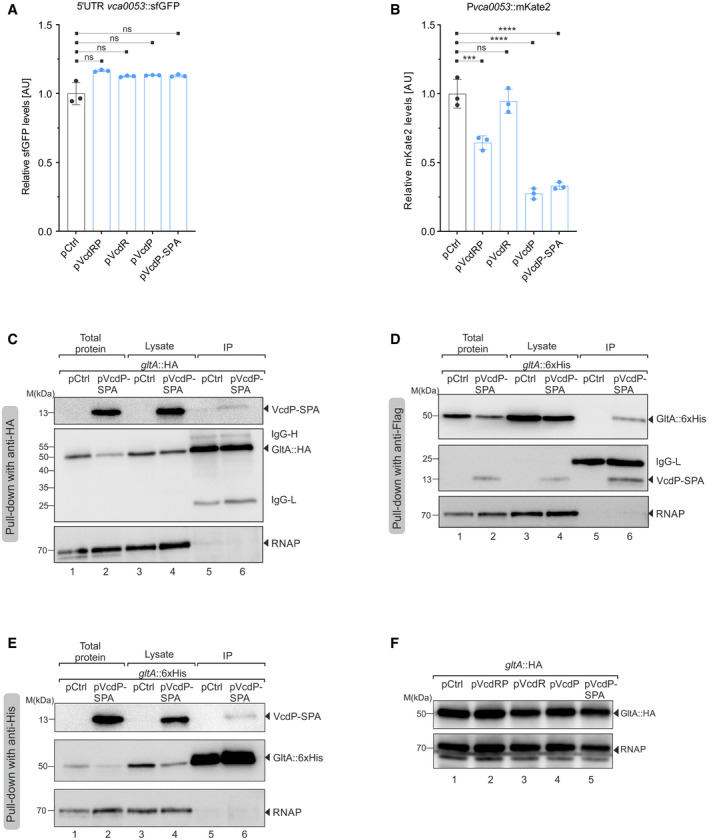
Mechanism of regulation by VcdP and its interaction with citrate synthase (related to Fig 5) A, BRelative fluorescence intensities (*y*‐axis) of *E. coli* translational reporter of *vca0053* fused to *sfGFP* (A) or *V. cholerae* Δ*vcdRP* strain harboring the transcriptional reporter of the *vca0053* promoter fused to *mKate2* (B), combined with either an empty control vector (pCtrl) or VcdRP expression plasmids (pVcdRP, pVcdR, pVcdP, and pVcdP‐SPA). Cells were grown in LB to OD_600_ of 1.0 and the corresponding fluorophore production was measured. The fluorescence of pCtrl (for each reporter fusion) was set to 1.CCo‐immunoprecipitation of chromosomally tagged GltA::HA combined with either an empty vector control (pCtrl) or SPA‐tagged *vcdP* over‐expression plasmid, grown in LB medium to exponential phase (OD_600_ of 0.5). Protein samples corresponding to the total input and cell lysates before and after subjecting to reciprocal immunoprecipitation with anti‐HA antibody. RNAP served as loading control. The solid triangles indicate the corresponding protein sizes.D, ECo‐immunoprecipitation of chromosomally tagged GltA::6xHis combined with either an empty vector control (pCtrl) or SPA‐tagged *vcdP* over‐expression plasmid, subjected to either anti‐Flag (D) or anti‐His (E) as bait. The cultures were grown in LB medium to exponential phase (OD_600_ of 0.5). Western blot analysis for (C–E) with anti‐HA or anti‐His and anti‐Flag antibodies confirmed the interaction of GltA with VcdP *in vivo*. RNAP served as loading control. The solid triangles indicate the corresponding protein sizes.FWestern blot analysis of GltA production (tagged chromosomally with HA), when combined with same set of plasmids in (A) and (B). Protein samples were collected from strains grown in LB medium to mid‐log phase (OD_600_ of 0.5). RNAP served as loading control. Data information: For (A) and (B), data are presented as mean ± SD, *n* = 3 independent biological replicates. Statistical significance was determined using one‐way ANOVA and post hoc Tukey’s multiple comparisons test. The *P*‐value is summarized as follows—ns for *P* > 0.05, *** for *P* ≤ 0.001, and **** for *P* ≤ 0.0001. Source data are available online for this figure.

**Figure EV5:**
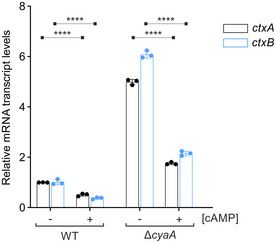
Increased cAMP leads to reduction of *ctxAB* transcript levels (related to Fig 7) *V. cholerae* wild‐type and Δ*cyaA* strains were grown without (−) or with (+) cAMP (f.c. 5 mM) in AKI medium to stimulate production of CTX. Growth under AKI conditions involve biphasic cultures. In the first phase, the cultures were grown in a still tube for 4 h at 37°C. Subsequently, in the second phase, the cultures were poured into a flask to continue growing with shaking. RNA samples equivalent to OD_600_ of 2.0 were harvested after 16 h of continuous shaking followed by qRT analyses of the *ctxA* and *ctxB* transcripts. The fold changes of transcript levels were calculated relative to wild‐type (−) cAMP sample set to 1. *recA* served as the reference housekeeping gene for these measurements. Data information: Data are presented as mean ± SD, *n* = 3 independent biological replicates. Statistical significance was determined using one‐way ANOVA and post hoc Tukey’s multiple comparisons test. The *P*‐values are summarized as follows: **** for *P* ≤ 0.0001. Source data are available online for this figure.

To identify the base‐pairing sequence in VcdRP, we truncated its gene from the 5′ end (maintaining the terminal 256, 156, 87, and 71 bp of *vcdRP*, respectively; Fig EV3A) and tested repression of the PtsG::GFP, NagE::GFP, TreB::GFP, and PtsHI::GFP reporters in *E. coli*. While the 256, 156, and 87 bp variants all repressed GFP production, truncation of *vcdRP* to 71 bp abrogated repression (Fig EV3, EV4, EV5). Hence, we conclude that base pairing with *ptsG*, *nagE*, *treB*, and *ptsHI* requires a VcdR sequence element located in the 3′ end of the transcript. Of note, the 156, 87, and 71 bp VcdRP variants correspond to the endogenous VcdRP isoforms associated with RNase E‐mediated cleavage (Figs EV1A and EV3F), indicating that the 156 and 87 bp processed transcript variants could act post‐transcriptionally to control target gene expression, whereas cleavage at position 71 renders the sRNA inactive.

### Four consecutive cytosines are required for post‐transcriptional regulation by VcdR

To pinpoint the residues relevant for base pairing of VcdR with its targets, we scanned the *vcdRP* alignment (Fig 1C) for conserved sequence elements contained in the 87 bp variant, but missing in the 71 bp regulator. We identified a sequence stretch including residues 223–232 of *vcdRP*, which is conserved in all tested species and characterized by four consecutive cytosines at the distal end (Fig 1C). We thus deleted these four consecutive cytosines in *vcdRP* and analyzed repression of PtsG::GFP, NagE::GFP, TreB::GFP, and PtsHI::GFP. In all cases, this mutation abrogated regulation of the reporters (Fig 4A–D), indicating that VcdRP employs a single conserved base‐pairing site to regulate multiple target mRNAs.

We next employed the RNA hybrid algorithm (Rehmsmeier *et al*, 2004) to predict RNA duplex formation between the sequence containing the four consecutive cytosines in VcdRP and the TIR of *ptsG*, *nagE*, *treB*, and *ptsHI* (Fig 4E–H). Mutation of two of the four cytosines to guanines in VcdRP abrogated repression of all four targets and we were able to compensate this effect by generating a complementary mutation in the targets (Figs 4I–L and EV3G), which validated our base‐pairing predictions. Of note, post‐transcriptional regulation of all 4 target genes was abrogated in Δ*hfq* cells, indicating that Hfq is strictly required for VcdR‐mediated gene control (Fig EV3H and I).

To corroborate these findings at the phenotypic level, we tested growth of *V. cholerae* in the presence of the glucose analog α‐methyl glucoside (αMG). αMG is imported into the cell via the PtsG transporter and inhibits cell growth (Wadler & Vanderpool, 2007; Lloyd *et al*, 2017). We hypothesized that VcdR‐mediated repression of PtsG could suppress this effect and thus promote cell replication. Indeed, we discovered that αMG strongly reduced the growth of *V. cholerae* and this effect was counteracted when VcdRP or VcdR was over‐expressed (Fig EV3J). In contrast, VcdP did not ameliorate growth. In summary, we conclude that the regulatory RNA element in VcdRP inhibits the synthesis of PTS sugar transport proteins, which is mediated by a conserved sequence stretch located in the 3′ part of the regulator.

### VcdP interacts with GltA *in vivo*

The above results indicated that VcdR controls carbon transport genes at the post‐transcriptional level; however, how VcdP affects gene expression remained unclear. To address this question, we focused on *vca0053* (encoding purine nucleoside phosphorylase), which was the most strongly VcdP‐repressed gene in our transcriptomic experiment (˜7‐fold), but did not show regulation by VcdR (Appendix Table S1). Specifically, to study the mechanism underlying VcdP‐mediated regulation of *vca0053*, we generated transcriptional and translational reporters for the gene (*vca0053::mKate2* and *vca0053::gfp*, respectively) and tested activity when VcdRP, VcdR, or VcdP were over‐expressed. Whereas we did not observe significant regulation of the translational reporter in response to any of the variants (Fig EV4A), VcdRP and VcdP inhibited the production of *vca0053::mKate2* (Fig EV4B). In accordance with our transcriptomic analyses (Appendix Table S1), VcdR failed to repress this reporter. Importantly, we also tested regulation of both reporters by VcdP carrying a C‐terminal SPA epitope (VcdP::SPA). Again, this variant inhibited *vca0053::mKate2* activity, but did not affect *vca0053::gfp*, suggesting that the SPA tag did not interfere with VcdP activity (Fig EV4A and B).

**Figure 5:**
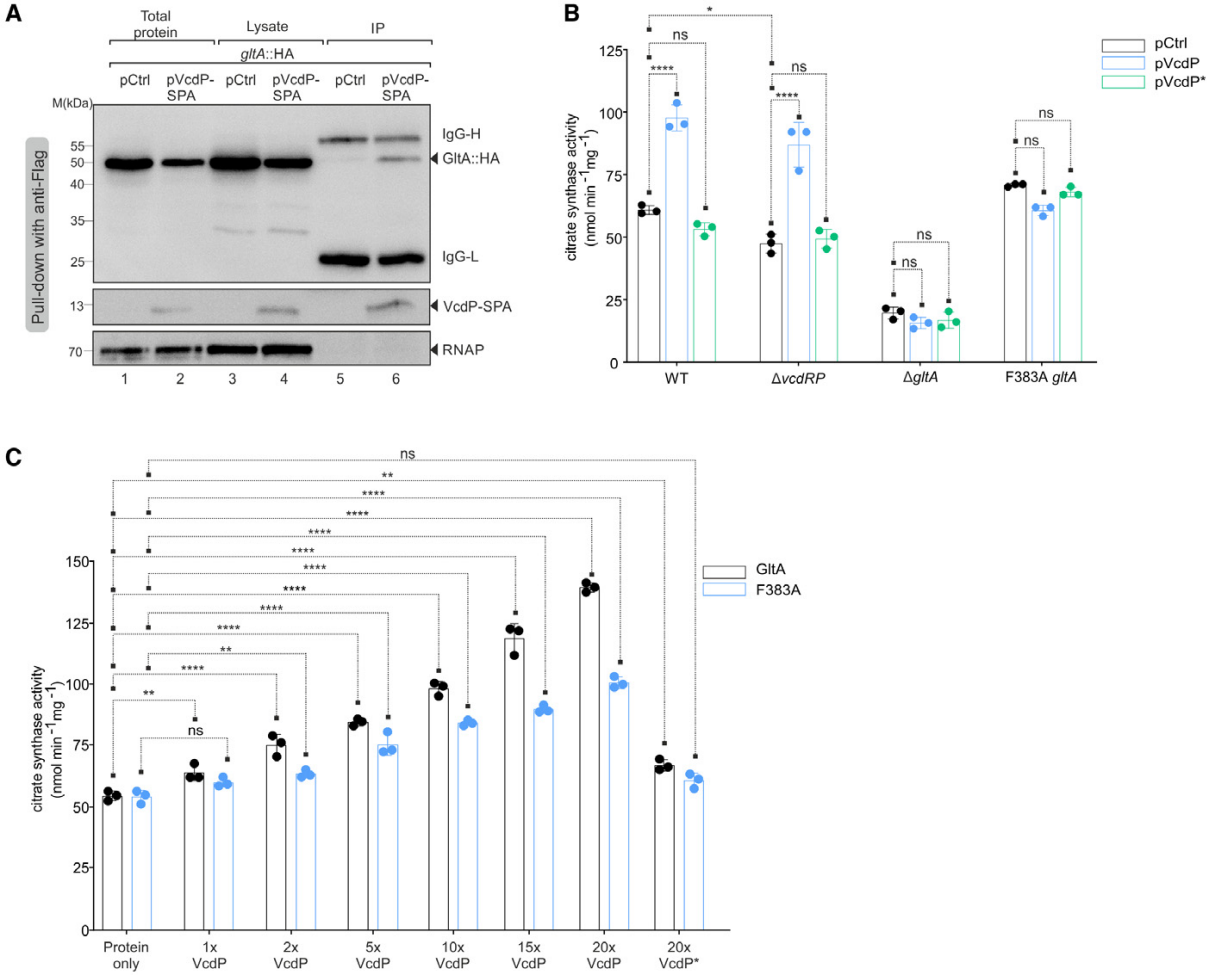
VcdP interacts with and increases the activity of citrate synthase AImmunoprecipitation of chromosomally tagged GltA::HA combined with either an empty vector control (pCtrl) or SPA‐tagged *vcdP* over‐expression plasmid, grown in LB medium to exponential phase (OD_600_ of 0.5). The SPA epitope contains the 3×FLAG and the calmodulin binding peptide sequences separated by a TEV protease cleavage site (Zeghouf *et al*, 2004). Therefore, protein samples corresponding to the total input and cell lysates before and after subjecting to immunoprecipitation with anti‐Flag antibody were loaded on an SDS–PAGE. Western blotting with anti‐HA and anti‐Flag antibodies confirmed the interaction of GltA with VcdP *in vivo*. RNAP served as loading control. The solid triangles indicate the corresponding protein sizes.BCitrate synthase enzyme activity measurements performed on cellular lysates. An empty vector control (pCtrl) or *vcdP* expression plasmids (pVcdP and pVcdP*) were conjugated into *V. cholerae* wild‐type, Δ*vcdRP*, Δ*gltA*, or F383A GltA backgrounds. Cells were grown in LB medium to stationary phase and subsequently lysed. The protein concentrations of these cellular extracts were measured and served as input for the assay. The resulting colorimetric product at 412 nm was proportional to the enzymatic activity of citrate synthase present. The enzyme kinetics were assayed at 15‐s intervals for at least 30 min and the activity per milligram of lysate was determined from the initial velocities.CCitrate synthase enzyme activity measurements performed *in vitro*. *V. cholerae* GltA and F383A GltA proteins were purified using a 6xHis tag in *E. coli*. Both the proteins were treated without the synthesized VcdP or VcdP* peptide, or with increasing concentrations of the two peptides (1, 2, 5, 10, 15, or 20× with respect to the protein monomer). The citrate synthase activity per milligram of protein as a result of its interaction with the peptide was determined from the initial velocities. Data information: Data in (B and C) are presented as mean ± SD, *n* = 3 independent biological replicates. Statistical significance was determined using one‐way ANOVA and post hoc Tukey’s or Dunnett’s multiple comparisons test. The *P*‐value is summarized as follows ‐ ns for *P* > 0.05, * for *P* ≤ 0.05, ** for *P* ≤ 0.01, and **** for *P* ≤ 0.0001. Source data are available online for this figure.

To identify cellular interaction partners of VcdP in *V. cholerae*, we over‐expressed VcdP::SPA in *V. cholerae*. The SPA epitope contains the 3×FLAG and the calmodulin‐binding peptide sequences separated by a TEV protease cleavage site (Zeghouf *et al*, 2004), and therefore, we co‐immunoprecipitated cellular proteins using an anti‐FLAG antibody. We performed mass spectrometry analysis of the bound proteins, which we compared to an isogenic strain carrying a control plasmid. When analyzing three biological replicates of this experiment, we discovered only a single protein, GltA (citrate synthase), that was detected in all VcdP::SPA samples, but not in the control (Appendix Table S2). To validate this potential interaction, we cloned an HA epitope to the C‐terminus of the chromosomal *gltA* gene in *V. cholerae* and repeated the co‐immunoprecipitation experiment. Indeed, we recovered GltA::HA when using VcdP::SPA as bait (Fig 5A). Reciprocally, we detected VcdP::SPA when GltA::HA was the bait (Fig EV4C). We also exchanged the HA‐tag on GltA to 6xHIS and were able to confirm interaction of GltA with VcdP (Fig EV4D and E). Of note, over‐expression of VcdP::SPA did not affect GltA abundance, indicating that VcdP affects GltA activity, rather than synthesis or turn‐over of the protein (Fig EV4F).

### VcdP facilitates GltA activity

Citrate synthase (GltA) is the first enzyme of the citric acid cycle and catalyzes the irreversible condensation of acetyl coenzyme A (acetyl‐CoA) with oxaloacetate to form citrate and CoA with a thiol group (CoA‐SH). To test whether VcdP modulates GltA function, we employed a colorimetric citrate synthase activity assay. Here, CoA‐SH reacts with DTNB (5,5′‐dithiobis‐(2‐nitrobenzoic acid)) to form TNB, which exhibits absorbance at 412 nm (Kanellopoulos *et al*, 2020). Cell extracts were obtained from *V. cholerae* wild‐type and Δ*vcdRP* cells carrying either a control vector, the VcdP plasmid, or a VcdP plasmid in which amino acids 15–18 of the protein were mutated to alanine (VcdP*; Appendix Fig S3). Over‐expression of VcdP led to an increase in citrate synthase activity and we obtained similar results in the Δ*vcdRP* background (Fig 5B). In contrast, VcdP* did not activate citrate synthase. Interestingly, comparison of citrate synthase activity in wild‐type and Δ*vcdRP* cells both harboring the control plasmid revealed mildly reduced enzyme activity in the *vcdRP* mutant, suggesting that chromosomal VcdP production also facilitates GltA activity (Fig 5B). We also tested the impact of VcdP over‐expression on *V. cholerae* cells lacking the *gltA* gene. As expected, deletion of *gltA* strongly reduced the activity of the lysates and VcdP over‐expression did not affect this pattern. Of note, the *V. cholerae* genome encodes a GltA paralog (VC1337; Heidelberg *et al*, 2000), which might account for the residual citrate synthase activity in the assay (Fig 5B).

GltA from Gram‐negative bacteria, such as *V. cholerae*, is a so‐called type II citrate synthase, which forms hexamers (trimer of dimeric subunits) and, in contrast to citrate synthase from animals, plants, and other bacteria, is allosterically inhibited by NADH (Weitzman & Jones, 1968; Nguyen *et al*, 2001). We thus speculated that VcdP could affect GltA activity by counteracting the inhibition of the enzyme by NADH. To test this hypothesis, we employed a previously studied mutant of GltA in which a phenylalanine residue at position 383 of the protein was changed to alanine (F383A). This GltA variant is capable of binding NADH; however, its enzymatic activity is not inhibited by the cofactor (Pereira *et al*, 1994; Nguyen *et al*, 2001; Maurus *et al*, 2003; Stokell *et al*, 2003). We thus introduced this mutation in the chromosomal *gltA* gene of *V. cholerae* and monitored citrate synthase activity. In contrast to wild‐type GltA, the F383A variant was no longer activated by VcdP over‐expression (Fig 5B), suggesting that VcdP might suppress the inhibitory effect of NADH on GltA.

We next aimed to study the impact of VcdP on GltA *in vitro*. To this end, we purified *V. cholerae* 6xHIS::GltA in *E. coli* and tested enzyme activity. Indeed, the enzyme was readily active in solution (Fig 5C). In accordance with our previous measurements (Fig 5B), addition of chemically synthesized VcdP protein increased GltA activity in a concentration‐dependent manner (Fig 5C). VcdP also activated the purified F383A variant; however, this effect was significantly weaker, when compared to wild‐type GltA. Likewise, we observed that chemically synthesized VcdP* (analogous to Fig 5B and Appendix Fig S3) was unable to efficiently activate either GltA wild‐type protein, or the F383A mutant (Fig 5C). Together, we conclude that VcdP binds to and activates GltA *in vivo* and *in vitro*, which might also affect the interaction of GltA with NADH.

### VcdRP modulates central metabolism in *V. cholerae*

**Figure 6:**
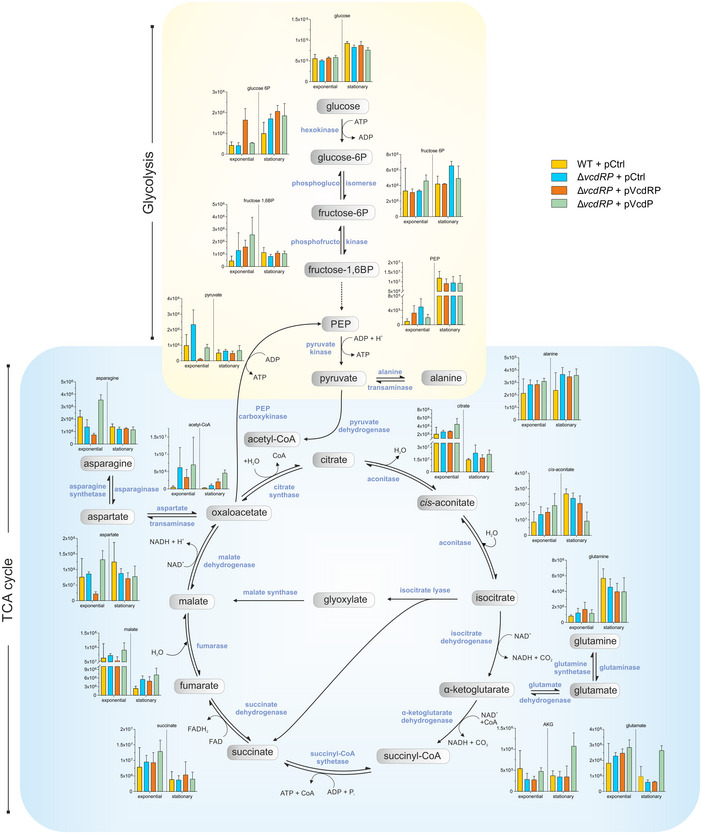
Glycolytic and citric acid cycle metabolites are regulated by VcdR/P Metabolite abundance of glycolytic and citric acid cycle intermediates in exponential (OD_600_ of 0.5) and stationary phase (OD_600_ of 3.0) of growth were measured for *V. cholerae* wild‐type and Δ*vcdRP*, each harboring an empty vector control (pCtrl) or Δ*vcdRP* with *vcdRP* expression plasmids (pVcdRP and pVcdP). The *y‐axis* represents the peak area of each metabolite determined by mass spectrometry. The main operative pathways including the fates of certain amino acids are color‐coded (yellow for glycolysis and blue for citric acid cycle). Data information: Data are presented as mean ± SEM, *n* = 3 independent biological replicates. glucose‐6P (glucose‐6‐phosphate), fructose‐6P (fructose 6‐phosphate), fructose‐1,6BP (fructose 1,6‐bisphosphate), PEP (phosphoenolpyruvate). Source data are available online for this figure.

As the first enzyme of the citric acid cycle, GltA is involved in the generation of various metabolic intermediates including reduced purine nucleotides that are used for energy generation through oxidative phosphorylation. We thus investigated how VcdRP affects the metabolic profile of *V. cholerae* cultivated to exponential or stationary growth phase. Specifically, we employed mass spectrometry to record key metabolites of the glycolysis pathway and citric acid cycle in wild‐type and Δ*vcdRP* cells (both carrying a control plasmid), as well as in Δ*vcdRP V. cholerae* expressing either VcdRP or VcdP from a plasmid (Fig 6). For the glycolysis pathway, we discovered that lack of *vcdRP* resulted in elevated pyruvate levels under exponential growth conditions, while over‐expression of VcdRP had the inverse effect and at the same time also resulted in increased amounts of glucose‐6‐phosphate. In contrast, over‐expression of VcdP alone did not affect glycolysis intermediates, supporting the hypothesis that VcdR modulates carbohydrate uptake and consumption, whereas VcdP acts further downstream in the metabolic cascade. Indeed, analysis of metabolites belonging to the citric acid cycle revealed elevated α‐ketoglutarate levels in stationary phase cells overproducing VcdP, whereas the precursor intermediate *cis*‐aconitate showed the inverse pattern. Increased concentrations of α‐ketoglutarate in VcdP expressing cells also resulted in higher L‐glutamate levels, which can be produced from α‐ketoglutarate through the activity of glutamate dehydrogenase (Reitzer, 2004). For comparison, we also obtained metabolic profiles of Δ*gltA V. cholerae* cells carrying the pVcdP plasmid or a control plasmid (Appendix Fig S4). Importantly, in the absence of *gltA*, VcdP did not change levels of the levels of α‐ketoglutarate and glutamate, or any other intermediate of the citric acid cycle. These results are in line with our idea that VcdP acts through GltA to modulate central metabolism.

## Discussion

**Figure 7:**
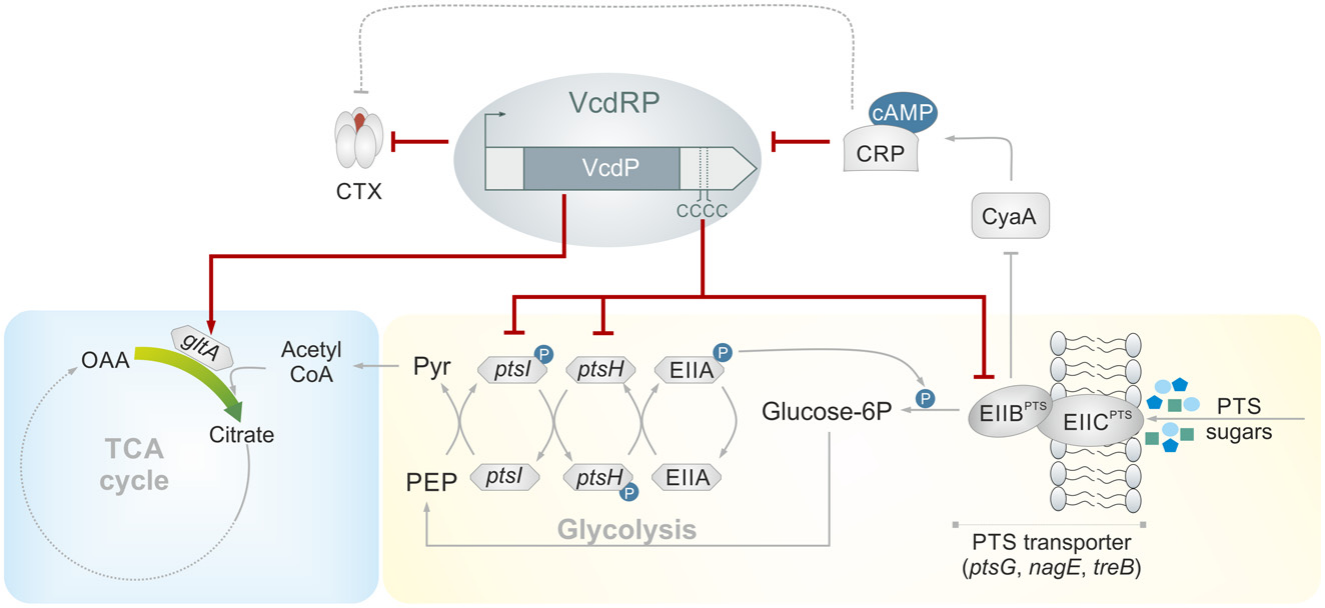
Model depicting the dual function of VcdRP in balancing carbon metabolism VcdRP is a dual‐function regulator that represses CTX production in *V. cholerae*. Additionally, CRP and cAMP act on the regulator at the transcriptional level and suppress the production of VcdRP. The sRNA element (VcdR) acts via a conserved stretch of four consecutive cytosines to base pair with the translation initiation sites of PTS‐specific and non‐specific sugars, resulting in the inhibition of carbon uptake. The peptide VcdP binds to and increases the activity of citrate synthase enzyme, thereby modulating the levels of metabolites within the cycle. Thus, VcdR and VcdP together synchronize the carbon uptake and its subsequent utilization, thereby balancing carbon metabolism.

The glycolytic pathway and the citric acid cycle are ubiquitous in nature. Although the enzymes involved in these pathways have long been known, their regulation is not fully understood. Furthermore, much of the knowledge regarding the regulation of the citric acid cycle and glycolysis in bacteria stems from model organisms such as *E. coli* and *Bacillus subtilis*; however, it is becoming ever more clear that there are significant species‐specific differences (Orlenko *et al*, 2016). Similarly, it is crucial that the regulatory mechanisms of the major metabolic pathways are interconnected to provide a global, robust, and economical response to changing environmental conditions. Here, we identified the VcdRP dual‐function RNA of *V. cholerae* as a modulator of both the citric acid cycle and the glycolysis pathway employing either the VcdP protein or the VcdR base‐paring regulator, respectively (Fig 7).

Transcriptional control of *vcdRP* is mediated by CRP, which inhibits *vcdRP* production (Fig 2). CRP directly controls dozens of additional genes in *V. cholerae* and is key for virulence in multiple models of infection (Skorupski & Taylor, 1997; Liang *et al*, 2007; Manneh‐Roussel *et al*, 2018). Similarly, PtsI and PtsH are required for virulence in *V. cholerae* and mutants lacking either of the two genes display strongly decreased levels of TcpA and CTX (Merrell *et al*, 2002; Wang *et al*, 2015). Given that VcdR base pairs to and inhibits the expression of *ptsHI* mRNA (Fig 4A and E), these results might well explain the reduced production of CTX in cells over‐expressing VcdRP (Fig 1A). The exact molecular mechanism of how PtsHI affects virulence genes in *V. cholerae* is currently unknown; however, it has been speculated that mutation of *ptsH* or *ptsI* results in elevated cAMP levels (Wang *et al*, 2015), which activates CRP. The CRP‐cAMP complex then inhibits the expression of the *tcpPH* operon, whose gene products are an integral part of *V. cholerae*’s virulence cascade and required for CTX expression. Indeed, we discovered that mutation of *cyaA* resulted in ˜5‐fold and ˜6‐fold increased *ctxA* and *ctxB* mRNA levels, respectively, whereas external addition of cAMP reduced *ctxA*B production in wild‐type and Δ*cyaA* cells (Fig EV5). Of note, mutation of PTS transporters (including PtsG, TreB, and NagE) did not affect virulence in *V. cholerae* (Hayes *et al*, 2017), suggesting that repression of *ptsHI* is most critical for CTX down‐regulation by VcdR (Fig 3B).

In contrast to the effect of overproducing VcdR or VcdRP, CTX levels remained unaffected when only VcdP was over‐expressed (Fig 3B). However, similar to PtsHI, the target of VcdP, GltA, has also been implicated in virulence of *V. cholerae*. Interestingly, *V. cholerae* cells lacking *gltA* displayed impaired survival in infant rabbits and upon transmission into pond water, but remained capable of infecting mice (Kamp *et al*, 2013). Thus, GltA could be a host‐specific virulence factor, suggesting differences in carbon utilization and citric acid cycle activity in the two hosts.

In *B*. *subtilis* and *Vibrio natriegens*, citric acid cycle intermediates determine the production of the biotechnologically important storage polymers poly‐γ‐glutamic acid and poly‐β‐hydroxybutyrate, respectively (Dalia *et al*, 2017; Halmschlag *et al*, 2020). Therefore, we tested whether VcdP from *V. cholerae* could also increase GltA activity in these two organisms. VcdP over‐production readily upregulated GltA activity in *V. natriegens*, whereas it did not affect GltA of *B. subtilis* (Appendix Fig S5A and B). These results suggest that VcdP‐mediated activation of citrate synthase might be specific to the hexameric GltA complex of Gram‐negative bacteria, whereas dimeric GltA complexes of Gram‐positive organisms do not interact with VcdP. This hypothesis is further supported by our finding that the GltA F383A variant of *V. cholerae* is not activated by VcdP (Fig 5B). In comparison with wild‐type GltA, F383A is not inhibited by NADH, a feature that is limited to hexameric type II citrate synthases of Gram‐negative bacteria (Weitzman & Jones, 1968; Nguyen *et al*, 2001). The molecular mechanisms underlying the activation of GltA by VcdP are an important direction for future research.

Despite being a potentially useful regulator for biotechnology applications, the interplay of VcdR and VcdP in the cell is also an interesting question. Indeed, we discovered that pulse expression of VcdRP modulated the expression of significantly more genes (103) than the sum of genes affected by either VcdR (49 genes) or VcdP (8 genes; Fig 3C and Appendix Table S1). Overall we found that regulation of genes activated by VcdP is more pronounced when VcdR is co‐expressed. For example, expression of *lamB* increased ˜2‐fold with VcdR, ˜4.4‐fold with VcdP, and ˜10‐fold with VcdRP and we observed a similar trend for several additional genes involved in carbon metabolism including *glpK*, *mglC*, *potE*, *rbsC*, and *sucC* (Appendix Table S1). Therefore, the physiological role of VcdP might only become fully evident in combination with VcdR and *vice versa*.

One interesting clue that could further help to understand the interplay of VcdR and VcdP in the cell comes from a long‐term evolution experiment, *i.e*., the Lenski experiment, covering more than 25 years of adaptation in *E. coli* (Blount *et al*, 2018). Approximately 15 years into the experiment, *E. coli* obtained a mutation in *gltA* which alleviated repression of citrate synthase by NADH and thus increased enzyme activity. This mutation was preceded by two additional mutations in citrate transporter genes and together allowed the cells to utilize citrate as a carbon source under aerobic conditions (Quandt *et al*, 2015). However, later in the evolution process, additional mutations in *gltA* occurred, which led to reduced protein abundance, suggesting that high GltA activity can be beneficial under certain conditions but comes with an overall reduction in fitness. We therefore speculate that VcdP might provide a transient increase in citrate synthase activity and that VcdR might compensate for the associated negative fitness effects. Indeed, high citrate synthase activity seems to be mostly detrimental when cells use glucose as a carbon source (Quandt *et al*, 2015) and the inhibition of PTS transporters and PtsHI by VcdR could help to balance the amount of carbohydrates coming into the cell (Fig 7).

This functional interdependency is also reflected in the conservation of the *vcdRP* gene. In all tested species, the VcdR base‐pairing sequence and the *vcdP* ORF were conserved (Fig 1B and C). This is contrary to other dual RNAs, such as the well‐studied SgrST, in which the base‐pairing sequence is highly conserved whereas the *sgrT* ORF is only found in a subset of species (Horler & Vanderpool, 2009). The regulatory function of SgrST shows several interesting analogies with VcdRP. For example, VcdR and SgrS both inhibit the expression of PTS‐encoding mRNAs and both base‐pairing sequences are positioned in the distal end of their transcripts with the small ORF located upstream of this sequence (Fig 1C; Wadler & Vanderpool, 2007). Indeed, both SgrS and VcdR protect cells from the toxic effect of αMG (Fig EV3J; Vanderpool & Gottesman, 2004). However, whereas SgrT adds to this protection from carbohydrate accumulation by inhibiting the activity of PtsG transporter (Lloyd *et al*, 2017), VcdP modulates citric acid cycle flux by promoting the activity GltA (Fig 5B and C). Likewise, the two regulators differ in their transcriptional control: expression of *sgrST* is activated by the sugar‐phosphate stress‐specific transcription factor SgrR (Vanderpool & Gottesman, 2007), while synthesis of *vcdRP* is repressed by the globally acting transcriptional regulator, CRP (Fig 2).

At least three additional RNA regulators have been reported to be directly controlled by CRP: CyaR, which is activated by CRP (Johansen *et al*, 2008; Papenfort *et al*, 2008; De Lay & Gottesman, 2009) and Spot 42 and AzuCR (a.k.a. IsrB), which are both repressed by the regulator (Polayes *et al*, 1988; Hemm *et al*, 2010). Spot 42 arguably belongs to the most thoroughly characterized post‐transcriptional regulators and has been documented to facilitate the transition between carbon sources in *E. coli* (Beisel & Storz, 2011). AzuCR was initially discovered as an sRNA candidate and subsequently reported to encode a small protein (Hemm *et al*, 2008). A recent study by the Storz group (see Raina *et al*, co‐submitted with this manuscript) now found that AzuCR indeed acts as a dual RNA and inhibits the expression of the *cadA* and *galE* mRNAs through a base‐pairing mechanism, while the AzuC small protein interacts with and up‐regulates the activity of aerobic glycerol 3‐phosphate dehydrogenase, GlpD. We currently do not know why non‐coding regulators and dual‐function RNAs are frequently found to be involved in pathways associated with carbon uptake and metabolism in bacteria (Durica‐Mitic *et al*, 2018). However, one possible interpretation could be that carbon metabolism provides a strong selective pressure requiring a rapid and multilayered regulatory response, which might well be most efficiently achieved by sRNAs and dual‐function RNAs.

## Materials and Methods

### Bacterial strains and growth conditions

A complete list of strains used in this study is provided in Appendix Table S3. *Vibrio cholerae* C6706 was used as the wild‐type strain and all the mutant strains were generated as described previously (Peschek *et al*, 2019). Briefly, RK2/RP4‐based conjugal transfer was used to introduce pKAS32‐derived plasmids from *E. coli* S17λpir plasmid donor strains. Subsequently, trans‐conjugants were selected for ampicillin (200 mg/ml) resistance. Polymyxin B (6.25 mg/ml) was used to specifically inhibit *E. coli* growth. Subsequently, single colonies were transferred to fresh plates and selected for streptomycin (5,000 mg/ml) resistance. Finally, mutants were confirmed by PCR and sequencing. *B. subtilis* 168 and *V. natriegens* ATCC14048 served as the wild‐type hosts for cloning and plasmid transformation as described previously (Brockmeier *et al*, 2006; Weinstock *et al*, 2016). All bacterial strains were grown at 37°C in LB broth (Lennox, Carl‐Roth, Karlsruhe, Germany) or M9 minimal medium (supplemented with 0.4% casaminoacids and 0.4% glucose or glycerol) or in AKI medium (0.5% sodium chloride, 0.4% yeast extract and 1.5% bacto‐peptone, 0.3% sodium bicarbonate). *V. natriegens* was grown in LB broth supplemented with 5% sodium chloride.

### Plasmids and DNA oligonucleotides

The plasmids and DNA oligonucleotides used in this study are listed in Appendix Table S4 and S5, respectively. The plasmids for the over‐expression sRNA library to screen for CTX repression were cloned into pEVS143 (Dunn *et al*, 2006) linearized with KPO‐0092/1397 followed by digestion with XbaI enzyme and subsequent ligation via T4 ligase with the corresponding sRNA inserts. For the amplification of the sRNAs, KPS‐0014 chromosomal DNA served as a template with the following combinations of oligonucleotides: KPO‐1076/1077 (pAS001), KPO‐1072/1073 (pAS002), KPO‐1070/1071 (pAS003), KPO‐0456/0465 (pKP333), KPO‐1003/1004 (pNP001), KPO‐0999/1000 (pNP002), KPO‐1024/1025 (pNP003), KPO‐1005/1006 (pNP004), KPO‐1015/1016 (pNP005), KPO‐1021/1022 (pNP006), KPO‐1011/1012 (pNP007), KPO‐1009/1010 (pNP008), KPO‐1219/1220 (pNP009), KPO‐1001/1002 (pNP010), KPO‐1007/1008 (pNP011), KPO‐1026/1027 (pNP012), KPO‐1013/1014 (pNP015), KPO‐1082/1083 (pRH001), KPO‐1092/1093 (pRH002), KPO‐1090/1091 (pRH003), KPO‐1860/1861 (pSG002), and KPO‐1448/1449 (pYH002). The remaining plasmids for the sRNA library screen were cloned into pEVS143 (Dunn *et al*, 2006) linearized with KPO‐0092/1397, with the corresponding sRNA inserts amplified from KPS‐0014 chromosomal DNA, followed by Gibson assembly. These sRNAs were amplified using KPS‐0014 chromosomal DNA as a template with the following combinations of oligonucleotides: KPO‐2568/2553 (pMD090), KPO‐2570/2565 (pMD104), KPO‐2571/2572 (pMD105), KPO‐1375/1376 (pRH0009), and KPO‐1383/1384 (pRH010). The inserts for *vcdRP* truncation plasmids were amplified with KPO‐2082/2083 (pSG006), KPO‐2082/2085 (pSG008), KPO‐2082/2109 (pMD062), and KPO‐2082/2110 (pMD063) and cloned into pEVS143 linearized using KPO‐1949/0092 by Gibson assembly. Site‐directed mutagenesis of pNP009 using KPO‐2090/2091 and KPO‐6476/6477 yielded pMD055 and pMD389, respectively. pMD083 was generated from pNP009 by amplifying the plasmid in two fragments with KPO‐1529/2323 and KPO‐1525/2322, respectively, and subsequently joined by Gibson assembly. For pMD004, the *rrnB* terminator from pKP8‐35 (Papenfort *et al*, 2006) was amplified with KPO‐1484/1485 and cloned by Gibson assembly into pKP‐331 (Papenfort *et al*, 2015) linearized with KPO‐0196/1397. pMD072 was generated by amplifying the insert from pNP009 using KPO‐2100/2101 and cloned via Gibson assembly into pMD004 linearized with KPO‐0196/1397. Site‐directed mutagenesis of pMD072 using KPO‐2090/2091 yielded pMD077. For pMD080, an artificial 5’UTR, MCS, and T1 terminator were inserted via overlapping primers using KPO‐2259/2260/2261/2329/1492 and cloned into pEVS143 (Dunn *et al*, 2006) linearized with KPO‐0092/1949. For pMD087, the codon‐modified VcdP sequence was custom synthesized by GeneArt and cloned using Gibson assembly into pMD004 linearized with KPO‐2391/2392. The insert fragment for pMD111 was amplified from pMD087 with KPO‐2330/1484 and cloned into pMD080 linearized with pBAD‐ATGrev/KPO‐1397. The SPA tag was inserted using KPO‐1715/4716 into linearized pMD065 with KPO‐4168/2410 to yield pKV114. The translational GFP fusions were cloned as previously described (Corcoran *et al*, 2012) by employing already determined transcriptional start site annotations (Papenfort *et al*, 2015). Briefly, *treB* (pNP058), *ptsG* (pMD161), *nagE* (pMD0162), and *ptsHI* (pMD164) inserts were amplified from KPS‐0014 chromosomal DNA using the primers indicated in Appendix Table S5 and introduced into the pXG‐10SF backbone using NheI and NsiI restriction sites or via Gibson assembly. The M2* mutation was introduced via site‐directed mutagenesis into the translational GFP fusions. To this end, amplification with KPO‐6560/6561, KPO‐6480/6481, KPO‐6562/6563, and KPO‐6558/6559 yielded pMD401, pMD402, pMD403, and pMD405, respectively. VcdRP transcriptional mKate2‐based reporter (pMD064) was generated by amplifying KPS‐0014 chromosomal DNA using KPO‐2111/2112 and ligating into pYH010 digested with SphI and SalI enzymes, via T4 ligase. Site‐directed mutagenesis of pMD064 with KPO‐6954/6955 yielded pKV164. All pKAS32‐derived plasmids (Skorupski & Taylor, 1996) were constructed by linearizing the backbone using KPO‐0267/0268 and cloning the corresponding “up” and “down” homologous fragments by Gibson assembly. An additional flank containing the SPA tag sequence where appropriate was also included. For the amplification of the “up” and “down” flanking fragments, KPS‐0014 chromosomal DNA served as a template with the following combinations of oligonucleotides for each of the flanking fragments: pMD054 (KPO‐1282/1283 and KPO‐1284/1285), pRH023 (KPO‐2243/2244 and KPO‐2245/2246), pRH024 (KPO‐2249/2250 and KPO‐2251/2252), pKV150 (KPO‐6306/6307 and KPO‐6308/6309), pKV154 (KPO‐6524/6525 and KPO‐6526/6527), pKV155 (KPO‐6306/6670 and KPO‐6309/6669), and pKV157 (KPO‐6472/6473 and KPO‐6744/6745 along with KPO‐4814/6178 for SPA tag amplified from pKV114). Site‐directed mutagenesis of pKV150 using KPO‐6484/6485 and KPO‐6486/6487 yielded pKV152 and pKV153, respectively. Plasmid pMD408 was constructed by cloning *gltA* amplified from KPS‐0014 chromosomal DNA using KPO‐6588/6589 into pET15b (Novagen) linearized with KPO‐4202/4203. Site‐directed mutagenesis of pMD408 using KPO‐6669/6670 yielded pKV156. pKV169 was generated by Gibson assembly of linearized pBSmul2 (pKV168) using KPO‐7127/7130 and insert amplified from pMD111 using KPO‐7131/7132. The insert fragment for pKV175 and pKV176 were amplified from KPS‐0014 chromosomal DNA using KPO‐7413/7414 and 7415/7416, respectively, and cloned via Gibson assembly into linearized pMD080 (with KPO‐3236/pBAD‐ATGrev) and pMD004 (with KPO‐0196/1488), respectively.

### RNA isolation and Northern blot analysis

Total RNA was prepared and blotted as described previously (Peschek *et al*, 2019). Briefly, total RNA samples corresponding to 4 OD_600_ units were harvested and mixed with 0.2 volumes of “stop‐mix” (95% ethanol, 5% phenol). Cell pellets were obtained by centrifugation, thoroughly resuspended in 1ml of EXTRAzol, and the samples were incubated at room temperature for 5 min. Subsequently, they were transferred to phase lock gel™ tubes, 200 µl of chloroform was added, mixed by inversion, and incubated for 5 min at room temperature to allow phase separation. After the samples were centrifuged at maximal speed for 15 min at 12°C, the upper phase was transferred to a new tube and 400 µl of isopropanol was added. After incubating at room temperature for 30 min, the samples were centrifuged at maximal speed for 30 min at 4°C. Following two washes with 75% ethanol, the pellets were resuspended in 20–30 µl of nuclease‐free water and quantified using a NanoDrop (Thermo Fisher Scientific). 5–10 µg RNA was then loaded on a 6% polyacrylamide 5 M urea gel and subsequently blotted on Amersham Hybond XL membranes (Cytiva Life Sciences, Marlborough, Massachusetts). The membranes were then hybridized in Roti‐Hybri‐Quick buffer (Carl Roth, Karlsruhe, Germany) at 42°C with (γ‐^32^P)‐ATP end‐labeled DNA oligonucleotides listed in Appendix Table S5. Signals were visualized using a Typhoon Phosphorimager (GE Healthcare, Chicago, Illinois).

### Determination of RNA stability

To determine transcript stability, cultures were grown in biological triplicates to appropriate growth phases when transcription was inhibited by the addition of rifampicin (f.c. 250 μg/ml). Prior to, as well as at 2, 4, 8, 16 and 32 min post‐rifampicin treatment, culture aliquots were mixed with 0.2 vol. equiv. stop‐mix (95% ethanol, 5% phenol) and samples were frozen in liquid nitrogen. RNA was extracted, and examined on Northern blots.

### Western blot analysis

Total protein samples corresponding to 1 OD_600_ units were collected at the desired OD_600_ and cell pellets were resuspended in 1× Laemmli buffer to a final concentration of 0.01 OD per µl. The samples were immunoblotted as previously described (Papenfort *et al*, 2015). Signals were visualized using a Fusion FX EDGE imager and band intensities were quantified using the BIO‐1D software (both from Vilber Lourmat, Marne‐la‐Vallée, France). 3xFlag and SPA‐tagged proteins were detected using mouse anti‐Flag antibody (#F1804, Sigma‐Aldrich, St. Louis, Missouri). The SPA epitope contains the 3×FLAG and the calmodulin‐binding peptide sequences separated by a TEV protease cleavage site (Zeghouf *et al*, 2004). HA and 6xHis‐tagged samples were detected using mouse monoclonal anti‐HA (#ab18181, Abcam, Cambridge, UK) and rabbit monoclonal anti‐6xHis (#ab200537, Abcam, Cambridge, UK) antibodies, respectively. RNAP served as a loading control and was detected using rabbit anti‐RNAP antibody (#WP003, BioLegend, San Diego, California). The corresponding secondary antibodies used were goat anti‐mouse HRP‐conjugated IgG antibody, (#31430, Thermo Fisher Scientific, Waltham, Massachusetts) and goat anti‐rabbit HRP‐conjugated IgG antibody, (#16104, Thermo Fisher Scientific, Waltham, Massachusetts).

### Fluorescence reporter measurements

Fluorescence assays of bacterial reporters were performed as previously described (Corcoran *et al*, 2012). Briefly, cell pellets corresponding to 1 OD_600_ units were collected by centrifugation. Subsequently, the pellets were washed twice in equal volume of 1×PBS (pH 7.4) and fluorescence intensity was quantified using a Spark 10 M plate reader (Tecan, Männedorf, Switzerland). Control strains not expressing fluorescent proteins were used to subtract background autofluorescence.

### Genetic screen for CTX repression

A sRNA over‐expression library was screened in a *V. cholerae* C6706 Δ*hapR* background for repression of cholera toxin (CTX). To this end, secreted protein fractions were prepared in independent biological triplicates as previously described (Herzog *et al*, 2019). Specifically, the strains were cultured in AKI medium (0.5% sodium chloride, 0.4% yeast extract, and 1.5% bacto‐peptone) supplemented with 0.3% sodium bicarbonate to stimulate the production of CTX. Growth under AKI conditions involves biphasic cultures. In the first phase, the cultures were grown in a still tube for 4 h at 37°C. Subsequently, in the second phase, the cultures were poured into a flask to continue growing with shaking. 2 ml of sample was harvested after 16 h of continuous shaking followed by trichloroacetic acid (TCA) preparation of proteins. Pellets were resuspended in volumes of 1× Laemmli buffer relative to the OD_600_ measurements of the respective culture. The same amount of each sample was analyzed simultaneously on two SDS–PAGEs: the first set of gels served as a loading control and were stained using Coomassie brilliant blue stain (0.1% Coomassie R‐250 in 40% ethanol, 10% acetic acid). Additionally, the second set of gels were subjected to immunoblotting using rabbit polyclonal anti‐cholera toxin antibody (#ab123129, Abcam, Cambridge, UK). The corresponding secondary antibody used was goat anti‐rabbit HRP‐conjugated IgG antibody, (#16104, Thermo Fisher Scientific, Waltham, Massachusetts). Signals were visualized using Fusion FX EDGE imager, and the band intensities from both sets of gels were quantified using the BIO‐1D software (Vilber Lourmat, Marne‐la‐Vallée, France). Each secreted fraction from the immunoblot was normalized to its corresponding loading control from the Coomassie‐stained gel. CTX levels were calculated relative to an empty control plasmid (pCMW).

### Electrophoretic mobility shift assays (EMSAs)

EMSAs were carried out as previously described (Manneh‐Roussel *et al*, 2018). Briefly, DNA fragments for EMSA experiments were generated by PCR using KPO‐IGRF and KPO‐IGRR, using genomic DNA from *V. cholerae* strain N16961 as a template. The resulting PCR products were purified and end‐labeled with (γ‐^32^P)‐ATP using T4 polynucleotide kinase (New England Biolabs, Ipswich, Massachusetts). The resulting radiolabeled fragments were incubated with different concentrations of CRP in buffer containing 40 mM Tris acetate (pH 7.9), 1 mM MgCl_2_, 100 mM KCl, and 0.2 mM cAMP. Herring sperm DNA was added as a non‐specific competitor at a final concentration of 12.5 µg/ml. Reactions were incubated at 37°C for 20 min, before being loaded onto a 7.5% non‐denaturing polyacrylamide gel run in 0.5× TBE buffer.

### RNA‐seq analysis: identification of VcdRP targets

*Vibrio cholerae* C6706 Δ*vcdRP* strains harboring pBAD1K‐ctrl, pBAD1K‐*vcdRP*, pBAD1K‐*vcdR*, or pBAD1K‐*vcdP* were cultivated to early exponential phase (OD_600_ of 0.1) and treated with L‐arabinose (0.2% f.c.). After 15 min of treatment, 4.0 OD_600_ units of cells were harvested with 0.2 volumes of “stop‐mix” (95% ethanol, 5% phenol) and snap‐frozen in liquid nitrogen until further processing. Total RNA was isolated with EXTRAzol according to standard protocols and subsequently DNase digested with TURBO DNase (Thermo Fisher Scientific, Waltham, Massachusetts). Depletion of ribosomal RNA was performed using the Ribo‐Zero rRNA removal kit for Gram‐negative bacteria (#MRZGN126, Illumina, San Diego, California). Integrity of the prepared RNA was tested using an Agilent 2100 Bioanalyzer. cDNA libraries were prepared using the NEBNext Ultra II Directional RNA Library Prep Kit for Illumina (#E7760, New England Biolabs, Ipswich, Massachusetts), according to the manufacturer's instructions. The libraries were then sequenced using a HiSeq 1500 System in single‐read mode for 100 cycles. The read files in FASTQ format were imported into CLC Genomics Workbench v12.0.3 (Qiagen, Hilden, Germany) and trimmed for quality and 3’ adaptors. Reads were mapped to the *V. cholerae* reference genome (NCBI accession numbers: NC_002505.1 and NC_002506.1) including annotations for the sRNAs Vcr001‐Vcr230 (Papenfort *et al*, 2015; Huber *et al*, 2020) using the “RNA‐Seq Analysis” tool with standard parameters. Reads mapping in CDS were counted, and genes with a total count cutoff > 10 in all samples were considered for analysis. Read counts were normalized (CPM) and transformed (log2). Differential expression was tested using the built‐in tool corresponding to edgeR in exact mode with tag‐wise dispersions (empirical analysis of DGE). Genes with an absolute fold‐change ≥ 2.0 and an FDR‐adjusted *P*‐value ≤ 0.05 were considered as differentially expressed. Enrichment of GO (gene ontology) terms was analyzed using the DAVID tool v6.8, (Huang *et al*, 2009a, 2009b). The sequencing data have been deposited at NCBI’s Gene Expression Omnibus (Edgar *et al*, 2002) accessible through the GEO series accession number GSE168736.

### VcdP co‐immunoprecipitation

VcdP co‐immunoprecipitations were performed as previously described for tagged *V. cholerae* cells (Huber *et al*, 2020). Briefly, *V. cholerae* wild‐type cells carrying either an empty vector control (pCtrl) or a SPA‐tagged over‐expression plasmid of *vcdP* were grown in LB medium to mid‐log phase. The SPA epitope contains the 3×FLAG and the calmodulin binding peptide sequences separated by a TEV protease cleavage site (Zeghouf *et al*, 2004). Hence, the lysates corresponding to 50 OD_600_ units were subjected to immunoprecipitation using monoclonal anti‐Flag antibody (#F1804; Sigma‐Aldrich, St. Louis, Missouri) and Protein G Sepharose (#P3296; Sigma‐Aldrich). Protein samples were analyzed on immunoblots using anti‐Flag antibodies. Subsequently, these samples were processed using the single‐pot solid‐phase‐enhanced sample preparation (SP3) protocol (Hughes *et al*, 2019) to identify potential protein partners binding to VcdP. The results from the LC‐MS analysis were filtered using the following criteria: protein‐level FDR < 5%, at least two high confidence peptides (FDR < 1%) of which at least one is unique and peptide assigned as high confidence with an FDR ≤ 1%. To validate the findings from the LC‐MS data, cells expressing chromosomally tagged *gltA*::HA or *gltA*::6xHis either an empty vector control (pCtrl) or a SPA‐tagged over‐expression plasmid of *vcdP* were subjected to reciprocal co‐immunoprecipitations using monoclonal anti‐Flag antibody and monoclonal anti‐HA (#ab18181, Abcam, Cambridge, UK) or monoclonal anti‐6xHis (#ab200537, Abcam, Cambridge, UK) antibodies, respectively.

### Mass spectrometry sample preparation and analysis for the identification of proteins binding to VcdP

*Vibrio cholerae* wild‐type cells carrying either an empty vector control (pCtrl) or a SPA‐tagged over‐expression plasmid of *vcdP* were grown in LB medium to mid‐log phase and subjected to co‐immunoprecipitation (IP) as described above. The IP samples were processed using the single‐pot solid‐phase‐enhanced sample preparation (SP3) protocol (Hughes *et al*, 2019). Briefly, the samples were reduced with Tris (2‐carboxyethyl) phosphine, alkylated with chloroacetamide and subsequently precipitated onto magnetic beads (SpeedBeads™ magnetic carboxylate) using ethanol. Following two wash steps, the bead‐associated precipitated proteins were digested in solution with trypsin. The peptides were separated by chromatography on a Dionex U3000 nanoHPLC system (Thermo, Dreieich, Germany) equipped with an Acclaim PepMap 100 C18 column (2 µm, 75 µm × 500 mm) coupled to a QExactive Plus Orbitrap MS (Thermo, Bremen, Germany). The raw MS data were processed using the Proteome Discoverer software package (v2.4.0.305, Thermo, Germany). The files were searched individually using SequestHT algorithm node against a protein database containing genome‐derived proteins of *V. cholerae* serotype O1 (strain ATCC 39315 / El Tor Inaba N16961‐ access:2018.06.26), with VcdP::SPA and mouse IgG appended along with the cRAP list of common laboratory contaminants. Searches were performed with semi‐trypsin specificity with a maximum of four missed cleavages. The results were filtered using the following criteria: protein level FDR < 5%, at least two high confidence peptides (FDR < 1%) of which at least one is unique and peptide assigned as high confidence with an FDR ≤ 1%. The proteins that were identified are shown in Appendix Table S2. All LC‐MS data was deposited to the ProteomeXchange Consortium (Vizcaíno *et al*, 2014) via the PRIDE partner repository (Perez‐Riverol *et al*, 2019) with the dataset identifier PXD025275.

### Purification of citrate synthase variants

*Vibrio cholerae* citrate synthase GltA and its variant that is no longer inhibited by NADH, GltA F383A, were expressed with a N‐terminal 6xHis in the pET15b vector and transformed into BL21 (DE3) *E. coli* cells (Novagen). The proteins were purified as previously described for tagged VqmR protein (Papenfort *et al*, 2017). Briefly, 6xHis::GltA (or GltA F383A) expressing cells were grown at 37°C with shaking to mid‐log phase. IPTG (f.c. of 1 mM) was added and the cultures were grown for an additional 2.5 h. Pellets were collected and re‐suspended in lysis buffer (20 mM imidazole pH 8.0, 450 mM potassium acetate, 50 mM HEPES pH 7.4 and 10 mM BME in PBS supplemented with 1x cOmplete EDTA‐free protease inhibitor cocktail (#4693, Sigma Aldrich, St. Louis, Missouri)). The cells were lysed via sonication (1 min 45 s, amplitude 20%, 5 s pulse, 15 s pause). The cleared lysates were applied to Ni‐NTA resin (#88221, Thermo Fisher Scientific, Waltham, Massachusetts) and rotated end‐to‐end for 2 h at 4°C, following which the resin‐lysate mixture was washed two times with wash buffer (40 mM imidazole pH 8.0, 450 mM potassium acetate, 50 mM HEPES pH 7.4, 5% (v/v) glycerol and 5 mM BME). The washed mixture was then re‐suspended in 10 ml lysis buffer (per ml beads) and loaded on a polypropylene column. After washing with 4x bed volume of resin, on‐column cleavage was induced using elution buffer (250 mM imidazole pH 8.0, 50 mM potassium chloride, 50 mM HEPES pH 7.4 and 5 mM BME). Protein purification was verified by SDS‐PAGE analysis and the concentrations were measured using BCA assay (#23225, Pierce™, ThermoScientific, Waltham, Massachusetts).

### Citrate synthase activity assay

Cell extracts were obtained from *V. cholerae* wild‐type, Δ*vcdRP*, Δ*gltA*, and F383A *gltA* cells carrying either an empty control vector, the VcdP plasmid, or VcdP* plasmid. The cells were grown in LB medium to late stationary phase, and cell pellets were lysed using bead ruptor in a buffer containing 1 M Tris and 0.5 mM EDTA (TE) at pH 8.0. The resulting cell lysates were used to measure the citrate synthase activity in accordance with the protocols described earlier (Duckworth & Tong, 1976; Anderson & Duckworth, 1988; Pereira *et al*, 1994). Specifically, protein concentrations were determined using Pierce™ BCA protein assay kit (#23225, Thermo Fisher Scientific, Waltham, Massachusetts). 1–50 µl of the lysate was used as input for the assay using a Spark 10 M plate reader (Tecan, Männedorf, Switzerland). Equimolar concentration of the substrates acetyl coenzyme A and oxaloacetate were added (f.c. 0.1 mM each). The coupled enzymatic reaction led to the formation of citrate, with the release of thiols. These thiol groups were detected using 0.1 mM of Ellman’s reagent (5,5′‐dithiobis‐(2‐nitrobenzoic acid) or DTNB). The resulting colorimetric product at 412 nm was proportional to the enzymatic activity of citrate synthase present. For *in vitro* citrate synthase activity measurements, a pre‐determined concentration of purified GltA and GltA F383A protein variants were mixed with a specified amount of synthetic VcdP or VcdP* peptides (calculated per monomer of the protein) and used as input for the assay. The enzyme kinetics were assayed at 15‐s intervals for at least 30 min and the activity was determined from the initial velocities. The increase in product was exponential over a short period of time (in the order of 2–3 min) before saturation. The change in absorbance per minute was determined within the linear range. The citrate synthase activity was calculated as change in absorbance per minute per milligram of protein input using the extinction coefficient value of 13.6/mM for TNB at 412 nm.

### Metabolite measurements using mass spectrometry

Glycolysis and citric acid cycle metabolites were measured for *V. cholerae* WT, Δ*vcdRP*, *and* Δ*gltA* each harboring an empty control plasmid (pCtrl) and Δ*vcdRP* with pVcdRP expression plasmids (pVcdRP and pVcdP) as well as Δ*gltA* harboring pVcdP. A modified LC/MS method as described previously was used for the measurement and analysis (Buescher *et al*, 2010). Briefly, cells were grown in LB medium to exponential and stationary phase and were subsequently quenched in ice‐cold methanol. The quenched samples were centrifuged at 3000× *g* for 10 min at −9°C. The frozen cell pellets were thawed on ice and resuspended in 500 µl methanol. After three freeze (liquid nitrogen) and thaw cycles, the sample was centrifuged (16,000×* g*, −4°C and 5 min). The supernatant was transferred into a new tube and stored on ice. The extraction step was repeated, and the supernatants were combined prior to evaporation (Concentrator plus, Eppendorf, Hamburg, Germany). The mass spectral data were acquired on a QTRAP 6500+® system (Sciex, Framingham, MA, USA) coupled on‐line with an Agilent 1290 II infinity UPLC system (Agilent Technologies Inc., Santa Clara, CA, USA). Each sample was resuspended in 100 µl Milli‐Q water, and 10 µl was injected onto a XSelect HSS T3 XP column (2.1 × 150 mm, 2.5 µm, 100 Å; Waters, Milford, MA, USA). Metabolites were eluted at a flow rate ranging from 0.4 ml/min to 0.15 ml/min with a non‐linear gradient. Mobile phase A and mobile phase B were 10 mM tributylamine, 10 mM acetic acid, 5% methanol and 2% 2‐propanol (pH 7.1) in water and 100% 2‐propanol, respectively. The autosampler was kept at 5°C and the temperature of the column oven was set to 40°C. Identification and relative quantification were based on specific MRM transitions measured in negative mode electrospray ionization. Data acquisition and analysis were performed with the Analyst® software (v1.7.1). The metabolome data are available at the NIH Common Fund's National Metabolomics Data Repository (NMDR) website, the Metabolomics Workbench, where it has been assigned the study ID ST001752. The data can be accessed directly via its project https://doi.org/10.21228/M8JD7N.

### Quantitative real‐time PCR

Total RNA was harvested from *V. cholerae* wild‐type and Δ*vcdRP* strains harboring an empty control vector (pCtrl) or Δ*vcdRP* cells harboring a VcdRP overexpression plasmid (pVcdRP). Cultures were grown in M9 medium supplemented with 0.4% glucose and 0.4% casaminoacids. Additionally, *V. cholerae* wild‐type and Δ*cyaA* strains were grown without (‐) or with (+) cAMP (f.c. 5 mM) in AKI medium to stimulate production of CTX. Growth under AKI conditions involve biphasic cultures. In the first phase, the cultures were grown in a still tube for 4 h at 37°C. Subsequently, in the second phase, the cultures were poured into a flask to continue growing with shaking. At the appropriate time‐point, RNA equivalent to 2.0 OD_600_ units of cells was extracted using the SV Total RNA Isolation System (Promega, Fitchburg, Wisconsin) according to manufacturer’s instructions. The qRT‐PCR was performed on independent biological triplicates using Luna Universal One‐Step RT qPCR kit (New England Biolabs, Ipswich, Massachusetts) in a MyiQ™ Single‐Color Real‐Time PCR Detection System (Bio‐Rad, Hercules, California). *recA* served as the reference house‐keeping gene and the oligonucleotides used for all qRT‐PCR analyses are provided in Appendix Table S5.

### Sequence alignment and gene synteny analysis

The *vcdRP* gene and its promoter sequences among various *Vibrio* species were aligned using the MultAlin webtool (Corpet, 1988). Vch: *Vibrio cholerae* (NCBI: txid243277), Vfu: *Vibrio furnissii* (NCBI: txid29494), Vha: *Vibrio harveyi* (NCBI: txid33843), Vpa: *Vibrio parahaemolyticus* (NCBI: txid670), Vvu: *Vibrio vulnificus* (NCBI: txid672), Van: *Vibrio anguillarum* (NCBI: txid55601) and Vsp: *Vibrio splendidus* (NCBI: txid575788). Gene synteny analysis of the genomic loci encoding *vcdRP* in various *Vibrio* strains was performed using SynTax (Oberto, 2013).

### Quantification and statistical analyses

The statistical parameters including the number of independent biological replicates are indicated in the legend corresponding to each figure. Normality test was performed using Shapiro–Wilk whereas equal variance was determined using Brown–Forsythe test. The data were tested for significant differences using ANOVA and post hoc Tukey or Dunnett’s tests, where appropriate, and the *P*‐values have been indicated against each figure. Statistical analysis was performed using Prism 9.0.0 (GraphPad Software Inc., San Diego, California). No blinding or randomization was used in the experiments. Also, no estimation of power analysis was calculated before performing the experiments. Northern and Western blots were visualized using Fiji (Schindelin *et al*, 2012), and the latter were quantified using the BIO‐1D software (Vilber Lourmat, Marne‐la‐Vallée, France).

## Author contributions

KV, MH, URK, AT, MvB, DCG, and KP designed the experiments; KV, MH, JRJH, LC, and BE performed the experiments; KV, MH, JRJH, LC, URK, BE, and KP analyzed data; and KP wrote the manuscript.

## Conflict of interest

The authors declare that they have no conflict of interest.

## Supporting information



Appendix

Expanded View Figures PDF

Source Data for Expanded View and Appendix

Source Data for Figure 1

Source Data for Figure 2

Source Data for Figure 3

Source Data for Figure 4

Source Data for Figure 5

Source Data for Figure 6

## Data Availability

The following datasets were generated in this study:
The transcriptome data for VcdRP target identification is accessible at the NCBI Gene Expression Omninus through the GEO series accession number GSE168736. https://www.ncbi.nlm.nih.gov/geo/query/acc.cgi?acc=GSE144478
The LC‐MS data for identification of VcdP interacting partners have been submitted to the ProteomeXchange Consortium via the PRoteomics IDEntifications (PRIDE) partner repository with the dataset identifier PXD025275. http://proteomecentral.proteomexchange.org/cgi/GetDataset?ID=PXD025275
The metabolome data are available at the NIH Common Fund's National Metabolomics Data Repository (NMDR) website, the Metabolomics Workbench, where it has been assigned the study ID ST001752. The data can be accessed directly via its project https://doi.org/10.21228/M8JD7N. https://www.metabolomicsworkbench.org/data/DRCCMetadata.php?Mode=Project&ProjectID=PR001123
