# Supplementary material for: A dual‐function RNA balances carbon uptake and central metabolism in Vibrio cholerae
Source: EMBO J. 2021 Oct 6;40(24):e108542. doi: 10.15252/embj.2021108542 (PMC8672173; doi:10.15252/embj.2021108542)
Supplement: Supplementary file 1 — Appendix [file EMBJ-40-e108542-s007.pdf]

A dual-function RNA balances carbon uptake and central metabolism in *Vibrio cholerae*

Kavyaa Venkat[1*], Mona Hoyos[1*], James R. J. Haycocks[2], Liam Cassidy[3], Beatrice Engelmann[4], Ulrike Rolle-Kampczyk[4], Martin von Bergen[4], Andreas Tholey[3], David C. Grainger[2], and Kai Papenfort[1,5#]

1 Friedrich Schiller University, Institute of Microbiology, Jena, Germany

2 Institute of Microbiology and Infection, University of Birmingham, United Kingdom

3 Systematic Proteome Research & Bioanalytics, University of Kiel, Germany

4 Helmholtz Centre for Environmental Research-UFZ, Leipzig, Germany

5 Microverse Cluster, Friedrich Schiller University Jena, Jena, Germany

* these authors contributed equally

# Corresponding author: kai.papenfort@uni-jena.de

This supplement contains:

Appendix Figures S1-S5

Appendix Tables S1-S5

Appendix supplementary references

APPENDIX

APPENDIX FIGURES

Appendix Fig. S1 *vcdRP* harbors a consensus motif for CRP-dependent promoters………………………………………………………… 3

Appendix Fig. S2 Target spectrum of VcdR……………………………………….. 4

Appendix Fig. S3 Mutated variant of VcdP………………………………………… 5

Appendix Fig. S4 Metabolite profile of glycolysis and TCA cycle intermediates upon deletion of citrate synthase………………………………… 6

Appendix Fig. S5 Citrate synthase activity measurements for *V. natriegens* and *B. subtilis*…………………………………………………………… 8

APPENDIX TABLES

Appendix Table S1 Differentially expressed genes regulated by *vcdRP* and *vcdR/P* pulse expression…………………………………….. 9

Appendix Table S2 Proteins identified via LC-MS as potential interaction partners of VcdP::SPA……………………………………….. 12

Appendix Table S3 Bacterial strains used in this study……………………………. 14

Appendix Table S4 Plasmids used in this study……………………………………. 14

Appendix Table S5 DNA oligonucleotides used in this study……………………… 17

Appendix supplementary references………………………………………………. 22

Appendix Fig. S1

A

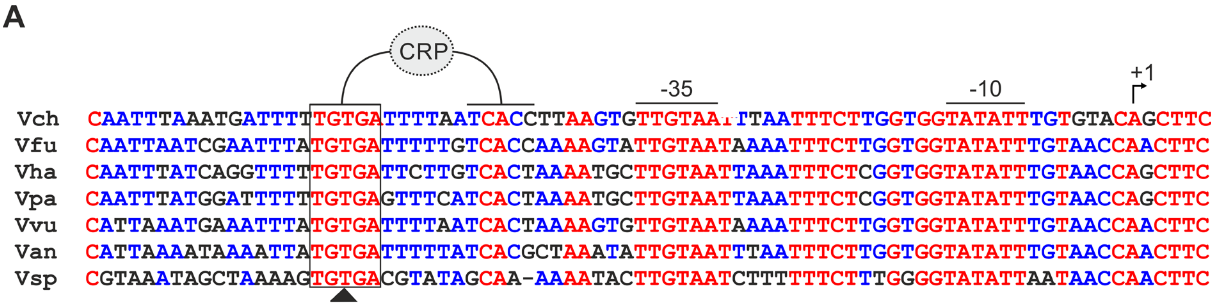

B

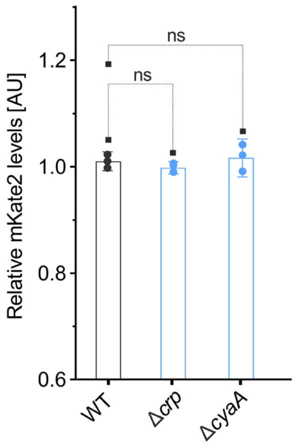

Appendix Figure S1. *vcdRP* harbors a consensus motif for CRP-dependent promoters

A. Alignment of promoter regions of *vcdRP* from various *Vibrio* species: *V. cholerae* (Vch), *V. furnissi* (Vfu), *V. harveyi* (Vha), *V. parahaemolyticus* (Vpa), *V. vulnificus* (Vvu), *V. anguillarum* (Van) and *V. splendidus* (Vsp). The CRP-binding site (grey), -35 box, -10 box and TSS (arrow) are indicated. The black box and the solid triangle represents the site that was deleted for the reporter fusions measured in (B).

B. Relative fluorescence intensities of *V. cholerae* wild-type or mutants of *crp* and *cyaA* harboring a transcriptional reporter plasmid with a part of the CRP box deleted (solid triangle in A) in the promoter of *vcdRP* fused to *mKate2*. Cells were grown in LB to OD_{600} of 1.0 and fluorophore production was measured. The fluorescence of WT was set to 1.

Data information: Data in (B) are presented as mean ± SD, *n* = 3 independent biological replicates. Statistical significance was determined using one-way ANOVA and post hoc Tukey's multiple comparisons test. The p-value is summarized as follows - ns for p > 0.05. Source data are available online for this figure.

Appendix Fig. S2

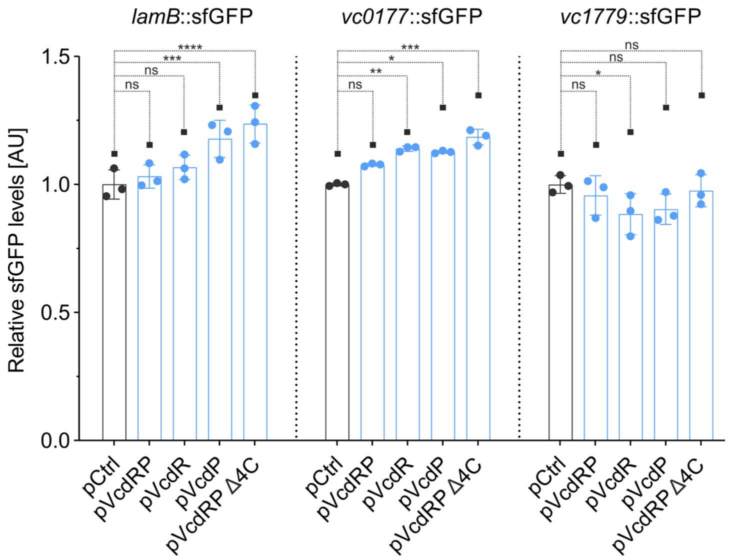

Appendix Figure S2. Target spectrum of VcdR

Relative fluorescence intensities (*y*-axis) of *E. coli* strains harboring the gene-specific translational reporters for *lamB*, *vc0177* and *vc01779* fused to *sfGFP*, combined with either an empty vector control (pCtrl) or *vcdRP* expression plasmids (pVcdRP, pVcdR, pVcdP and pVcdRP Δ4C on *x*-axis). Cells were grown in LB to OD_{600} of 1.0 and fluorophore production was measured. The fluorescence of pCtrl (for each reporter fusion) was set to 1.

Data information: Data in (A) are presented as mean ± SD, *n* = 3 independent biological replicates. Statistical significance was determined using one-way or two-way ANOVA and post hoc Dunnett's multiple comparisons test. The p-value is summarized as follows - ns for p > 0.05, * for p ≤ 0.05, ** for p ≤ 0.01, *** for p ≤ 0.001 and **** for p ≤ 0.0001. Source data are available online for this figure.

Appendix Fig. S3

```
        1              10                  20                29
VcdP    MNKGLSSAMFWNQHSVYTGNFAYPRSFGY
VcdP*   MNKGLSSAMFWNQHAAAAGNFAYPRSFGY
```

Appendix Figure S3. Mutated variant of VcdP

A mutated version of VcdP was generated by replacing the amino acids from positions 15-18 of the small protein with alanine as underlined in black.

Appendix Fig. S4

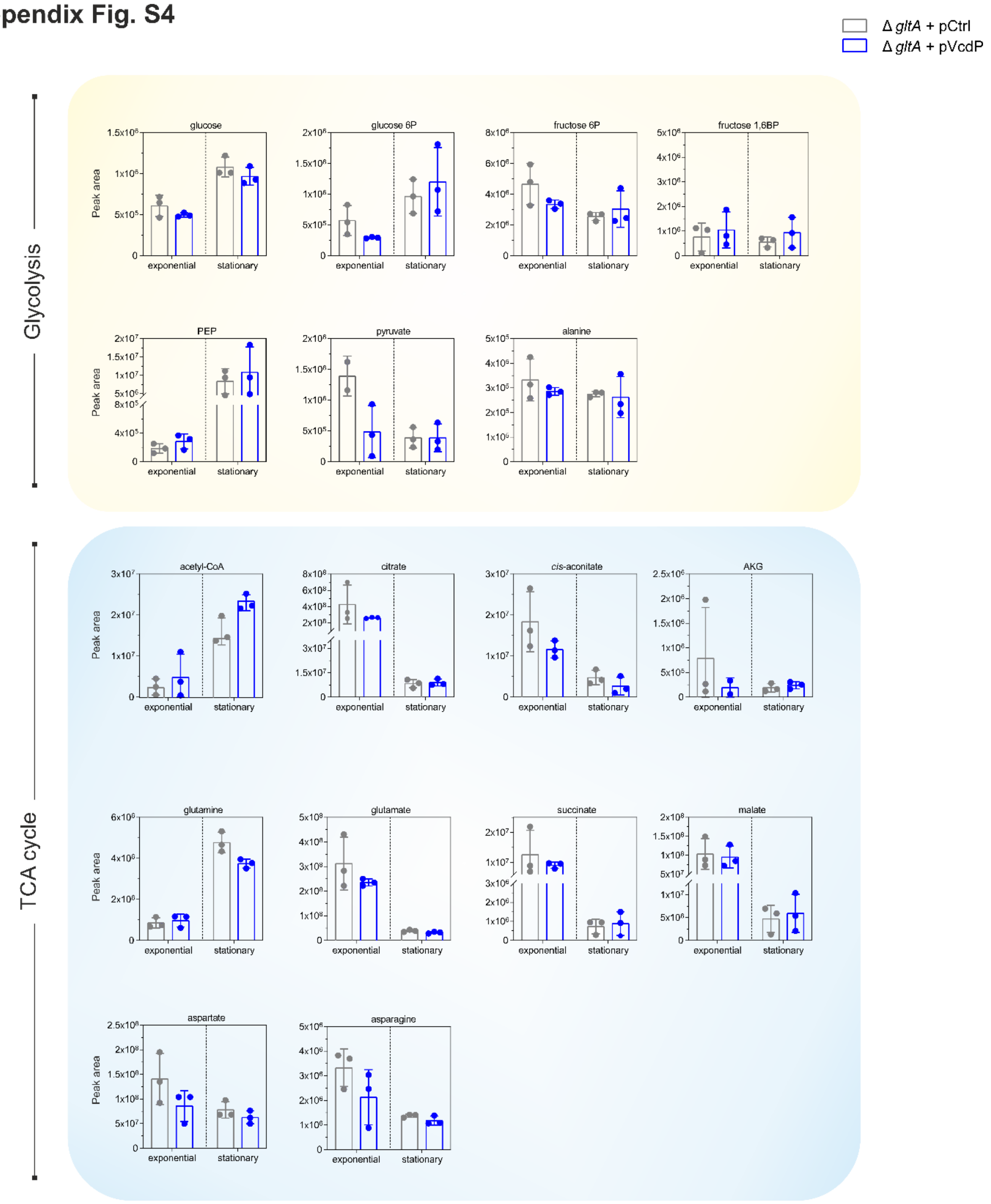

Appendix Figure S4. Metabolite profile of glycolysis and citric acid cycle intermediates upon deletion of citrate synthase.

Metabolite abundance of glycolytic and citric acid cycle intermediates in exponential (OD_{600} of 0.5) and stationary phase (OD_{600} of 3.0) of growth were measured for *V. cholerae* Δ*gltA*, harboring either an empty vector control (pCtrl) or *vcdP* expression plasmid (pVcdP). The *y-axis* represents the peak area of each metabolite determined by mass spectrometry. The main operative pathways are color coded (yellow for glycolysis and blue for citric acid cycle).

Data information: data are presented as mean ± SEM, *n* = 3 independent biological replicates. Abbreviations: glucose-6P (glucose 6-phosphate), fructose-6P (fructose 6-phosphate), fructose-1,6BP (fructose 1,6-bisphosphate), PEP (phosphoenolpyruvate). Source data are available online for this figure.

Appendix Fig. S5

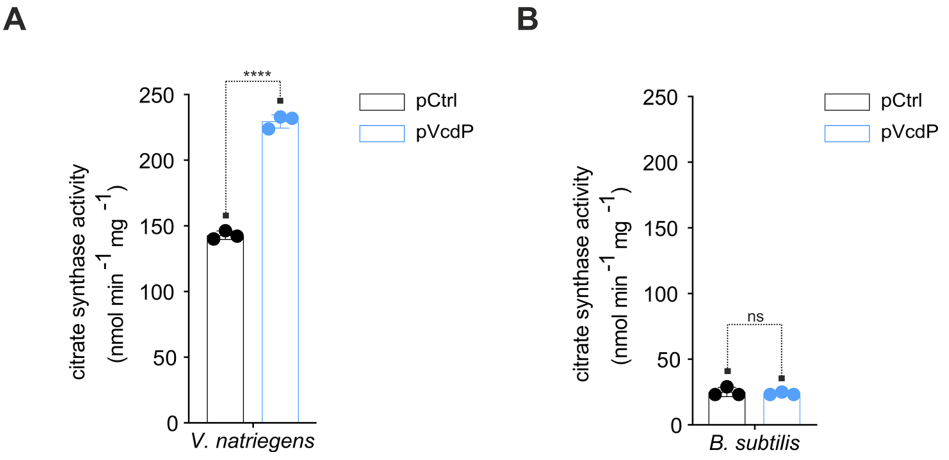

Appendix Fig. S5. Citrate synthase activity measurements for *V. natriegens* and *B. subtilis*

A-B: An empty vector control (pCtrl) or *vcdP* expression plasmids (pVcdP) were conjugated into *V. natriengens* (A) and *B. subtilis* (B) cells. Cells were grown in appropriate rich medium (LB for *B. subtilis* or LB supplemented with 5% sodium chloride for *V. natriengens*) and subsequently lysed. These cellular extracts served as input for measuring the activity of citrate synthase enzyme colorimetrically at an absorbance of 412nm.

Data information: data in (A) and (B) are presented as mean ± SD, $n = 3$ independent biological replicates. Statistical significance was determined using one-way ANOVA and post hoc Tukey's multiple comparisons test. The p-value is summarized as follows - ns for $p > 0.05$ and **** for $p \leq 0.0001$. Source data are available online for this figure.

Appendix Table S1: Differentially expressed genes regulated by *vcdRP* and *vcdR/P* pulse expression

#Description is based on the annotation at KEGG (https://www.genome.jp/kegg).

§Genes with a total count cutoff >10 in all samples, with an absolute fold-change ≥ 2.0 and a FDR adjusted p-value ≤ 0.05 were considered to be differentially regulated.

The color codes are based on the Venn diagram in Fig. 3C

Gene	Description#	Fold change§		
		pVcdRP vs. pCtrl	pVcdR vs. pCtrl	pVcdP vs. pCtrl
astD	Succinylglutamic semialdehyde dehydrogenase	2.3	1.1	1.7
carB	Carbamoyl-phosphate synthase large subunit	1.4	1.0	2.0
cpdB	2',3'-cyclic-nucleotide 2'-phosphodiesterase / 3'-nucleotidase	2.2	1.3	1.4
galM	Aldose 1-epimerase	2.1	1.4	1.4
glpK	Glycerol kinase	4.0	1.5	2.5
glpQ	Glycerophosphoryl diester phosphodiesterase	3.3	1.7	1.4
glpT	Glycerol-3-phosphate transporter	3.6	1.5	1.2
glpX	Fructose-1,6-bisphosphatase II	1.3	1.1	2.1
gltA	Citrate synthase	2.1	1.4	1.4
lamB	Maltoprotein	10.0	2.0	4.4
lldD	L-lactate dehydrogenase	2.1	1.2	1.5
malE	Maleylacetate reductase	7.4	1.8	3.4
malF	Maltose/maltodextrin transport system permease protein	4.0	1.6	2.7
malG	Maltose/maltodextrin transport system permease protein	4.0	1.6	2.7
malQ	4-alpha-glucanotransferase	4.5	1.5	3.1
malS	Alpha-amylase	2.6	1.2	2.2
mglC	Methyl-galactoside transport system permease protein	4.2	1.5	2.1
nagE	PTS system N-acetylglucosamine-specific transporter subunit IIABC	-3.1	-3.3	1.1
potE	Putrescine transporter	3.4	1.3	1.6
ptsG	PTS system glucose-specific transporter subunits IIBC	-2.7	-4.9	1.5
ptsH	Phosphocarrier protein HPr	-4.0	-3.5	1.3
ptsI	Phosphoenolpyruvate-protein phosphotransferase	-2.5	-3.4	1.4
purN	Phosphoribosylglycinamide formyltransferase 1	1.2	1.1	2.1
purT	Phosphoribosylglycinamide formyltransferase 2	1.5	1.0	2.6
pyrB	Aspartate carbamoyltransferase	-1.0	-1.1	2.3
rbsC	Ribose transport system permease protein	6.8	1.4	2.8
sdhA	Succinate dehydrogenase, flavoprotein subunit	2.1	1.3	1.6
sdhC	Succinate dehydrogenase / fumarate reductase	2.1	1.3	1.2
sucA	2-oxoglutarate dehydrogenase E1 component	2.1	1.3	1.5
sucC	Succinyl coa synthetase, beta subunit	3.3	1.5	1.9
tnaC leader peptide	Tryptophanase leader peptide	3.0	1.7	1.5
treB	Trehalose PTS system EIIBC	-1.5	-2.4	1.6
ushA	5'-nucleotidase / UDP-sugar diphosphatase	2.0	1.4	1.4

vc0177	Hypothetical protein	-2.0	-2.1	1.3
vc0216	Methyl-accepting chemotaxis protein	2.3	1.3	1.3
vc0282	Methyl-accepting chemotaxis protein	2.1	1.4	1.3
vc0338	Solute carrier family 13 (sodium-dependent dicarboxylate transporter)	2.0	1.2	1.3
vc0384	Sulfite reductase (NADPH) flavoprotein alpha-component	1.7	1.1	2.0
vc0432	Malate dehydrogenase	2.3	1.4	1.8
vc0706	Sigma-54 modulation protein	2.3	1.5	1.1
vc0931	Hypothetical protein	-1.1	1.4	2.0
vc1325	Galactoside ABC transporter periplasmic D-galactose/D-glucose-binding protein	6.1	1.8	3.1
vc1327	Galactose/methyl galaxtoside transporter ATP-binding protein	4.3	1.5	2.4
vc1446	ATP-binding cassette, subfamily B, bacterial RtxE	-2.4	-1.4	-1.7
vc1447	Membrane fusion protein, RTX toxin transport system	-2.7	-1.3	-1.5
vc1448	ATP-binding cassette, subfamily B, bacterial RtxB	-3.3	-1.5	--1.9
vc1449	Hypothetical protein	-2.2	-1.8	-1.4
vc1450	RTX toxin activating protein	-2.0	-1.6	-1.3
vc1492	Glutamate dehydrogenase	2.7	1.4	1.7
vc1539a	Hypothetical protein	2.2	1.3	1.4
vc1595	Galactokinase	2.2	1.4	1.6
vc1596	Galactose-1-phosphate uridylyltransferase	2.1	1.3	1.6
vc1658	Serine transporter	-2.0	-1.2	-1.5
vc1696	DNA-binding protein	2.1	1.3	1.2
vc1741	Tetr family transcriptional regulator	2.0	1.5	1.3
vc1774	N-acetylneuraminic acid mutarotase	2.1	1.1	1.4
vc1776	N-acetylneuraminate lyase	4.1	1.2	1.6
vc1777	Hypothetical protein	4.7	1.2	1.9
vc1778	Hypothetical protein	6.9	1.4	2.2
vc1779	C4-dicarboxylate-binding protein	9.9	2.1	3.7
vc1781	N-acetylmannosamine-6-phosphate 2-epimerase	6.8	1.4	3.1
vc1782	N-acetylmannosamine kinase	2.7	1.2	2.0
vc1783	N-acetylglucosamine-6-phosphate deacetylase	2.2	1.0	1.5
vc1784	Neuraminidase	2.7	1.2	1.9
vc1822	PTS system fructose-specific transporter subunit IIABC	2.5	1.5	1.4
vc1823	PTS system fructose-specific transporter subunit IIB	2.3	1.4	1.4
vc1824	PTS system nitrogen regulatory subunit IIA	2.5	1.5	2.0
vc1898	Methyl-accepting chemotaxis protein	2.8	1.4	1.5
vc1905	Alanine dehydrogenase	2.4	1.4	2.1
vc1953	Concentrative nucleoside transporter, CNT family	-4.8	-1.5	-4.5
vc1998	Methionine sulfoxide reductase B	-2.3	-1.9	-1.2
vc2084	Succinyl-coa synthetase subunit alpha	3.3	1.6	2.0
vc2086	Dihydrolipoamide succinyltransferase	2.2	1.3	1.7
vc2277	Xanthine-guanine phosphoribosyltransferase	-2.2	-1.9	-1.1
vc2305	Outer membrane protein OmpK	2.8	1.3	1.6
vc2338	Pseudogene	2.1	1.3	1.6
vc2350	2-deoxyribose-5-phosphate aldolase	-1.3	1.2	-2.2

vc2352	Concentrative nucleoside transporter, CNT family	2.1	1.5	1.3
vc2416	2`,3`-cyclic-nucleotide 2`-phosphodiesterase	2.1	1.3	1.4
vc2511	Aspartate carbamoyltransferase	-1.0	-1.1	2.0
vc2544	Fructose-1,6-bisphosphatase	2.2	1.3	1.9
vc2600	Hypothetical protein	-2.0	-1.3	-1.4
vc2656	Fumarate reductase flavoprotein subunit	2.5	1.4	2.2
vc2657	Fumarate reductase iron-sulfur subunit	2.4	1.3	2.3
vc2658	Fumarate reductase subunit C	2.2	1.3	2.1
vc2659	Fumarate reductase subunit D	2.6	1.3	2.1
vc2667	Hypothetical protein	1.1	1.5	2.4
vc2738	Phosphoenolpyruvate carboxykinase	2.2	1.1	1.8
vc2761	Inner membrane transport protein YdhC	-3.6	1.2	-5.7
vca0013	Maltodextrin phosphorylase	3.5	1.4	2.5
vca0015	Pseudogene	2.7	1.5	2.1
vca0025	Nadc family protein	3.6	1.6	1.9
vca0037	Periplasmic copper chaperone A	2.0	1.3	1.2
vca0052	Hypothetical protein	-1.4	1.3	-4.1
vca0053	Purine nucleoside phosphorylase	--1.9	1.4	-7.0
vca0087	Hypothetical protein	-2.1	-1.7	-1.7
vca0127	D-ribose pyranase	9.4	1.6	3.8
vca0128	D-ribose transporter ATP-binding protein	8.9	1.4	3.2
vca0130	D-ribose transporter subunit rbsb	8.9	1.5	2.6
vca0131	Ribokinase	3.9	1.0	1.9
vca0179	Concentrative nucleoside transporter, CNT family	-2.3	-1.3	-1.6
vca0205	Anaerobic C4-dicarboxylate transporter	2.5	1.3	1.5
vca0276	Pseudogene	2.6	1.5	2.4
vca0277	Glycine cleavage system protein H	2.1	1.2	1.5
vca0280	Pseudogene	2.3	1.4	1.5
vca0556	Hypothetical protein	-2.2	-1.2	-1.4
vca0610	Isoprenoid biosynthesis protein	2.4	1.7	1.5
vca0623	Transaldolase B	-1.5	1.3	-2.3
vca0624	Transketolase	-1.4	1.2	-2.4
vca0743	Hypothetical protein	-1.1	1.2	-2.0
vca0745	Pseudogene	4.5	1.5	2.3
vca0843	Glyceraldehyde-3-phosphate dehydrogenase	3.1	1.3	1.9
vca0867	Outer membrane protein W	3.1	1.6	1.3
vca0946	Maltose/maltodextrin transporter ATP-binding protein	2.4	1.2	2.3
vca0985	Oxidoreductase/iron-sulfur cluster-binding protein	2.1	1.3	1.5
vca0987	Phosphoenolpyruvate synthase	4.5	1.3	3.7
vca1027	Maltose operon periplasmic protein	2.6	1.0	1.5
vca1063	Ornithine decarboxylase	5.5	1.7	2.7
vca1069	Methyl-accepting chemotaxis protein	2.5	1.3	1.4

Appendix Table S2: Proteins identified via LC-MS as potential interaction partners of VcdP::SPA.

Only proteins identified co-precipitating with VcdP::SPA in at least two out of three biological replicates, and that were absent from control samples, are listed. A minimum of two high confidence peptides with at least one unique peptide, and a protein level FDR <5%, was required for a protein to be classed as identified.

§Identified in all three biological replicates (all other proteins were identified in two out of three biological replicates).
*The VcdP::SPA protein was given the arbitrary accession number A00055_SPA, to allow for database searches.
(PSM: peptide spectral matches, MW: molecular weight).

Accession	Description	# Unique Peptides	# PSM	MW [kDa]
Q9KQA8§	Citrate synthase	15	55	48.6
A00055_SPA*	VcdP::SPA	11	54	11.3
Q9KTY5	Inositol-1-monophosphatase	6	24	29.1
Q9KV04	Peptidyl-prolyl cis-trans isomerase	7	22	28.1
Q9KR21	PTS system, fructose-specific II ABC component	11	20	65.6
Q9KLJ9	Glycerol kinase	8	19	55.6
P0C6C3	Flagellin A	8	18	40.4
Q9KV30	DNA-directed RNA polymerase subunit beta	15	16	149.4
O34242	Chaperone protein DnaJ	7	15	40.8
P0C6C5	Flagellin C	3	15	39.9
Q9KRZ1	Uncharacterized protein	10	15	183.4
Q9KSW2	ATP-dependent Clp protease, ATP-binding subunit ClpA	12	14	84
Q9KLA3	Glyceraldehyde 3-phosphate dehydrogenase	10	14	55.6
Q9KUT5	Immunogenic protein	7	14	35.2
Q9KQL3	Long-chain-fatty-acid-CoA ligase	8	14	62.8
Q9KQB6	Succinate-CoA ligase [ADP-forming] subunit alpha	10	14	29.9
Q9KUR3	A/G-specific adenine glycosylase	4	12	40.1
Q9KU42	Carbon starvation protein A, putative	4	12	53.1
Q9KKM6	Uncharacterized protein	8	12	67.1
Q9KPH4	Protein translocase subunit SecA	6	11	102.4
Q9KNH4	ATP synthase gamma chain	7	10	31.8
Q9KU11	DNA-binding response regulator PhoB	4	10	26.2
Q9KT50	Nucleoid-associated protein VC_1055	3	10	12
Q9KT11	Oligopeptide ABC transporter, ATP-binding protein	6	9	36.1
Q9KQB4	Dihydrolipoyllysine-residue succinyltransferase component of 2-oxoglutarate dehydrogenase complex	8	8	44.1
Q9KQT0	Peptidylprolyl isomerase	4	8	68.4
Q9KTB7	Adenylate kinase	4	7	23.3
Q9KUT3	Malate dehydrogenase	5	7	32
Q9KR88	Paraquat-inducible protein B	3	7	60.9
Q9KTW0	Uncharacterized protein	3	7	21.1

Q9KUZ2	30S ribosomal protein S6	3	6	14.2
Q9KUR8	Co-chaperone protein DjlA	5	6	31.9
Q9KQU1	Cys regulon transcriptional activator	3	6	36.2
Q9KN38	D-ribose pyranase	2	6	15.3
Q9KSF2	Fumarate hydratase class I	4	6	54.7
Q9KLH1	Methyl-accepting chemotaxis protein	1	6	52.1
Q9KVH6	Peptide ABC transporter, ATP-binding protein	6	6	63.5
Q9KL10	Transcriptional regulator, DeoR family	4	6	28
Q9KTX1	4-hydroxy-3-methylbut-2-en-1-yl diphosphate synthase (flavodoxin)	3	5	40.6
Q9KSD1	Galactose/methyl galactoside import ATP-binding protein MglA	5	5	56.4
Q9KNG6	ParA family protein	5	5	28
Q9KSW1	Putative transport protein VC_1145	3	5	60.5
Q9KPK5	Threonine synthase	4	5	46.2
Q9KNG4	tRNA uridine 5-carboxymethylaminomethyl modification enzyme MnmG	5	5	70.1
Q9KL28	Uncharacterized protein	5	5	42.4
Q9KNC8	Uncharacterized protein	4	5	16.4
Q9KPT0	Uncharacterized protein	4	5	39.8
Q9KUY1	Uncharacterized protein	3	5	16.7
P23247	Aspartate-semialdehyde dehydrogenase 2	2	4	37.4
Q9KUJ8	Beta-ketoadipate enol-lactone hydrolase, putative	3	4	30
Q9KR62	Putative N-acetylmannosamine-6-phosphate 2-epimerase	2	4	25.5
Q9KSF0	Uncharacterized protein	2	4	51.9

Supplementary Table S3: Bacterial strains used in this study.

Strain	Relevant markers/ genotype	Reference/ source
V. cholerae		
KPS-0014	C6706 wild-type	(Thelin & Taylor, 1996)
KPS-0053	C6706 Δ*hapR*	(Svenningsen et al, 2009)
KPVC10141	C6706 *rne*-3071	(Hoyos et al, 2020)
KPVC-10609	C6706 Δ*vcdRP*	This study
KPVC-10985	C6706 Δ*crp*	This study
KPVC-11023	C6706 Δ*cyaA*	This study
KPVC-13609	C6706 *gltA*::*gltA*-HA	This study
KPVC-13611	C6706 *gltA*::*gltA*-6xHis	This study
KPVC-13686	C6706 Δ*gltA*	This study
KPVC-13724	C6706 *gltA F383A*	This study
KPVC-13820	C6706 *vcdP*:: *vcdP*-SPA	This study
V. natriegens		
KPVC-14140	ATCC 14048 wild-type	(Baumann et al, 1980)
B. subtilis		
KPVC-14141	168 wild-type	(Burkholder & Giles, 1947)
E. coli		
TOP10	F- *mcrA* Δ(*mrr-hsdRMS-mcrBC*) φ80*lacZ*ΔM15 Δ*lacX*74 *nupG recA*1 *araD*139 Δ(*ara-leu*)7697 *galE*15 *galK*16 *rpsL*(Str^R) *endA1* λ^-	Invitrogen
S17λpir	Δ*lacU*169 (Φ*lacZ*ΔM15), *recA*1, *endA*1, *hsdR*17, *thi*-1, *gyrA*96, *relA*1, λ*pir*	(Simon et al, 1983)
BL21(DE3)	F^- *ompT* *hsd*S_B (r_B^-, m_B^-) *gal dcm* (DE3)	Novagen
KPEC53467	Top10 Δ*hfq*	(Hoyos et al, 2020)

Supplementary Table S4: Plasmids used in this study.

Plasmid trivial name	Plasmid stock name	Comment	Origin, marker	Reference
pEVS143	pEVS143	Constitutive over-expression plasmid	p15A, Kan^R	(Dunn et al, 2006)
pKAS32	pKAS32	Suicide plasmid for allelic exchange	R6K, Amp^R	(Skorupski & Taylor, 1996)
pXG10-SF	pXG10-SF	Control plasmid	pSC101*, Cm^R	(Corcoran et al, 2012)
pCMW-1	pCMW-1	Control plasmid	p15A, Kan^R	(Waters & Bassler, 2006)
pEVS143-*spot42*	pAS001	*spot42* expression plasmid	p15A, Kan^R	This study
pEVS143-*tarB*	pAS002	*tarB* expression plasmid	p15A, Kan^R	This study
pEVS143-*tarA*	pAS003	*tarA* expression plasmid	p15A, Kan^R	This study
pEVS143-*vqmR*	pKP333	*vqmR* expression plasmid	p15A, Kan^R	(Papenfort et al, 2015)
pEVS143-*vcdP*-SPA	pKV114	*vcdP-SPA* expression plasmid	p15A, Kan^R	This study

pKAS32-*gltA*::3xFlag	pKV150	*gltA*::3xFlag allelic replacement	R6K, Amp^R	This study
pKAS32-*gltA*::HA	pKV152	*gltA*::HA allelic replacement	R6K, Amp^R	This study
pKAS32-*gltA*::6xHis	pKV153	*gltA*::6xHis allelic replacement	R6K, Amp^R	This study
pKAS32-Δ*gltA*	pKV154	Suicide plasmid for *gltA* knockout	R6K, Amp^R	This study
pKAS32-*gltA F383A*	pKV155	Suicide plasmid for *gltA F383A* allelic replacement	R6K, Amp^R	This study
pET15b-*gltA F383A*	pKV156	Over-expression construct for GltA F383A purification	pBR322, Amp^R	This study
pKAS32-*vcdP*::SPA	pKV157	*vcdP*::SPA allelic replacement	R6K, Amp^R	This study
pEVS143-*vcdP**	pKV159	*vcdP** expression plasmid	p15A, Kan^R	This study
pCMW1C-ΔCRP box-pVcdRP ::mKate2	pKV164	Transcriptional reporter for *vcdRP*	p15A, Cm^R	This study
pBSmul2	pKV168	*B. subtilis* expression plasmid	pUB110, Amp^R	(Brockmeier et al, 2006)
pBSmul2-*vcdP*	pKV169	*B. subtilis* expression plasmid	pUB110, Amp^R	This study
pEVS143-*gltA*	pKV175	*gltA* expression plasmid	p15A, Kan^R	This study
pBAD1K-*gltA*	pKV176	*gltA* expression plasmid	p15A, Kan^R	This study
pKAS32-rne3071	pMD003	Suicide plasmid for temperature sensitive *rne* allele	R6K, Amp^R	(Hoyos et al, 2020)
pBAD1K-ctrl	pMD004	Control plasmid	p15A, Kan^R	Lab collection
pKAS32-Δ*vcdRP*	pMD054	Suicide plasmid for *vcdRP* knockout	R6K, Amp^R	This study
pEVS143-*vcdR*	pMD055	*vcdR* expression plasmid	p15A, Kan^R	This study
pEVS143-*vcdRP 87nt*	pMD062	*vcdRP* expression plasmid truncated at 87th nucleotide (from the 3' end)	p15A, Kan^R	This study
pEVS143-*vcdRP 71nt*	pMD063	*vcdRP* expression plasmid truncated at 71st nucleotide (from the 3' end)	p15A, Kan^R	This study
pCMW1C-pVcdRP::mKate2	pMD064	Transcriptional reporter for *vcdRP*	p15A, Cm^R	This study
pEVS143-*vcdP*::3xFlag	pMD065	*vcdP::*3xFlag expression plasmid	p15A, Kan^R	This study
pBAD1K-*vcdRP*	pMD072	*vcdRP* expression plasmid	p15A, Kan^R	This study
pBAD1K-*vcdR*	pMD077	*vcdR* expression plasmid	p15A, Kan^R	This study
pEVS143 (protein)	pMD080	Modified expression plasmid with artificial 5'UTR, MCS and T1 terminator	p15A, Kan^R	This study
pEVS143-*vcdRP* Δ4C	pMD083	*vcdRP* Δ4C expression plasmid	p15A, Kan^R	This study
pBAD1K-*vcdP*	pMD087	*vcdP* expression plasmid	p15A, Kan^R	This study
pEVS143-*oppZ*	pMD090	*oppZ* expression plasmid	p15A, Kan^R	(Hoyos et al, 2020)
pEVS143-*tfoR*	pMD104	*tfoR* expression plasmid	p15A, Kan^R	This study
pEVS143-*vcr078*	pMD105	*vcr078* expression plasmid	p15A, Kan^R	This study
pEVS143-*vcdP*	pMD111	*vcdP* expression plasmid	p15A, Kan^R	This study

pXG10-*ptsG*	pMD161	Translational reporter for *ptsG*	pSC101*, Cm^R	This study
pXG10-*nagE*	pMD162	Translational reporter for *nagE*	pSC101*, Cm^R	This study
pXG10-*ptsHI*	pMD164	Translational reporter for *ptsHI*	pSC101*, Cm^R	This study
pEVS143-*vcdRP M2*	pMD389	*vcdRP M2* expression plasmid	p15A, Kan^R	This study
pXG10-*treB M2**	pMD401	Translational reporter for *treB M2**	pSC101*, Cm^R	This study
pXG10-*ptsG M2**	pMD402	Translational reporter for *ptsG M2**	pSC101*, Cm^R	This study
pXG10-*nagE M2**	pMD403	Translational reporter for *nagE M2**	pSC101*, Cm^R	This study
pXG10-*ptsHI M2**	pMD405	Translational reporter for *ptsHI M2**	pSC101*, Cm^R	This study
pET15b-*gltA*	pMD408	Over-expression construct for GltA purification	pBR322, Amp^R	This study
pEVS143-*vcr002*	pNP001	*vcr002* expression plasmid	p15A, Kan^R	This study
pEVS143-*micV*	pNP002	*micV* expression plasmid	p15A, Kan^R	(Peschek et al, 2019)
pEVS143-*vcr036*	pNP003	*vcr036* expression plasmid	p15A, Kan^R	This study
pEVS143-*vcr043*	pNP004	*vcr043* expression plasmid	p15A, Kan^R	This study
pEVS143-*vadR*	pNP005	*vadR* expression plasmid	p15A, Kan^R	(Peschek et al, 2020)
pXG10-*treB*	pNP0058	Translational reporter for *treB*	pSC101*, Cm^R	This study
pEVS143-*vcr079*	pNP006	*vcr079* expression plasmid	p15A, Kan^R	This study
pEVS143-*vcr065*	pNP007	*vcr065* expression plasmid	p15A, Kan^R	This study
pEVS143-*vcr034*	pNP008	*vcr034* expression plasmid	p15A, Kan^R	This study
pEVS143-*vcdRP*	pNP009	*vcdRP* expression plasmid	p15A, Kan^R	This study
pEVS143-*vcr092*	pNP010	*vcr092* expression plasmid	p15A, Kan^R	This study
pEVS143-*vcr098*	pNP011	*vcr098* expression plasmid	p15A, Kan^R	This study
pEVS143-*vcr103*	pNP012	*vcr103* expression plasmid	p15A, Kan^R	This study
pEVS143-*carZ*	pNP015	*carZ* expression plasmid	p15A, Kan^R	(Hoyos et al, 2020)
pEVS143-*vrrA*	pRH001	*vrrA* expression plasmid	p15A, Kan^R	(Peschek et al, 2019)
pEVS143-*qrr2*	pRH002	*qrr2* expression plasmid	p15A, Kan^R	This study
pEVS143-*qrr4*	pRH003	*qrr4* expression plasmid	p15A, Kan^R	This study
pEVS143-*gcvB*	pRH006	*gcvB* expression plasmid	p15A, Kan^R	This study
pEVS143-*csrD*	pRH009	*csrD* expression plasmid	p15A, Kan^R	This study
pEVS143-*flaX*	pRH010	*flaX* expression plasmid	p15A, Kan^R	This study
pKAS32-Δ*crp*	pRH023	Suicide plasmid for *crp* knockout	R6K, Amp^R	This study
pKAS32-Δ*cyaA*	pRH024	Suicide plasmid for *cyaA* knockout	R6K, Amp^R	This study
pEVS143-*vcr080*	pSG002	*vcr080* expression plasmid	p15A, Kan^R	This study
pEVS143-*vcdRP 256nt*	pSG006	*vcdRP* expression plasmid truncated at 256th nucleotide (from the 3' end)	p15A, Kan^R	This study

pEVS143-*vcdRP 156nt*	pSG008	*vcdRP* expression plasmid truncated at 156th nucleotide (from the 3' end)	p15A, Kan^R	This study
pEVS143-*vcr006*	pYH002	*vcr006* expression plasmid	p15A, Kan^R	This study
pCMW1C-mKate2	pYH010	Template for transcriptional reporters	p15A, Cm^R	Lab collection

Appendix Table S5: DNA oligonucleotides used in this study.

Sequences are given in 5' →3' direction; 'P-' denotes a 5' monophosphate

ID	Sequence	Description
KPO-0009	CTACGGCGTTTCACTTCTGAGTTC	*E.coli* 5S rRNA oligoprobe
KPO-0092	CCACACATTATACGAGCCGA	pEVS143-derivates
KPO-0196	GGAGAAACAGTAGAGAGTTGCG	pBAD1K-derivates
KPO-0243	TTCGTTTCACTTCTGAGTTCGG	*V.ch.* 5S rRNA oligoprobe
KPO-0267	TAATAGGCCTAGGATGCATATG	pKAS32-derivates
KPO-0268	CGTTAACAACCGGTACCTCTA	pKAS32-derivates
KPO-0456	P-CAGAGCATGAGTTGCATGAC	pKP333
KPO-0465	GTTTTTGGATCCAGCTTATCTTGCCTATTCGG	pKP333
KPO-0570	GGCTGAAAGCGATAATGATCTTG	*vc1449* qRT-PCR
KPO-0571	CGGCTTCCATTCTAGGATCTTC	*vc1449* qRT-PCR
KPO-0999	P-ACCACTGCTTTTTCTTAGAAGAC	pNP02
KPO-1000	GTTTTTTCTAGAGGATTAGAACCCGAATTAAACT	pNP02
KPO-1001	P-TCACAGAACCGCTGTGACCA	pNP10
KPO-1002	GTTTTTTCTAGATTGACTACTTCATTCGCCAC	pNP10
KPO-1003	P-GCAAACACATTGGTAAGATATTAG	pNP01
KPO-1004	GTTTTTTCTAGATATAACCTGTTCAGAATGTGCT	pNP01
KPO-1005	P-GTCATCTCGTTAGTCATTACGA	pNP04
KPO-1006	GTTTTTTCTAGACACTGACAAACCGGTGTTGG	pNP04
KPO-1007	P-GTAAGGTTAGTGAGAACATTTCT	pNP11
KPO-1008	GTTTTTTCTAGAAGTTTCAAATTTCGTGGACAGC	pNP11
KPO-1009	P-ACTTACTTGGATAAATATGCATTG	pNP08
KPO-1010	GTTTTTTCTAGAGTATTGTTTGTCTGTCATAAAGTT	pNP08
KPO-1011	P-ACCTGTCGCTAATTTCAGTATC	pNP07
KPO-1012	GTTTTTTCTAGAAGCCTAACCCTATCTTTCGT	pNP07
KPO-1013	P-TATTACAACAAGAGAGGCTCAA	pNP015
KPO-1014	GTTTTTTCTAGACAGACGCTACATCAAACTGAA	pNP015
KPO-1015	P-AATAGACAACCTTTTGTCCTATC	pNP05
KPO-1016	GTTTTTTCTAGAATAGAAAGCACTGAGTCAGGA	pNP05

KPO-1021	P-GTTTGAACCCCGGCGGCT	pNP06
KPO-1022	GTTTTTTCTAGAAAACCGACTCCTTGCAAGAA	pNP06
KPO-1023	GTTTTTTCTAGAGGATCCGGTGATTGATTGAG	pEVS143 derivates
KPO-1024	P-ACCCAAAGGGTAGAGCAAAC	pNP03
KPO-1025	GTTTTTTCTAGAGAAAACGAAGTAATCTTCACCTT	pNP03
KPO-1026	P-TGAATAATCAAAGACGAGGCTC	pNP12
KPO-1027	GTTTTTTCTAGAGAACAGCCAGTTAACTTGAGA	pNP12
KPO-1070	P-ACATGAGCGGTTACCTCAT	pAS03
KPO-1071	GTTTTTTCTAGATTATAGAGATAGGTTTGTGTGTG	pAS03
KPO-1072	P-GGTTTAGCACTCCCCCCTA	pAS02
KPO-1073	GTTTTTTCTAGAGTTTTTGTCTTTAGGAAAATAAAG	pAS02
KPO-1076	P-GCGTAGGGTACAGAGGTAA	pAS01
KPO-1077	GTTTTTTCTAGAAGTGCCAACGTGGAATAGC	pAS01
KPO-1082	P-GTGATTGACAGAGCTTTGAGA	pRH01
KPO-1083	GTTTTTTCTAGATCGCCAATGAACCGACTTG	pRH01
KPO-1084	P-GCAACGGCGGCCTGAACGG	pRH06
KPO-1085	GTTTTTTCTAGAAGCTCAGTATTTACTGGTTGGA	pRH06
KPO-1090	P-TGACCCTTCTAAGCCGAGG	pRH03
KPO-1091	GTTTTTTCTAGACCACGAAAGCCAAGATGCT	pRH03
KPO-1092	P-ACAAAGTATCACAAAAATCAGGG	pRH02
KPO-1093	GTTTTTTCTAGAAAAGCAGTGAAAATAGCGGG	pRH02
KPO-1219	P-AGCTTCGCTAGCGAAGAG	pNP09
KPO-1220	GTTTTTTCTAGAGAATGTTGCGATCAAGTTCG	pNP09
KPO-1226	TCGTATAATGTGTGGGTAAGGTTAGTGAGAACATTTCT	pRH05
KPO-1227	ACCGGATCCTCTAGAAGTTTCAAATTTCGTGGACAGC	pRH05
KPO-1282	GATCGCATTACGTAGACTACC	pMD054
KPO-1283	GTTCGAATAGGGAAGATTTTTTGAGACCACCAAGAAATTAAATTA CAACAC	pMD054
KPO-1284	CTCAAAAAATCTTCCCTATTCGAAC	pMD054
KPO-1285	GTTAAACCAGTACGGCCACC	pMD054
KPO-1286	GTTTTTGGTACCGAATTCGACTAAGCGCAGATC	pMD054
KPO-1287	GTTTTTCCTAGGGTGGTGTTTGAAGTACCGAGC	pMD054
KPO-1375	TCGTATAATGTGTGGGTCAGCAGGAAGCGGACAC	pRH09
KPO-1376	ACCGGATCCTCTAGATACGGAAAGATGCCAAGAGA	pRH09
KPO-1383	TCGTATAATGTGTGGTTGCCAAACTCTGCAATCTCG	pRH10
KPO-1384	ACCGGATCCTCTAGAAGTAGGAAAGATAAAGGTGGGG	pRH10
KPO-1397	GATCCGGTGATTGATTGAGC	pBAD1K-derivates
KPO-1440	TAGAGGTACCGGTTGTTAACGCTTCGAGCTTGTATTCTGC	pMD003
KPO-1441	CATATGCATCCTAGGCCTATTACAGAGAAGGGCTCAAACG	pMD003
KPO-1442	GCTGAAAGACACGGTTTCtTCCCTCTCAAAGAAATCGC	pMD003
KPO-1443	GCGATTTCTTTGAGAGGGAaGAAACCGTGTCTTTCAGC	pMD003

KPO-1448	TCGGCTCGTATAATGTGTGGACAGAATGAGTAATCAACCAAAG	pYH02
KPO-1449	CTCAATCAATCACCGGATCCCAATAAAAAAGGACGCGATGC	pYH02
KPO-1484	GCTCAATCAATCACCGGATCAAGGCCCAGTCTTTCGAC	pMD004, pMD111
KPO-1485	CGCAACTCTCTACTGTTTCTCCTTTTTTCTAGATTAAATCAGAACGCAG	pMD004
KPO-1488	TTTTTTCTAGATTAAATCAGAACGCAG	pKV176
KPO-1492	GATAAAACGAAAGGCCCAGTCTTTCGACTGAGCCTTTCG	pMD080
KPO-1524	CAACGGGAATCCTGCTCTG	pMD083
KPO-1525	GCGGCCCTCTCACTTCC	pMD083
KPO-1702	ATGCATGTGCTCAGTATCTCTATC	pXG10SF-derivates
KPO-1703	GCTAGCGGATCCGCTGG	pXG10SF-derivates
KPO-1708	GAGATACTGAGCACATGCATAATTGATTTGGGACTGTTCCCAA	pNP58
KPO-1709	GAGCCAGCGGATCCGCTAGCCAATTCGATAAGACGCGTCAC	pNP58
KPO-1860	TCGGCTCGTATAATGTGTGGGCAAGTCAGTGGTGTTGG	pSG02
KPO-1861	CTCAATCAATCACCGGATCCGTACTGTCAATATCGACCAC	pSG02
KPO-1949	TCTAGAGGATCCGGTGATTG	pEVS143-derivates
KPO-2040	GTGAATCATATCGACCAAATTTG	VcdRP oligoprobe
KPO-2050	GTCAGCAGAATATGTGATACAGG	pKV159
KPO-2082	CTCAATCAATCACCGGATCCGAATGTTGCGATCAAGTTCG	pEVS143-*vcdRP* derivates
KPO-2083	TCGGCTCGTATAATGTGTGGCTCCATGGAACCGAGAAATC	pSG06
KPO-2085	TCGGCTCGTATAATGTGTGG GTACACCGGTAACTTTGCATAC	pSG08
KPO-2090	GATTTTAATGGTGATAGTTATGAATTAAGGTTTATCGTCTGCAATGTTC	pMD055
KPO-2091	GAACATTGCAGACGATAAACCTTAATTCATAACTATCACCATTAAAATC	pMD055
KPO-2100	CGCAACTCTCTACTGTTTCTCCAGCTTCGCTAGCGAAGAG	pMD072
KPO-2101	GCTCAATCAATCACCGGATCGAATGTTGCGATCAAGTTCG	pMD072
KPO-2109	TCGGCTCGTATAATGTGTGGTTTTGTTCACCCCTAAATTGG	pMD062
KPO-2110	TCGGCTCGTATAATGTGTGGATTGGAATTTATTGACGACCAAATTTG	pMD063
KPO-2111	GTTTTTGCATGC GTAGGCAAATGCATCTTCATGC	pMD064
KPO-2112	GTTTTTGTCGACGAAGCTGTACACAAATATACCAC	pMD064
KPO-2229	CCTGTATCACATATTCTGCTGAC	pKV159
KPO-2243	AGAGGTACCGGTTGTTAACGGGAAGGTGTCTAAGTTAGCAC	pRH023
KPO-2244	TTAAATCTAGCGCATCATACCGTGTCAGTAGTCCAATTTGC	pRH023
KPO-2245	GTATGATGCGCTAGATTTAACGG	pRH023
KPO-2246	TATGCATCCTAGGCCTATTAGATACAACGCTGCTTCCTGC	pRH023
KPO-2249	AGAGGTACCGGTTGTTAACGCAGCATGGTTAAGAGGTGCG	pRH024
KPO-2250	GACGCATAATAAAAAAGAGCCTGATATGACAGATAAAAGCCGC	pRH024
KPO-2251	GCTCTTTTTTATTATGCGTCAGTG	pRH024
KPO-2252	TATGCATCCTAGGCCTATTACTCCGATTCCACGTTAAAGC	pRH024

KPO-2259	TCGGCTCGTATAATGTGTGGTACCCGTTTTTTGGGCTAACAGGAGGAATTAACC	pMD080
KPO-2260	GCCTCTAGATTATGAGACCAGGTCTCACATGGTTAATTCCTCCTGTTAGC	pMD080
KPO-2261	TGGTCTCATAATCTAGAGGCATCAAATAAAACGAAAGGCTCAGTCGAAAG	pMD080
KPO-2322	GTCTCGAACAAGTTTTGTTCATAAATTGGAATTTATTGACGACC	pMD083
KPO-2323	TGAACAAAACTTGTTCGAGAC	pMD083
KPO-2329	ACTGGGCCTTTCGTTTTATCTCTAGAGGATCCGGTGATTGATTGAGC	pMD080
KPO-2330	CTAACAGGAGGAATTAACCATGAACAAGGGCCTGAGTAGCGCTATGTTTTGGAATCAGCA	pMD111
KPO-2356	CCAAAAGAGCGTGGATAAGC	$VcdP_{scram}$ oligoprobe
KPO-2378	GGTAACCCAGAAACTACCACTG	*recA* qRT-PCR
KPO-2379	CACCACTTCTTCGCCTTCTT	*recA* qRT-PCR
KPO-2410	ATAACCAAAAGAGCGTGGATAAG	pKV114
KPO-2553	GCTCAATCAATCACCGGATCAGGCGATTGGTCGTGTTG	pMD090
KPO-2565	GCTCAATCAATCACCGGATCATTGAAGTGAGTGATGGTAATAG	pMD104
KPO-2568	TCGGCTCGTATAATGTGTGGATTCAGCCTGTGACGGG	pMD090
KPO-2570	TCGGCTCGTATAATGTGTGGGTTGAAAGGACATCCCTCC	pMD104
KPO-2571	TCGGCTCGTATAATGTGTGGATCAAAGATACAGACTTTGCC	pMD105
KPO-2572	GCTCAATCAATCACCGGATCCTTTTTAGGTTTCTCTGCCACA	pMD105
KPO-2715	GACCTGTTCCTGCTTTGTTTAC	*lamB* qRT-PCR
KPO-2716	CTGCTTTCGCTGTCGATTTC	*lamB* qRT-PCR
KPO-2747	AAGCAGTCAGGTGGTCTTATG	*ctxA* qRT-PCR
KPO-2748	ACAAATCCCGTCTGAGTTCC	*ctxA* qRT-PCR
KPO-2779	GAGATACTGAGCACATGCATCGGAAAATATAATGCAAAAAGTGG	pMD161
KPO-2780	GAGCCAGCGGATCCGCTAGCGATTAAGTTATTAGAATTGCTGGG	pMD161
KPO-2781	GAGATACTGAGCACATGCATGACTAAATTGGGCGACTAAAAAAAG	pMD162
KPO-2782	GAGCCAGCGGATCCGCTAGCCGTAGCGATAGGTAGCATC	pMD162
KPO-2792	GAGATACTGAGCACATGCATGTTACCAAGTTCAGGTGAACG	pMD164
KPO-2794	GAGCCAGCGGATCCGCTAGCTTGAAGAAGTAATGCTTTACCAATAG	pMD164
KPO-3236	TCTAGAGGCATCAAATAAAACGAAAGGC	pKV175
KPO-3726	CAGCCTAATCCAATAACGTGAAAC	Spot42 oligoprobe
KPO-4168	GACTACAAAGACCATGACGG	pKV114
KPO-4202	ATGGCTGCCGCGCGGC	pMD408
KPO-4203	GGATCCGGCTGCTAACAAAG	pMD408
KPO-4715	CCGTCATGGTCTTTGTAGTCGAGCTCACCCTGAAAATACAAATTC	pKV114
KPO-4716	CTTATCCACGCTCTTTTGGTTATTCCATGGAAAAGAGAAGATGG	pKV114
KPO-5266	TTCTGCCGATCTGTTCTACAC	*vca0053* qRT-PCR
KPO-5267	AGCCGCTACACCGTAGATA	*vca0053* qRT-PCR

KPO-5268	CTCGGCAATGTGATGATGTTTG	*vc2761* qRT-PCR
KPO-5269	CCAATGTCCCGTGCTTGATA	*vc2761* qRT-PCR
KPO-5270	GCCTCTTTAGGTGTGGAATTGA	*vc1953* qRT-PCR
KPO-5271	CATCGACGTGTTCGGTTTCT	*vc1953* qRT-PCR
KPO-6306	TAGAGGTACCGGTTGTTAACGATGGTTATGGCGGATAAGAAAGC	pKV150, pKV155
KPO-6307	CCGTCATGGTCTTTGTAGTCACTGCGTTCATGCAAGGGTG	pKV150
KPO-6308	CGACTACAAAGATGACGATAAATAGTCATTGATTCAAAAGCAAAA GAGCG	pKV150
KPO-6309	CATATGCATCCTAGGCCTATTAGGCAGTTGGTGGTTGAGCA	pKV150, pKV155
KPO-6476	TTTGTTCACGGCTAAATTGGAATTTATTGACGACC	pMD389
KPO-6477	CCAATTTAGCCGTGAACAAAACTTGTTCGAGAC	pMD389
KPO-6480	GCTACGGCCGTATGCCGTTAGTCCCAG	pMD402
KPO-6481	GCATACGGCCGTAGCCTAAATGTTAGCAC	pMD402
KPO-6484	CTAAGCGTAATCTGGAACATCGTATGGGTAACTGCGTTCATGCA AGGGTG	pKV152
KPO-6485	TGTTCCAGATTACGCTTAGTCATTGATTCAAAAGCAAAAGAGCG	pKV152
KPO-6486	CTAGTGATGGTGATGGTGATGACTGCGTTCATGCAAGGGTG	pKV153
KPO-6487	CACCATCACCATCACTAGTCATTGATTCAAAAGCAAAAGAGCG	pKV153
KPO-6524	TAGAGGTACCGGTTGTTAACGCATAGGCCAATCCAGCCATG	pKV154
KPO-6525	CTCCTTTGTTTATTATTTAATCCGTC	pKV154
KPO-6526	GACGGATTAAATAATAAACAAAGGAGTCATTGATTCAAAAGCAAA AGAGCG	pKV154
KPO-6527	CATATGCATCCTAGGCCTATTACCCTGATAGTCTGGCCACTG	pKV154
KPO-6558	AACTCATTGCCGTATAAAACATGTACGAGAAGC	pMD405
KPO-6559	GTTTTATACGGCAATGAGTTTATTTTTTGTAAGTTTG	pMD405
KPO-6560	AATTCAATACCGTGGACGAGGATATGAGC	pMD401
KPO-6561	CGTCCACGGTATTGAATTATTATCATTGCAGCAA	pMD401
KPO-6562	ATCCTATAGCCGGAACTTAAGGTGAATATTCTTG	pMD403
KPO-6563	TAAGTTCCGGCTATAGGATTTTTGTATAGTATTG	pMD403
KPO-6588	CCGCGCGGCAGCCATATGGTTATGGCGGATAAGAAAG	pMD408
KPO-6589	CTTTGTTAGCAGCCGGATCCTTAACTGCGTTCATGCAAGGG	pMD408
KPO-6669	GTCTATGGCGACGGTGATCTTTGCGATGTCTC	pKV155, pKV156
KPO-6670	GATCACCGTCGCCATAGACACAGGAATACCAATGG	pKV155, pKV156
KPO-6768	GAACCAACACGCGGCAGCGGCGGGTAACTTTGCATACCCTCG	pKV159
KPO-6769	GTTACCCGCCGCTGCCGCGTGTTGGTTCCAGAACATTGC	pKV159
KPO-6954	GACCAAGATCAATTTAAATGATTTTTTTAATCACCTTAAGTGTTG TAATTTA	pKV164
KPO-6955	AAAATCATTTAAATTGATCTTGGTCAG	pKV164
KPO-7127	CTCGAGCACCACCACCAC	pKV169
KPO-7130	TTATGTAAATCGCTCCTTTTTAGGTG	pKV169
KPO-7131	CACCTAAAAAGGAGCGATTTACATAAATACCCGTTTTTTGGGCTA ACAG	pKV169
KPO-7132	GTGGTGGTGGTGCTCGAGAAAACGAAAGGCCCAGTCTTTC	pKV169

KPO-7413	TCGTTTTATTTGATGCCTCTAGATTAACTGCGTTCATGCAAGGG	pKV175
KPO-7414	GCTAACAGGAGGAATTAACCATGGTTATGGCGGATAAGAAAGC	pKV175
KPO-7415	CTGCGTTCTGATTTAATCTAGAAAAAATTAACTGCGTTCATGCAA GGG	pKV176
KPO-7416	CGCAACTCTCTACTGTTTCTCCATGGTTATGGCGGATAAGAAAG C	pKV176
KPO-7417	GCTGCTCTGACTTCATTCCTAA	*ptsG* qRT-PCR
KPO-7418	CGTAAGCCAGACCTGCTAATAC	*ptsG* qRT-PCR
KPO-7419	GTGGTGCCTTCTTACCTCTATC	*treB* qRT-PCR
KPO-7420	CCAATCACGCGACCAAATG	*treB* qRT-PCR
KPO-7421	GTGATTGGTCTGGCGTTCTT	*nagE* qRT-PCR
KPO-7422	AGCGGAGTCTGTTTCTGTTTC	*nagE* qRT-PCR
KPO-7423	ACCTCTACAACCCACTCTCTC	*ptsH* qRT-PCR
KPO-7424	CCCGCTAATTCACCACACATA	*ptsH* qRT-PCR
KPO-7425	GCAAACGCTAGGTCTGGTAA	*ptsI* qRT-PCR
KPO-7426	GTTGGTCCATCAGAGCAACTA	*ptsI* qRT-PCR
KPO-IGRF	GGCTGCGAATTCCACAGATGACTCCTTACTGTAATTTTTCC	EMSA
KPO-IGRR	GCCCGAAGCTTCATGTTCGAATCCTTTAGTGGT	EMSA
pBAD-ATGrev	GGTTAATTCCTCCTGTTAGC	pEVS143 and pBAD1K-derivates

Appendix Supplementary References

Baumann P, Baumann L, Bang SS & Woolkalis MJ (1980) Reevaluation of the taxonomy of *Vibrio*, *Beneckea*, and *Photobacterium*: Abolition of the genus *Beneckea*. *Current Microbiology* 4: 127–132

Brockmeier U, Wendorff M & Eggert T (2006) Versatile Expression and Secretion Vectors for *Bacillus subtilis*. *Curr Microbiol* 52: 143–148

Burkholder PR & Giles NH (1947) Induced biochemical mutations in *Bacillus subtilis*. *Am J Bot* 34: 345–348

Corcoran CP, Podkaminski D, Papenfort K, Urban JH, Hinton JCD & Vogel J (2012) Superfolder GFP reporters validate diverse new mRNA targets of the classic porin regulator, MicF RNA. *Molecular Microbiology* 84: 428–445

Dunn AK, Millikan DS, Adin DM, Bose JL & Stabb EV (2006) New *rfp*- and pES213-Derived Tools for Analyzing Symbiotic *Vibrio fischeri* Reveal Patterns of Infection and *lux* Expression *In Situ*. *AEM* 72: 802–810

Hoyos M, Huber M, Förstner KU & Papenfort K (2020) Gene autoregulation by 3’ UTR-derived bacterial small RNAs. *eLife* 9: e58836

Papenfort K, Förstner KU, Cong J-P, Sharma CM & Bassler BL (2015) Differential RNA-seq of *Vibrio cholerae* identifies the VqmR small RNA as a regulator of biofilm formation. *Proc Natl Acad Sci USA* 112: E766–E775

Peschek N, Herzog R, Singh PK, Sprenger M, Meyer F, Fröhlich KS, Schröger L, Bramkamp M, Drescher K & Papenfort K (2020) RNA-mediated control of cell shape modulates antibiotic resistance in *Vibrio cholerae*. *Nature Communications* 11: 6067

Peschek N, Hoyos M, Herzog R, Förstner KU & Papenfort K (2019) A conserved RNA seed-pairing domain directs small RNA-mediated stress resistance in enterobacteria. *The EMBO Journal* 38: e101650

Simon R, Priefer U & Pühler A (1983) A Broad Host Range Mobilization System for *In Vivo* Genetic Engineering: Transposon Mutagenesis in Gram Negative Bacteria. *Bio/Technology* 1: 784–791

Skorupski K & Taylor RK (1996) Positive selection vectors for allelic exchange. *Gene* 169: 47–52

Svenningsen SL, Tu KC & Bassler BL (2009) Gene dosage compensation calibrates four regulatory RNAs to control *Vibrio cholerae* quorum sensing. *EMBO J* 28: 429–439

Thelin KH & Taylor RK (1996) Toxin-coregulated pilus, but not mannose-sensitive hemagglutinin, is required for colonization by *Vibrio cholerae* O1 El Tor biotype and O139 strains. *Infect Immun* 64: 2853–2856

Waters CM & Bassler BL (2006) The *Vibrio harveyi* quorum-sensing system uses shared regulatory components to discriminate between multiple autoinducers. *Genes Dev* 20: 2754–2767
