# Supplementary material for: A dual‐function RNA balances carbon uptake and central metabolism in Vibrio cholerae
Source: EMBO J. 2021 Oct 6;40(24):e108542. doi: 10.15252/embj.2021108542 (PMC8672173; doi:10.15252/embj.2021108542)
Supplement: Supplementary file 2 — Expanded View Figures PDF [file EMBJ-40-e108542-s009.pdf]

Expanded View Figures

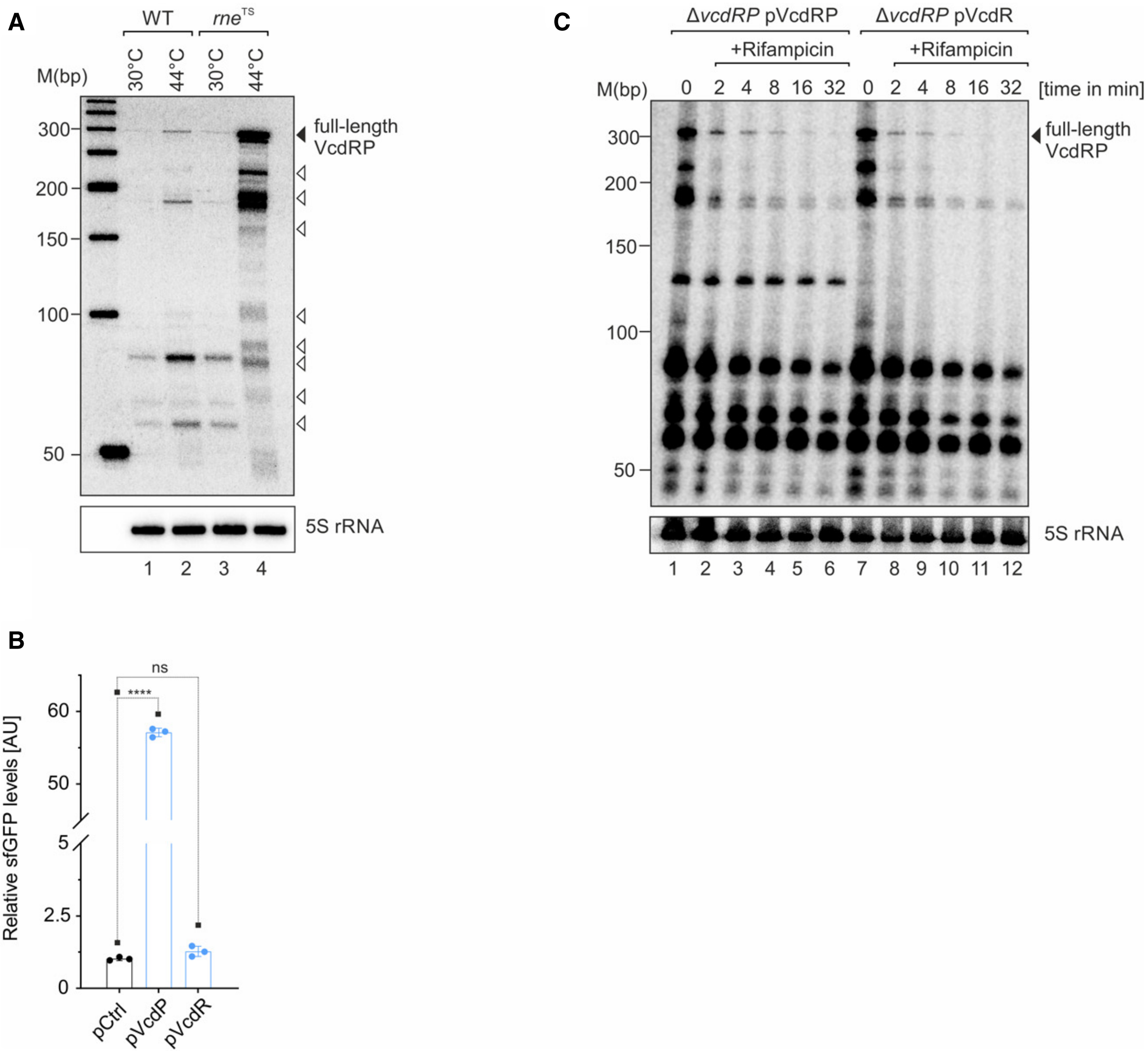

Figure EV1. Effect of VcdR/P variants on translation and RNA stability (related to Fig 1).

A RNase E-mediated processing of VcdRP. *V. cholerae* wild-type and *rne*^{TS} strains were grown at 30°C to stationary phase (OD_{600} of 2.0). Cultures were divided into half and were allowed to continue growing at either 30 or 44°C for 30 min. The cleavage patterns of VcdRP was monitored on a Northern blot. The solid triangle refers to the band corresponding to the full-length primary *vcdRP* transcript, whereas the open triangles correspond to the different processed isoforms. Probing with 5S rRNA confirmed equal loading.

B Relative fluorescence intensities (*y*-axis) of *E. coli* strains harboring an empty control plasmid (pCtrl) or translational fusions of *vcdRP* expression plasmids fused to *sfGFP*, expressing either only the 29 amino acid small protein (pVcdP) or a STOP codon introduced in the 3rd codon of the ORF (pVcdR, *x*-axis). Cells were grown in LB to OD_{600} of 1.0 and fluorophore production was measured. The fluorescence of pCtrl was set to 1.

C Northern blot analysis of *V. cholerae* Δ*vcdRP* strains harboring either pVcdRP or pVcdR plasmids examined for the stability of *vcdRP*. The cultures were grown in LB medium transcription was inhibited by the addition of rifampicin (f.c. 250 μg/ml) at OD_{600} of 1.0. RNA samples were harvested prior to, as well as at 2, 4, 8, 16, and 32 min post-rifampicin treatment. The solid triangle represents the full-length VcdRP transcript. Probing for 5S rRNA served as loading control.

Data information: Data in (B) are presented as mean ± SD, $n = 3$ independent biological replicates. Statistical significance was determined using one-way ANOVA and post hoc Tukey's multiple comparisons test. The *P*-value is summarized as follows - ns for $P > 0.05$ and **** for $P \leq 0.0001$.
Source data are available online for this figure.

© 2021 The Authors

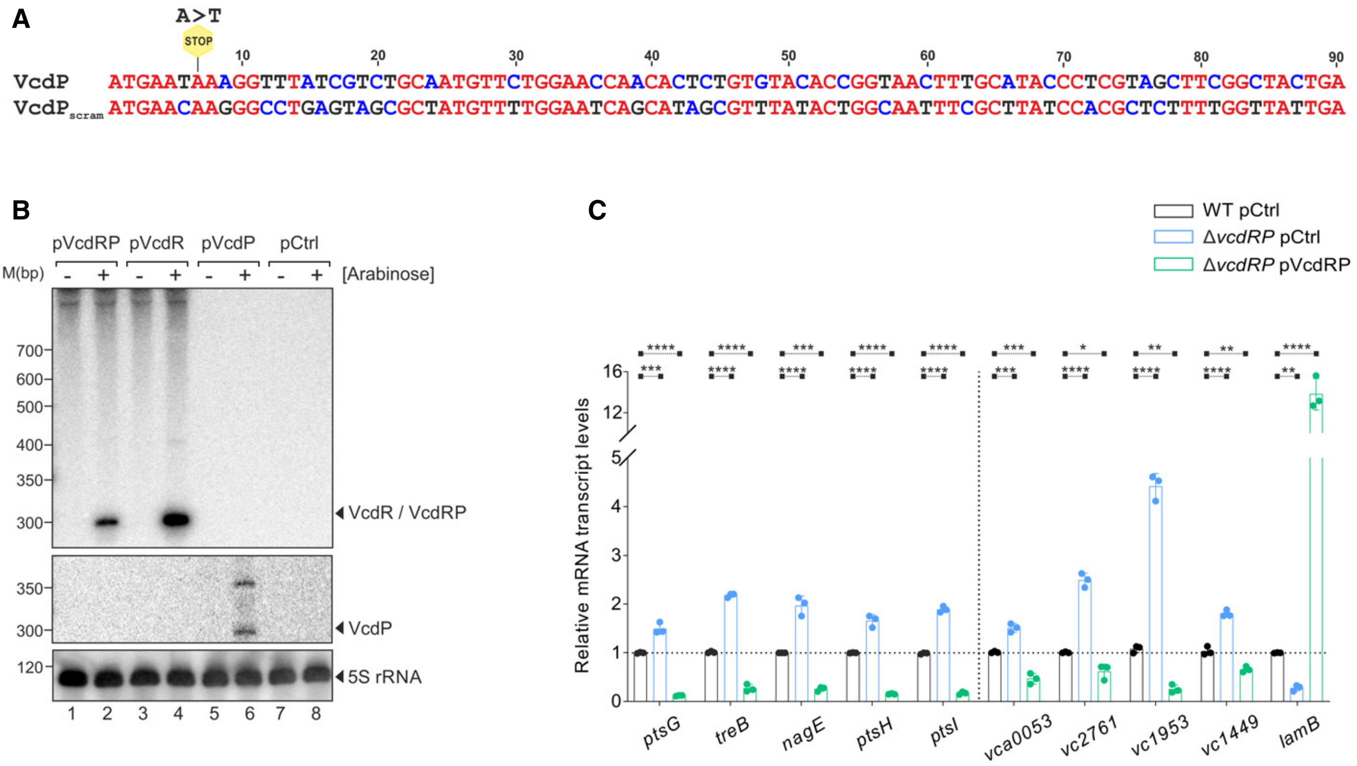

Figure EV2. VcdR/P variants used for the transcriptome analysis (related to Fig 3).

A To disentangle the protein-coding function from the base-pairing activity of *vcdRP*, the 3rd codon of the ORF was mutated from AAA to TAA (thus introducing a STOP codon, yellow). To generate the protein variant devoid of its RNA function, an over-expression plasmid was created carrying only the codon-modified *vcdP* ORF. The sequence of the VcdP codons were scrambled ($VcdP_{scram}$) such that the correct protein is translated, but the sequence of the two VcdR base-pairing regions is significantly changed.

B Northern blot analysis of *V. cholerae* Δ*vcdRP* strains carrying pBAD-based plasmids (pVcdRP, pVcdR, and pVcdP) or an empty vector control (pCtrl). RNA samples were harvested before (−) and after (+) L-arabinose induction for 15 min at OD_{600} of 0.1. The solid triangles indicate the expression of the different VcdR/P variants. Probing with 5S rRNA confirmed equal loading.

C qRT-PCR analyses of the indicated mRNA transcripts (x-axis) measured on *V. cholerae* wild-type and Δ*vcdRP* strains harboring an empty vector control (pCtrl) or Δ*vcdRP* carrying *vcdRP* expression plasmid (pVcdRP). The strains were grown in M9 medium supplemented with 0.4% glucose and 0.4% casaminoacids, and samples were collected at OD_{600} of 0.1. Relative fold changes were calculated with respect to wild-type pCtrl set to 1. *recA* served as the reference housekeeping gene for all the measurements.

Data information: Data in (C) are presented as mean ± SD, $n = 3$ independent biological replicates. Statistical significance was determined using one-way ANOVA and post hoc Dunnett's multiple comparisons test. The *P*-values are summarized as follows: ns for $P > 0.05$, * for $P \le 0.05$, ** for $P \le 0.01$, *** for $P \le 0.001$, and **** for $P \le 0.0001$.
Source data are available online for this figure.

 © 2021 The Authors

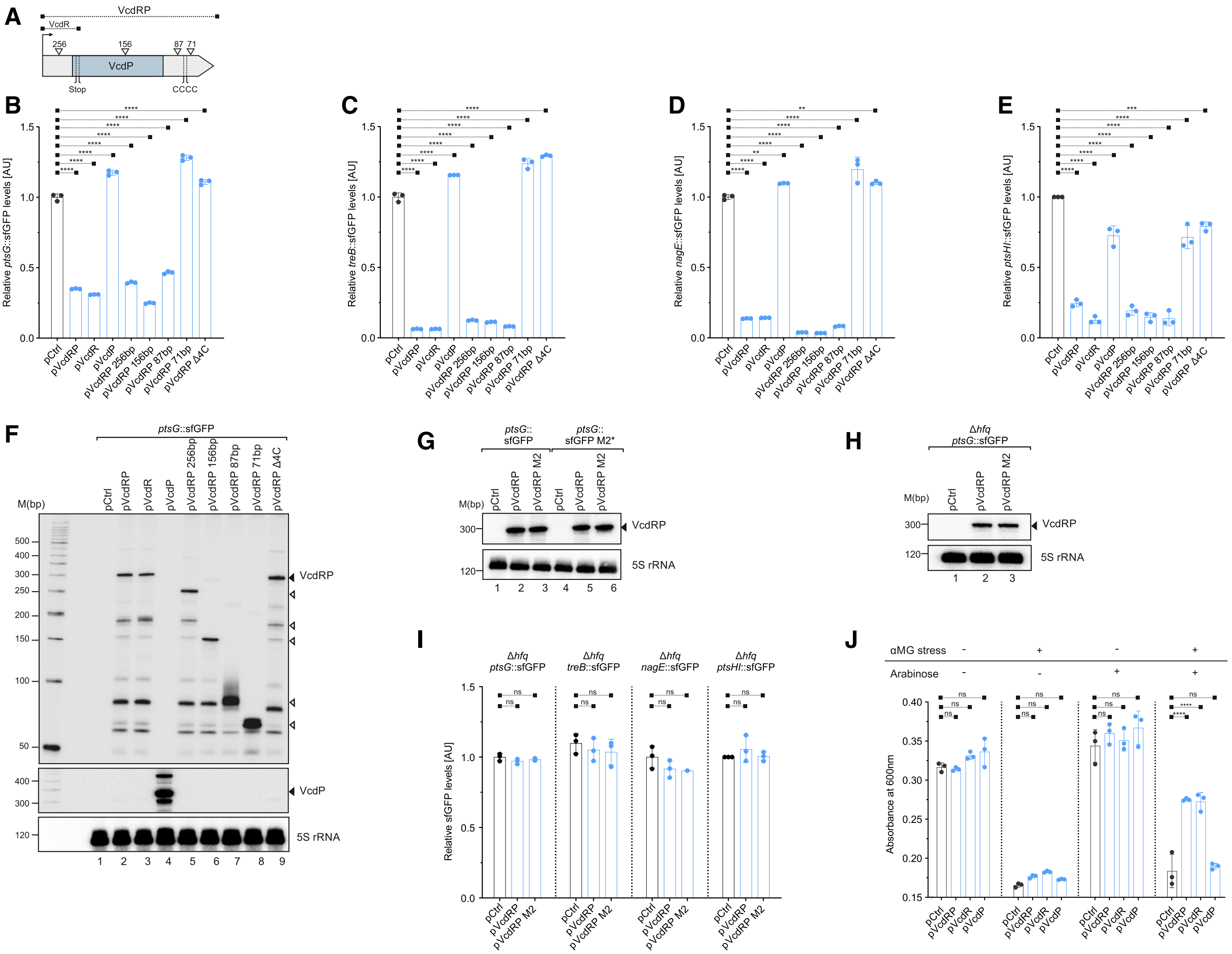

Figure EV3. Target spectrum of and post-transcriptional regulation by VcdR (related to Fig 4).

A A schematic representation of the different truncation plasmids of VcdRP tested in (B-F). These include pVcdRP, pVcdR, pVcdP, and pVcdRP Δ4C or plasmids truncated from the 5′ end maintaining the terminal 256, 156, 87, and 71 bp of *vcdRP*, respectively.

B–E Relative fluorescence intensities of *E. coli* translational reporters of *ptsG* (B), *treB* (C), *nagE* (D), and *ptsHI* (E) fused to *sfGFP* harboring either an empty control vector (pCtrl) or VcdRP expression plasmids described in (A) Cells were grown in LB to OD_{600} of 1.0 and fluorophore production was measured using plate reader (B-D) or by Western blotting (E). The fluorescence of pCtrl (for each reporter fusion) was set to 1.

F RNA samples were harvested in parallel to (B) and the corresponding expression was examined by Northern blotting. The solid triangle represents the band corresponding to the full-length primary *vcdRP* transcript, whereas the open triangles indicate the sizes of the respective truncations tested in (B). Probing with 5S rRNA confirmed equal loading.

G Northern blot analysis of *E. coli* translational reporter of *ptsG* or the corresponding M2* variant, combined with an empty control plasmid or *vcdRP* expression plasmids (pVcdRP and pVcdRP M2). The solid triangle represents the band corresponding to the full-length primary *vcdRP* transcript. Probing with 5S rRNA confirmed equal loading.

H Northern blot analysis of a translational reporter of *ptsG* fused to *sfGFP* monitored in *E. coli* cells lacking *hfq*, combined with an empty control plasmid or *vcdRP* expression plasmids (pVcdRP and pVcdRP M2). The solid triangle represents the band corresponding to the full-length primary *vcdRP* transcript. Probing with 5S rRNA confirmed equal loading.

I Relative fluorescence intensities of translational reporters of *ptsG*, *treB*, *nagE*, and *ptsHI* fused to *sfGFP* monitored in *E. coli* cells lacking *hfq*, harboring either an empty control vector (pCtrl) or VcdRP expression plasmids (pVcdRP and pVcdRP M2). Cells were grown in LB to OD_{600} of 1.0, and fluorophore production was measured using plate reader (for *ptsG*, *treB*, and *nagE*) or by Western blotting (for *ptsHI*). The fluorescence of pCtrl (for each reporter fusion) was set to 1.

J The base-pairing element of the dual regulator confers protection against the sugar analog αMG. *V. cholerae* Δ*vcdRP* strain harboring either an empty vector control (pCtrl) or inducible *vcdRP* expression plasmids (pVcdRP, pVcdR, pVcdP on *x*-axis) was grown to early log phase at which 0.1% αMG and / or 0.2% arabinose was added. Absorbance at 600 nm (*y*-axis) was measured after 5 h of growth without (−) or with (+) αMG and/or arabinose (indicated in the upper panel).

Data information: Data in (B–E) and (I–J) are presented as mean ± SD, $n = 3$ independent biological replicates. Statistical significance was determined using one-way or two-way ANOVA and post hoc Dunnett's multiple comparisons test. The *P*-value is summarized as follows - ns for $P > 0.05$, ** for $P \le 0.01$, *** for $P \le 0.001$ and **** for $P \le 0.0001$. Source data are available online for this figure.

Figure EV4. Mechanism of regulation by VcdP and its interaction with citrate synthase (related to Fig 5).

A, B Relative fluorescence intensities (*y*-axis) of *E. coli* translational reporter of *vca0053* fused to *sfGFP* (A) or *V. cholerae* Δ*vcdRP* strain harboring the transcriptional reporter of the *vca0053* promoter fused to *mKate2* (B), combined with either an empty control vector (pCtrl) or VcdRP expression plasmids (pVcdRP, pVcdR, pVcdP, and pVcdP-SPA). Cells were grown in LB to OD_{600} of 1.0 and the corresponding fluorophore production was measured. The fluorescence of pCtrl (for each reporter fusion) was set to 1.

C Co-immunoprecipitation of chromosomally tagged GltA::HA combined with either an empty vector control (pCtrl) or SPA-tagged *vcdP* over-expression plasmid, grown in LB medium to exponential phase (OD_{600} of 0.5). Protein samples corresponding to the total input and cell lysates before and after subjecting to reciprocal immunoprecipitation with anti-HA antibody. RNAP served as loading control. The solid triangles indicate the corresponding protein sizes.

D, E Co-immunoprecipitation of chromosomally tagged GltA::6xHis combined with either an empty vector control (pCtrl) or SPA-tagged *vcdP* over-expression plasmid, subjected to either anti-Flag (D) or anti-His (E) as bait. The cultures were grown in LB medium to exponential phase (OD_{600} of 0.5). Western blot analysis for (C–E) with anti-HA or anti-His and anti-Flag antibodies confirmed the interaction of GltA with VcdP *in vivo*. RNAP served as loading control. The solid triangles indicate the corresponding protein sizes.

F Western blot analysis of GltA production (tagged chromosomally with HA), when combined with same set of plasmids in (A) and (B). Protein samples were collected from strains grown in LB medium to mid-log phase (OD_{600} of 0.5). RNAP served as loading control.

Data information: For (A) and (B), data are presented as mean ± SD, $n = 3$ independent biological replicates. Statistical significance was determined using one-way ANOVA and post hoc Tukey's multiple comparisons test. The *P*-value is summarized as follows—ns for $P > 0.05$, *** for $P \leq 0.001$, and **** for $P \leq 0.0001$.
Source data are available online for this figure.

 © 2021 The Authors

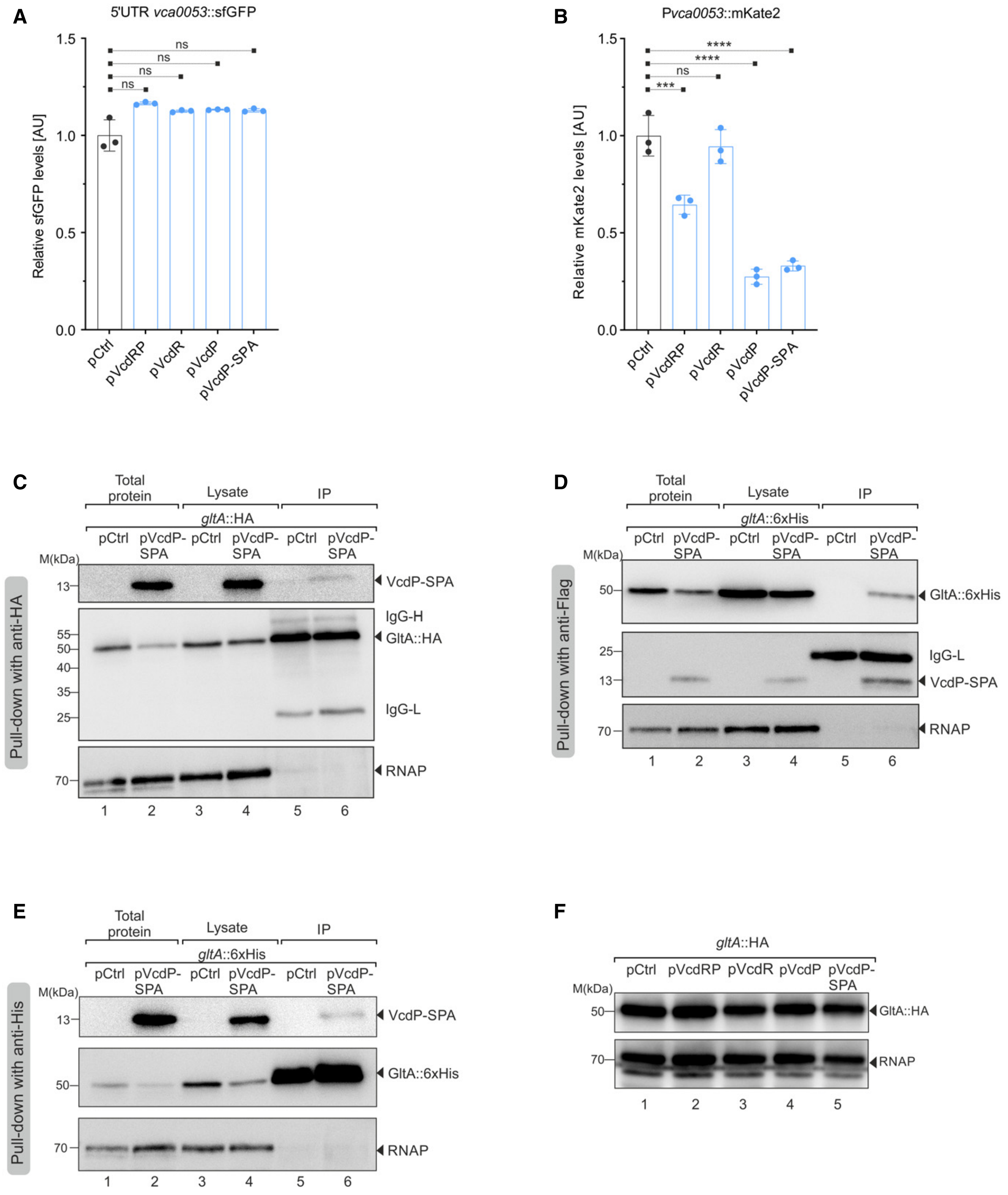
A
5'UTR vca0053::sfGFP
Relative sfGFP levels [AU]
ns
pCtrl
pVcdRP
pVcdR
pVcdP
pVcdP-SPA
B
Pvca0053::mKate2
Relative mKate2 levels [AU]
****
***
ns
C
Total protein
Lysate
IP
gltA::HA
M(kDa)
Pull-down with anti-HA
VcdP-SPA
IgG-H
GltA::HA
IgG-L
RNAP
D
gltA::6xHis
Pull-down with anti-Flag
GltA::6xHis
E
Pull-down with anti-His
F
GltA::HA

Figure EV4.

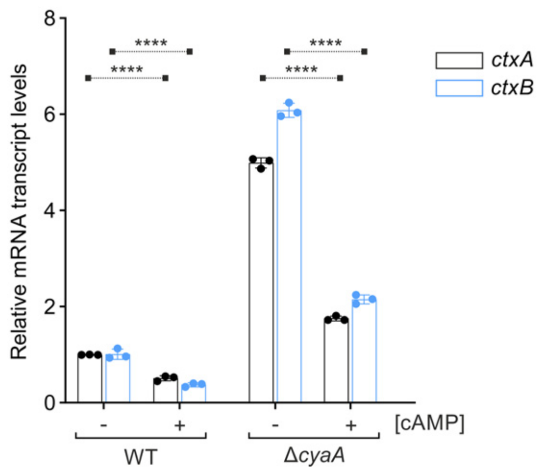

Figure EV5. Increased cAMP leads to reduction of *ctxAB* transcript levels (related to Fig 7).

V. cholerae wild-type and Δ*cyaA* strains were grown without (−) or with (+) cAMP (f.c. 5 mM) in AKI medium to stimulate production of CTX. Growth under AKI conditions involve biphasic cultures. In the first phase, the cultures were grown in a still tube for 4 h at 37°C. Subsequently, in the second phase, the cultures were poured into a flask to continue growing with shaking. RNA samples equivalent to OD_{600} of 2.0 were harvested after 16 h of continuous shaking followed by qRT analyses of the *ctxA* and *ctxB* transcripts. The fold changes of transcript levels were calculated relative to wild-type (−) cAMP sample set to 1. *recA* served as the reference housekeeping gene for these measurements.

Data information: Data are presented as mean ± SD, $n = 3$ independent biological replicates. Statistical significance was determined using one-way ANOVA and post hoc Tukey's multiple comparisons test. The *P*-values are summarized as follows: **** for $P \leq 0.0001$.
Source data are available online for this figure.

 © 2021 The Authors
