# Supplementary material for: A dual‐function RNA balances carbon uptake and central metabolism in Vibrio cholerae
Source: EMBO J. 2021 Oct 6;40(24):e108542. doi: 10.15252/embj.2021108542 (PMC8672173; doi:10.15252/embj.2021108542)
Supplement: Supplementary file 3 — Source Data for Expanded View and Appendix [file EMBJ-40-e108542-s004.zip › EMBOJ-2021-108542R_SourceDataForAppendixFigureS1B.pdf]

Source Data Fig. S1

Data related to Fig. S1B

Data refers to the mKate2 levels for each variant corrected for autofluorescence, calculated as relative fold change w.r.t. WT (set to 1)

Rel. mKate2 levels [AU]		Rep I	Rep II	Rep III
WT		0.9973	1.0227	1.1065
Δ*crp*		1.006	0.9895	0.9543
Δ*cyaA*		1.0144	0.9909	0.9780

Statistical analysis related to Fig. S1B

Normality test (Shapiro-Wilk)
Passed normality test (alpha=0.05)? Yes

Multiple comparisons
Number of families 1
Number of comparisons per family 2
Alpha 0.05

Dunnett's multiple comparisons test	Mean Diff.	95.00% CI of diff.	Below threshold?	Summary	Adjusted P Value
WT vs. Δ*crp*	0.05895	-0.04894 to 0.1668	No	ns	0.3297
WT vs. Δ*cyaA*	0.03879	-0.06909 to 0.1467	No	ns	0.6216
