# Supplementary material for: A dual‐function RNA balances carbon uptake and central metabolism in Vibrio cholerae
Source: EMBO J. 2021 Oct 6;40(24):e108542. doi: 10.15252/embj.2021108542 (PMC8672173; doi:10.15252/embj.2021108542)
Supplement: Supplementary file 3 — Source Data for Expanded View and Appendix [file EMBJ-40-e108542-s004.zip › EMBOJ-2021-108542R_SourceDataForAppendixFigureS2.pdf]

Source Data Fig. S2

Data related to Fig. S2

Data refers to the sfGFP levels for each reporter gene fusion corrected for autofluorescence, calculated as relative fold change w.r.t. pCtrl (set to 1)

Gene fusion	*lamB*::sfGFP			*vc0177*::sfGFP			*vc1779*::sfGFP		
Rel. sfGFP levels [AU]	Rep I	Rep II	Rep III	Rep I	Rep II	Rep III	Rep I	Rep II	Rep III
pCtrl	0.9943	1.0056	1.0001	0.9939	1.0369	0.9692	1.0632	0.9548	0.9821
pVcdRP	1.0780	1.0823	1.0712	0.9889	1.0129	0.8695	1.0836	0.9971	1.0134
pVcdR	1.1284	1.1456	1.1459	0.8970	0.9579	0.7983	1.1163	1.0632	1.0210
pVcdP	1.1221	1.1315	1.1267	0.8620	0.9710	0.8775	1.0963	1.2324	1.2073
pVcdRP Δ4C	1.2123	1.1912	1.1521	0.9227	1.0447	0.9597	1.1587	1.3070	1.2432

Statistical analysis related to Fig. S2

**ANOVA table**	**SS**	**DF**	**MS**	**F (DFn, DFd)**	**P value**
Interaction	0.1123	8	0.01403	F (8, 30) = 5.207	P=0.0004
Row Factor	0.09762	4	0.02441	F (4, 30) = 9.056	P<0.0001
Column Factor	0.2564	2	0.1282	F (2, 30) = 47.56	P<0.0001
Residual	0.08085	30	0.002695		

Normality test (Shapiro-Wilk)
Passed normality test (alpha=0.05)? Yes

Multiple comparisons
Number of families 3
Number of comparisons per family 4
Alpha 0.05

Dunnett's multiple comparisons test	Mean Diff.	95.00% CI of diff.	Below threshold?	Summary	Adjusted P Value
lamB::sfGFP					
pCtrl vs. pVcdRP	-0.03139	-0.1407 to 0.07790	No	ns	0.8713
pCtrl vs. pVcdR	-0.06686	-0.1761 to 0.04242	No	ns	0.3421
pCtrl vs. pVcdP	-0.1786	-0.2879 to -0.06935	Yes	***	0.0008
pCtrl vs. pVcdRP Δ4C	-0.2363	-0.3456 to -0.1270	Yes	****	<0.0001
vc0177::sfGFP					
pCtrl vs. pVcdRP	-0.07715	-0.1864 to 0.03213	No	ns	0.2287
pCtrl vs. pVcdR	-0.1400	-0.2493 to -0.03070	Yes	**	0.0088
pCtrl vs. pVcdP	-0.1268	-0.2361 to -0.01749	Yes	*	0.0191
pCtrl vs. pVcdRP Δ4C	-0.1852	-0.2945 to -0.07592	Yes	***	0.0005
vc1779::sfGFP					
pCtrl vs. pVcdRP	0.04291	-0.06637 to 0.1522	No	ns	0.7052
pCtrl vs. pVcdR	0.1156	0.006329 to 0.2249	Yes	*	0.0356
pCtrl vs. pVcdP	0.09647	-0.01281 to 0.2058	No	ns	0.0957
pCtrl vs. pVcdRP Δ4C	0.02429	-0.08499 to 0.1336	No	ns	0.9424
