# Supplementary material for: A dual‐function RNA balances carbon uptake and central metabolism in Vibrio cholerae
Source: EMBO J. 2021 Oct 6;40(24):e108542. doi: 10.15252/embj.2021108542 (PMC8672173; doi:10.15252/embj.2021108542)
Supplement: Supplementary file 3 — Source Data for Expanded View and Appendix [file EMBJ-40-e108542-s004.zip › EMBOJ-2021-108542R_SourceDataForAppendixFigureS4.pdf]

Source Data Fig. S4

Data refers to the peak area of each metabolite determined by mass spectrometry. For certain metabolites, only two biological replicates could be measured and therefore, the corresponding peak area has been left blank.

	Exponential phase					
Metabolite	Δ*gltA* + pCtrl			Δ*gltA* + pVcdP		
	Rep I	Rep II	Rep III	Rep I	Rep II	Rep III
Glucose	4.64E+05	6.46E+05	7.16E+05	4.89E+05	4.75E+05	5.25E+05
Glc6P	3.48E+05	5.43E+05	8.28E+05	2.92E+05	3.06E+05	2.82E+05
Frc6P	3.25E+06	4.79E+06	5.93E+06	3.59E+06	3.04E+06	3.38E+06
FrcBP	1.05E+06	9.86E+04	1.12E+06	8.08E+05	1.87E+06	4.60E+05
PEP	1.21E+05	1.85E+05	2.51E+05	1.65E+05	3.69E+05	3.18E+05
Pyruvate	1.62E+06	1.16E+06		4.34E+05	9.32E+05	8.98E+04
Alanine	2.58E+05	3.13E+05	4.25E+05	2.84E+05	3.02E+05	2.71E+05
AcetylCoA	4.50E+06	5.38E+05	2.49E+06	3.76E+06	1.10E+07	4.25E+05
Citrate	2.54E+08	3.29E+08	7.00E+08	2.68E+08	2.66E+08	2.54E+08
Cis-Aconitate	1.24E+07	1.62E+07	2.65E+07	1.14E+07	1.38E+07	9.68E+06
α-ketoglutarate	2.65E+05	1.14E+05	1.98E+06	6.01E+04	3.35E+05	
Glutamine	7.47E+05	6.66E+05	1.14E+06	6.10E+05	1.16E+06	1.15E+06
Glutamate	2.22E+08	2.83E+08	4.31E+08	2.50E+08	2.35E+08	2.22E+08
Succinate	8.85E+06	6.91E+06	2.19E+07	9.50E+06	9.68E+06	7.86E+06
Malate	7.31E+07	8.84E+07	1.49E+08	8.51E+07	7.29E+07	1.28E+08
Aspartate	9.21E+07	1.35E+08	1.95E+08	1.04E+08	1.04E+08	4.96E+07
Aspargine	2.45E+06	3.70E+06	3.83E+06	3.04E+06	2.47E+06	8.80E+05

	Stationary phase					
Metabolite	Δ*gltA* + pCtrl			Δ*gltA* + pVcdP		
	Rep I	Rep II	Rep III	Rep I	Rep II	Rep III
Glucose	1.22E+06	1.01E+06	1.01E+06	8.85E+05	1.09E+06	9.27E+05
Glc6P	9.64E+05	1.24E+06	6.88E+05	1.07E+06	1.81E+06	7.21E+05
Frc6P	2.69E+06	2.71E+06	2.24E+06	2.26E+06	4.39E+06	2.44E+06
FrcBP	6.33E+05	7.03E+05	3.27E+05	9.24E+05	1.56E+06	3.26E+05
PEP	1.12E+07	9.38E+06	4.53E+06	9.43E+06	1.83E+07	4.87E+06
Pyruvate	3.61E+05	5.60E+05	2.35E+05	3.29E+05	6.34E+05	1.95E+05
Alanine	2.80E+05	2.82E+05	2.62E+05	2.34E+05	3.56E+05	1.97E+05
AcetylCoA	1.98E+07	1.46E+07	1.37E+07	2.16E+07	2.52E+07	2.23E+07
Citrate	1.07E+07	5.60E+06	8.69E+06	8.07E+06	1.14E+07	7.53E+06
Cis-Aconitate	3.37E+06	4.19E+06	6.75E+06	1.02E+06	2.00E+06	5.12E+06
α-ketoglutarate	1.63E+05	2.85E+05	1.22E+05	1.65E+05	3.07E+05	2.56E+05
Glutamine	4.34E+06	5.32E+06	4.66E+06	3.51E+06	3.95E+06	3.77E+06
Glutamate	3.43E+07	4.33E+07	3.85E+07	2.80E+07	3.47E+07	3.22E+07

Succinate	9.56E+05	9.47E+05	2.90E+05	9.16E+05	1.50E+06	2.42E+05
Malate	5.91E+06	6.98E+06	1.32E+06	5.40E+06	1.04E+07	2.08E+06
Aspartate	6.82E+07	9.76E+07	6.83E+07	4.98E+07	7.64E+07	6.19E+07
Aspargine	1.41E+06	1.42E+06	1.30E+06	1.07E+06	1.39E+06	1.09E+06
