# Supplementary material for: A dual‐function RNA balances carbon uptake and central metabolism in Vibrio cholerae
Source: EMBO J. 2021 Oct 6;40(24):e108542. doi: 10.15252/embj.2021108542 (PMC8672173; doi:10.15252/embj.2021108542)
Supplement: Supplementary file 3 — Source Data for Expanded View and Appendix [file EMBJ-40-e108542-s004.zip › EMBOJ-2021-108542R_SourceDataForAppendixFigureS5A-B.pdf]

Source Data Fig. S5

Data related to Fig. S5A-B

Data refers to the citrate synthase activity levels calculated as nmol $min^{-1}mg^{-1}$

	Citrate synthase activity (nmol $min^{-1}mg^{-1}$)		
Lysates extracted from:	Rep I	Rep II	Rep III
V. nat pCtrl	142.1834	140.0507	146.4489
V. nat pVcdP	223.8167	232.0807	233.1137
B. sub pCtrl	22.50356	22.50356	28.50451
B. sub pVcdP	22.59059	22.59059	24.43472

Statistical analysis related to Fig. S5A-B

**ANOVA table**	**SS**	**DF**	**MS**	**F (DFn, DFd)**	**P value**
Treatment (between columns)	90446	3	30149	F (3, 8) = 2426	P<0.0001
Residual (within columns)	99.43	8	12.43		
Total	90545	11			

Equal variance test (Brown-Forsythe)

F (DFn, DFd)	0.3307 (3, 8)
P value	0.8036
P value summary	ns
Are SDs significantly different (P<0.05)?	No

Normality test (Shapiro-Wilk)

Passed normality test (alpha=0.05)?	Yes

Multiple comparisons

Number of families	1
Number of comparisons per family	6
Alpha	0.05

Tukey's multiple comparisons test	Mean Diff.	95.00% CI of diff.	Below threshold?	Summary	Adjusted P Value
V. nat pCtrl vs. *V. nat* pVcdP	-86.78	-95.99 to -77.56	Yes	****	<0.0001
V. nat pCtrl vs. *B. sub* pCtrl	118.4	109.2 to 127.6	Yes	****	<0.0001
V. nat pCtrl vs. *B. sub* pVcdP	119.7	110.5 to 128.9	Yes	****	<0.0001
V. nat pVcdP vs. *B. sub* pCtrl	205.2	195.9 to 214.4	Yes	****	<0.0001
V. nat pVcdP vs. *B. sub* pVcdP	206.5	197.2 to 215.7	Yes	****	<0.0001
B. sub pCtrl vs. *B. sub* pVcdP	1.299	-7.920 to 10.52	No	ns	0.9675
