# Supplementary material for: A dual‐function RNA balances carbon uptake and central metabolism in Vibrio cholerae
Source: EMBO J. 2021 Oct 6;40(24):e108542. doi: 10.15252/embj.2021108542 (PMC8672173; doi:10.15252/embj.2021108542)

# Source Data Fig. EV1

## Data related to Fig. EV1A

Upper panel
VcdRP (KPO-2040)

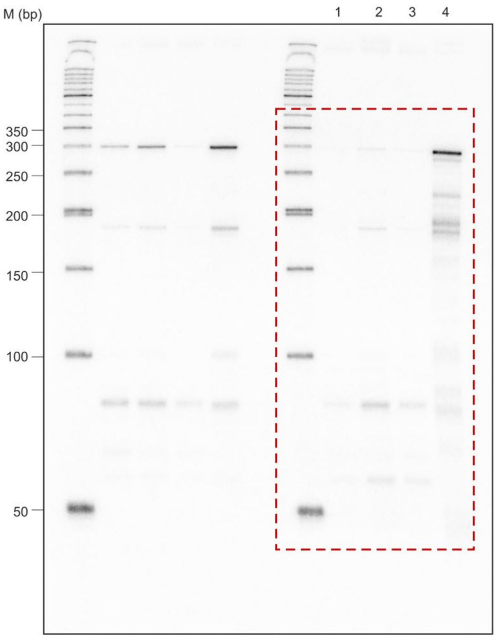


Lower panel
5S rRNA (KPO-0243)

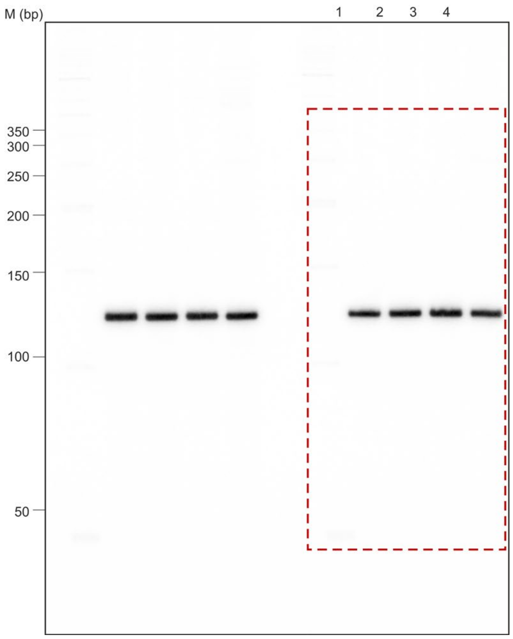


## Data related to Fig. EV1B

Data refers to the sfGFP levels for each variant corrected for autofluorescence, calculated as relative fold change w.r.t. pCtrl (set to 1)

| Rel. sfGFP levels [AU] | Rep I | Rep II | Rep III |
|---|---|---|---|
| pCtrl | 1.0837 | 1.0128 | 0.9647 |
| pVcdP | 54.4575 | 57.5957 | 57.2504 |
| pVcdR | 1.1070 | 1.2635 | 1.4635 |

## Statistical analysis related to Fig. EV1B

| **ANOVA table** | **SS** | **DF** | **MS** | **F (DFn, DFd)** | **P value** |
|---|---|---|---|---|---|
| Treatment (between columns) | 6261 | 2 | 3131 | F (2, 6) = 24915 | P<0.0001 |
| Residual (within columns) | 0.7539 | 6 | 0.1257 | | |
| Total | 6262 | 8 | | | |

**Equal variance test (Brown-Forsythe)**

| | |
|---|---|
| F (DFn, DFd) | 1.676 (2, 6) |
| P value | 0.2641 |
| P value summary | ns |
| Are SDs significantly different (P<0.05)? | No |

**Normality test (Shapiro-Wilk)**

| | |
|---|---|
| Passed normality test (alpha=0.05)? | Yes |

**Multiple comparisons**

| | |
|---|---|
| Number of families | 1 |

Number of comparisons per family 2
Alpha 0.05

| Dunnett's multiple comparisons test | Mean Diff. | 95.00% CI of diff. | Below threshold? | Summary | Adjusted P Value |
|---|---|---|---|---|---|
| pCtrl vs. pVcdP | -56.08 | -56.91 to -55.25 | Yes | **** | <0.0001 |
| pCtrl vs. pVcdR | -0.2576 | -1.086 to 0.5710 | No | ns | 0.6034 |

## Data related to Fig. EV1C

Upper panel
VcdRP (KPO-2040)

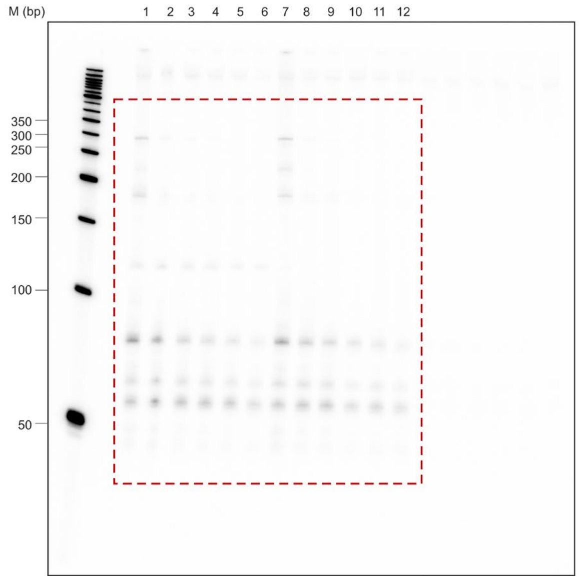


Lower panel
5S rRNA (KPO-0243)

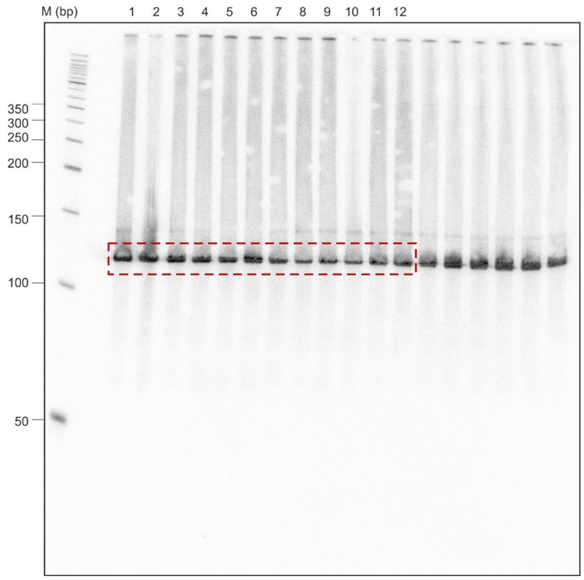

Supplement: Supplementary file 3 — Source Data for Expanded View and Appendix [file EMBJ-40-e108542-s004.zip › EMBOJ-2021-108542R_SourceDataForFigureEV1A-C.pdf]
