# Supplementary material for: A dual‐function RNA balances carbon uptake and central metabolism in Vibrio cholerae
Source: EMBO J. 2021 Oct 6;40(24):e108542. doi: 10.15252/embj.2021108542 (PMC8672173; doi:10.15252/embj.2021108542)
Supplement: Supplementary file 3 — Source Data for Expanded View and Appendix [file EMBJ-40-e108542-s004.zip › EMBOJ-2021-108542R_SourceDataForFigureEV2B-C.pdf]

Source Data Fig. EV2

Data related to Fig. EV2B

Upper panel
VcdRP (KPO-2040)

Middle panel
VcdP (KPO-2356)

Lower panel
5S rRNA (KPO-0243)

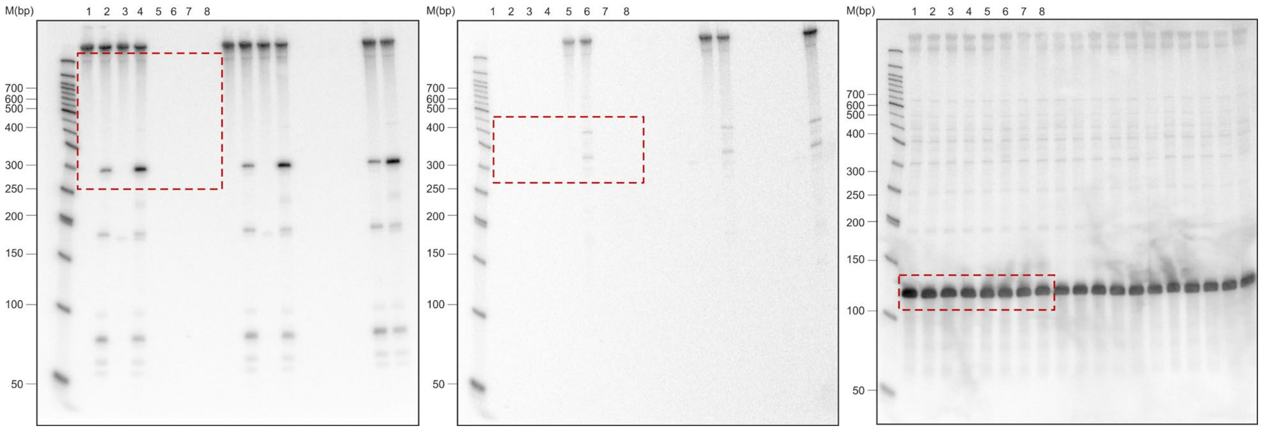

Data related to Fig. EV2C

Data refers to normalized mRNA levels w.r.t. the housekeeping gene *recA*. The mRNA levels have been calculated relative to WT pCtrl levels set to 1 (for each transcript).

Rel. mRNA levels		WT pCtrl	Δ*vcdRP* pCtrl	Δ*vcdRP* pVcdRP
ptsG	Rep I	0.9971	1.4241	0.1287
	Rep II	1.0125	1.6294	0.1274
	Rep III	0.9874	1.4387	0.1091
treB	Rep I	1.0021	2.2017	0.3598
	Rep II	1.0066	2.2069	0.2084
	Rep III	1.0325	2.1311	0.2467
nagE	Rep I	1.0012	2.1320	0.2876
	Rep II	1.0021	2.0229	0.2767
	Rep III	0.9987	1.7545	0.2055
ptsH	Rep I	0.9987	1.5182	0.1673
	Rep II	0.9937	1.7547	0.1502
	Rep III	1.0032	1.6967	0.1483
ptsI	Rep I	0.9932	1.8315	0.1993
	Rep II	0.9712	1.9568	0.1714
	Rep III	1.0021	1.8844	0.1445
vca0053	Rep I	1.0032	1.5187	0.4719
	Rep II	0.9987	1.4205	0.3559
	Rep III	1.0332	1.5970	0.5780
vc2761	Rep I	1.0231	2.3402	0.7116
	Rep II	1.0002	2.5021	0.4378
	Rep III	0.9975	2.6273	0.6996

vc1953	Rep I	1.1148	4.6110	0.3533
	Rep II	1.1299	4.5285	0.2258
	Rep III	0.9930	4.1123	0.1864
vc1449	Rep I	1.1366	1.7625	0.6026
	Rep II	0.9996	1.8812	0.7214
	Rep III	0.9714	1.7619	0.6492
lamB	Rep I	0.9987	0.3298	15.5598
	Rep II	0.9937	0.2134	13.1622
	Rep III	1.0032	0.2933	12.7289

Statistical analysis related to Fig. EV2C

ANOVA table	SS	DF	MS	F (DFn, DFd)	P value
Interaction	393.1	18	21.84	F (18, 60) = 254.3	P<0.0001
Row Factor	14.42	2	7.209	F (2, 60) = 83.94	P<0.0001
Column Factor	128.2	9	14.24	F (9, 60) = 165.8	P<0.0001
Residual	5.153	60	0.08588		

Normality test (Shapiro-Wilk)
Passed normality test (alpha=0.05)? Yes

Multiple comparisons
Number of families 10
Number of comparisons per family 2
Alpha 0.05

Dunnett's multiple comparisons test	Mean Diff.	95.00% CI of diff.	Below threshold?	Summary	Adjusted P Value
ptsG					
WT pCtrl vs. Δ*vcdRP* pCtrl	-0.4984	-0.65456 to 0.3421	Yes	***	0.0002
WT pCtrl vs. Δ*vcdRP* pVcdRP	0.8773	0.3352 to 1.419	Yes	****	<0.0001
treB					
WT pCtrl vs. Δ*vcdRP* pCtrl	-1.166	-1.289 to -1.043	Yes	****	<0.0001
WT pCtrl vs. Δ*vcdRP* pVcdRP	0.7422	0.6195 to 0.8648	Yes	****	<0.0001
nagE					
WT pCtrl vs. Δ*vcdRP* pCtrl	-0.9691	-1.238 to -0.7000	Yes	****	<0.0001
WT pCtrl vs. Δ*vcdRP* pVcdRP	0.7441	0.4071 to 1.013	Yes	***	0.0004
ptsH					
WT pCtrl vs. Δ*vcdRP* pCtrl	-0.6580	-0.8251 to -0.4909	Yes	****	<0.0001
WT pCtrl vs. Δ*vcdRP* pVcdRP	0.8433	0.6762 to 1.010	Yes	****	<0.0001
ptsI					
WT pCtrl vs. Δ*vcdRP* pCtrl	-0.9020	-0.9971 to -0.8070	Yes	****	<0.0001
WT pCtrl vs. Δ*vcdRP* pVcdRP	0.8172	0.7211 to 0.9122	Yes	****	<0.0001

vca0053					
WT pCtrl vs. Δ*vcdRP* pCtrl	-0.5003	-0.6937 to -0.3070	Yes	***	0.0006
WT pCtrl vs. Δ*vcdRP* pVcdRP	0.5431	0.3498 to 0.7365	Yes	***	0.0004
vc2761					
WT pCtrl vs. Δ*vcdRP* pCtrl	-1.483	-1.769 to -1.197	Yes	****	<0.0001
WT pCtrl vs. Δ*vcdRP* pVcdRP	0.3906	0.1047 to 0.6764	Yes	*	0.0140
vc1953					
WT pCtrl vs. Δ*vcdRP* pCtrl	-3.3380	-3.731 to -2.945	Yes	****	<0.0001
WT pCtrl vs. Δ*vcdRP* pVcdRP	0.8241	0.4313 to 1.217	Yes	**	0.0017
vc1449					
WT pCtrl vs. Δ*vcdRP* pCtrl	-0.7660	-0.9373 to -0.5947	Yes	****	<0.0001
WT pCtrl vs. Δ*vcdRP* pVcdRP	0.3781	0.2068 to 0.5494	Yes	**	0.013
lamB					
WT pCtrl vs. Δ*vcdRP* pCtrl	0.7197	0.1776 to 1.262	Yes	**	0.0074
WT pCtrl vs. Δ*vcdRP* pVcdRP	-12.82	-13.36 to -12.28	Yes	****	<0.0001
