# Supplementary material for: A dual‐function RNA balances carbon uptake and central metabolism in Vibrio cholerae
Source: EMBO J. 2021 Oct 6;40(24):e108542. doi: 10.15252/embj.2021108542 (PMC8672173; doi:10.15252/embj.2021108542)
Supplement: Supplementary file 3 — Source Data for Expanded View and Appendix [file EMBJ-40-e108542-s004.zip › EMBOJ-2021-108542R_SourceDataForFigureEV3B-J.pdf]

Source Data Fig. EV3

Data related to Fig. EV3B

Data refers to the sfGFP levels of *ptsG* translational reporter fusion corrected for autofluorescence, calculated as relative fold change w.r.t. pCtrl (set to 1)

Rel. *ptsG::*sfGFP levels [AU]	Rep I	Rep II	Rep III
pCtrl	1.0127	0.9713	1.0160
pVcdRP	0.3532	0.3494	0.3476
pVcdR	0.3078	0.3109	0.3100
pVcdP	1.1789	1.1900	1.1554
pVcdRP 256bp	0.3897	0.3946	0.4011
pVcdRP 156bp	0.2494	0.2531	0.2441
pVcdRP 87bp	0.4551	0.4666	0.4744
pVcdRP 71bp	1.2985	1.2604	1.2829
pVcdRP Δ4C	1.1210	1.1174	1.0887

Statistical analysis related to Fig. EV3B

ANOVA table	SS	DF	MS	F (DFn, DFd)	P value
Treatment (between columns)	4.338	8	0.5423	F (8, 18) = 2760	P<0.0001
Residual (within columns)	0.003537	18	0.0001965		
Total	4.342	26			

**Equal variance test (Brown-Forsythe)**	
F (DFn, DFd)	0.6607 (8, 18)
P value	0.7186
P value summary	ns
Are SDs significantly different (P<0.05)?	No

**Normality test (Shapiro-Wilk)**	
Passed normality test (alpha=0.05)?	Yes

**Multiple comparisons**	
Number of families	1
Number of comparisons per family	8
Alpha	0.05

Dunnett's multiple comparisons test	Mean Diff.	95.00% CI of diff.	Below threshold?	Summary	Adjusted P Value
pCtrl vs. pVcdRP	0.6499	0.6163 to 0.6835	Yes	****	<0.0001
pCtrl vs. pVcdR	0.6904	0.6568 to 0.7240	Yes	****	<0.0001
pCtrl vs. pVcdP	-0.1748	-0.2084 to -0.1412	Yes	****	<0.0001
pCtrl vs. pVcdRP 256bp	0.6049	0.5713 to 0.6385	Yes	****	<0.0001
pCtrl vs. pVcdRP 156bp	0.7512	0.7176 to 0.7848	Yes	****	<0.0001
pCtrl vs. pVcdRP 87bp	0.5346	0.5010 to 0.5682	Yes	****	<0.0001
pCtrl vs. pVcdRP 71bp	-0.2806	-0.3142 to -0.2470	Yes	****	<0.0001
pCtrl vs. pVcdRP Δ4C	-0.1090	-0.1426 to -0.07542	Yes	****	<0.0001

Data related to Fig. EV3C

Data refers to the sfGFP levels of *treB* translational reporter fusion corrected for autofluorescence, calculated as relative fold change w.r.t. pCtrl (set to 1)

Rel. *treB* sfGFP levels [AU]	Rep I	Rep II	Rep III
pCtrl	1.022028	1.013426	0.964546
pVcdRP	0.061673	0.062215	0.065466
pVcdR	0.062851	0.061313	0.064934
pVcdP	1.155928	1.15744	1.156441
pVcdRP 256bp	0.127753	0.122905	0.123515
pVcdRP 156bp	0.109938	0.114007	0.113984
pVcdRP 87bp	0.082729	0.080357	0.080282
pVcdRP 71bp	1.250243	1.265462	1.199776
pVcdRP Δ4C	1.287292	1.29319	1.300422

Statistical analysis related to Fig. EV3C

**ANOVA table**	**SS**	**DF**	**MS**	**F (DFn, DFd)**	**P value**
Treatment (between columns)	7.980	8	0.9975	F (8, 18) = 4064	P<0.0001
Residual (within columns)	0.004418	18	0.0002455		
Total	7.984	26			

Equal variance test (Brown-Forsythe)

F (DFn, DFd)	1.395 (8, 18)
P value	0.2641
P value summary	ns
Are SDs significantly different (P<0.05)?	No

Normality test (Shapiro-Wilk)

Passed normality test (alpha=0.05)?	Yes

Multiple comparisons

Number of families	1
Number of comparisons per family	8
Alpha	0.05

Dunnett's multiple comparisons test	Mean Diff.	95.00% CI of diff.	Below threshold?	Summary	Adjusted P Value
pCtrl vs. pVcdRP	0.9369	0.8993 to 0.9744	Yes	****	<0.0001
pCtrl vs. pVcdR	0.937	0.8994 to 0.9745	Yes	****	<0.0001
pCtrl vs. pVcdP	-0.1566	-0.1942 to -0.1191	Yes	****	<0.0001
pCtrl vs. pVcdRP 256bp	0.8753	0.8377 to 0.9128	Yes	****	<0.0001
pCtrl vs. pVcdRP 156bp	0.8874	0.8498 to 0.9249	Yes	****	<0.0001
pCtrl vs. pVcdRP 87bp	0.9189	0.8813 to 0.9564	Yes	****	<0.0001
pCtrl vs. pVcdRP 71bp	-0.2385	-0.2760 to -0.2009	Yes	****	<0.0001
pCtrl vs. pVcdRP Δ4C	-0.2936	-0.3312 to -0.2561	Yes	****	<0.0001

Data related to Fig. EV3D

Data refers to the sfGFP levels of *nagE* translational reporter fusion corrected for autofluorescence, calculated as relative fold change w.r.t. pCtrl (set to 1)

Rel. *nagE* sfGFP levels [AU]	Rep I	Rep II	Rep III
pCtrl	1.012701	0.98038	1.006919
pVcdRP	0.137777	0.135835	0.138767
pVcdR	0.143428	0.142219	0.142638
pVcdP	1.098716	1.099919	1.093887
pVcdRP 256bp	0.038608	0.039116	0.039966
pVcdRP 156bp	0.034075	0.034461	0.034265
pVcdRP 87bp	0.079748	0.084113	0.085974
pVcdRP 71bp	1.098495	1.232228	1.261512
pVcdRP Δ4C	1.095507	1.11497	1.093527

Statistical analysis related to Fig. EV3D

**ANOVA table**	**SS**	**DF**	**MS**	**F (DFn, DFd)**	**P value**
Treatment (between columns)	6.914	8	0.8642	F (8, 18) = 970.6	P<0.0001
Residual (within columns)	0.01603	18	0.00008904		
Total	6.930	26			

Equal variance test (Brown-Forsythe)

F (DFn, DFd)	1.570 (8, 18)
P value	0.2028
P value summary	ns
Are SDs significantly different (P<0.05)?	No

Normality test (Shapiro-Wilk)

Passed normality test (alpha=0.05)?	Yes

Multiple comparisons

Number of families	1
Number of comparisons per family	8
Alpha	0.05

Dunnett's multiple comparisons test	Mean Diff.	95.00% CI of diff.	Below threshold?	Summary	Adjusted P Value
pCtrl vs. pVcdRP	0.8625	0.7910 to 0.9341	Yes	****	<0.0001
pCtrl vs. pVcdR	0.8572	0.7857 to 0.9288	Yes	****	<0.0001
pCtrl vs. pVcdP	-0.09751	-0.1690 to -0.02599	Yes	**	0.0053
pCtrl vs. pVcdRP 256bp	0.9608	0.8893 to 1.032	Yes	****	<0.0001
pCtrl vs. pVcdRP 156bp	0.9657	0.8942 to 1.037	Yes	****	<0.0001
pCtrl vs. pVcdRP 87bp	0.9167	0.8452 to 0.9882	Yes	****	<0.0001
pCtrl vs. pVcdRP 71bp	-0.1974	-0.2689 to -0.1259	Yes	****	<0.0001
pCtrl vs. pVcdRP Δ4C	-0.1013	-0.1729 to -0.02982	Yes	**	0.0038

Data related to Fig. EV3E

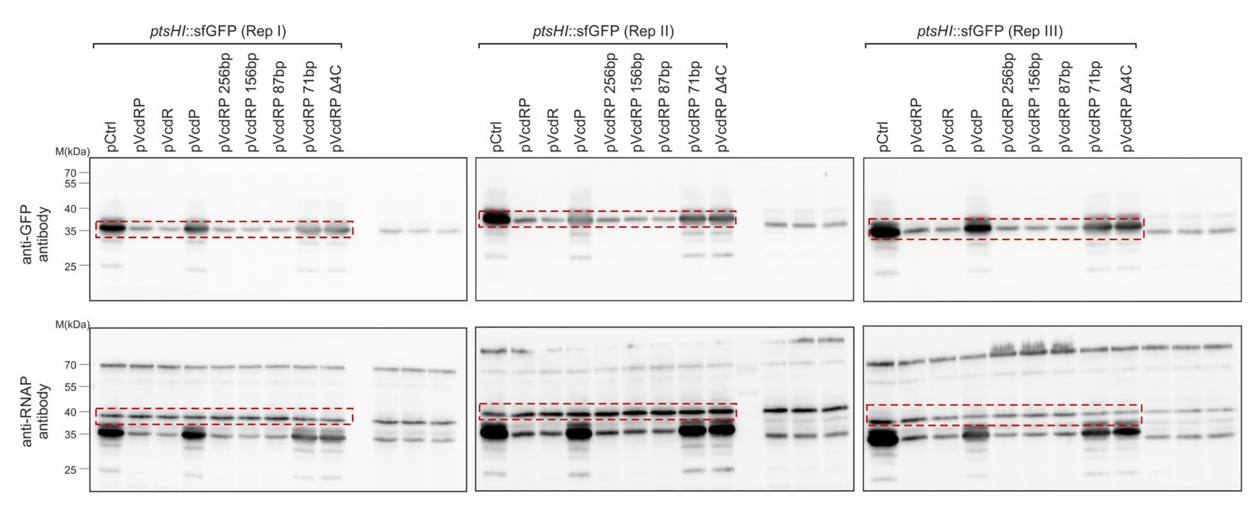

Data refers to the quantification of sfGFP levels from a Western blot analysis, normalized to its corresponding loading control detected with anti-RNAP antibody, calculated as relative fold change w.r.t. pCtrl (set to 1)

Rel. *ptsHI* sfGFP levels [AU]	Rep I	Rep II	Rep III
pCtrl	1.000000	1.000000	1.000000
pVcdRP	0.224673	0.24147	0.272409
pVcdR	0.111391	0.112913	0.153847
pVcdP	0.64679	0.758755	0.773731
pVcdRP 256bp	0.191136	0.162022	0.228733
pVcdRP 156bp	0.160362	0.113125	0.170758
pVcdRP 87bp	0.110031	0.106954	0.195689
pVcdRP 71bp	0.809639	0.67986	0.657157
pVcdRP Δ4C	0.80928	0.756538	0.813152

Statistical analysis related to Fig. EV3E

**ANOVA table**	**SS**	**DF**	**MS**	**F (DFn, DFd)**	**P value**
Treatment (between columns)	2.903	8	0.3629	F (8, 18) = 178.0	P<0.0001
Residual (within columns)	0.03669	18	0.002038		
Total	2.940	26			

Equal variance test (Brown-Forsythe)

F (DFn, DFd)	0.4688 (8, 18)
P value	0.8623
P value summary	ns
Are SDs significantly different (P<0.05)?	No

Normality test (Shapiro-Wilk)

Passed normality test (alpha=0.05)?	Yes

Multiple comparisons

Number of families	1
Number of comparisons per family	8
Alpha	0.05

Dunnett's multiple comparisons test	Mean Diff.	95.00% CI of diff.	Below threshold?	Summary	Adjusted P Value
pCtrl vs. pVcdRP	0.7538	0.6456 to 0.8620	Yes	****	<0.0001
pCtrl vs. pVcdR	0.8739	0.7657 to 0.9822	Yes	****	<0.0001
pCtrl vs. pVcdP	0.2736	0.1654 to 0.3818	Yes	****	<0.0001
pCtrl vs. pVcdRP 256bp	0.806	0.6978 to 0.9142	Yes	****	<0.0001
pCtrl vs. pVcdRP 156bp	0.8519	0.7437 to 0.9601	Yes	****	<0.0001
pCtrl vs. pVcdRP 87bp	0.8624	0.7542 to 0.9707	Yes	****	<0.0001
pCtrl vs. pVcdRP 71bp	0.2844	0.1762 to 0.3927	Yes	****	<0.0001
pCtrl vs. pVcdRP Δ4C	0.207	0.09880 to 0.3152	Yes	***	0.0002

Data related to Fig. EV3F

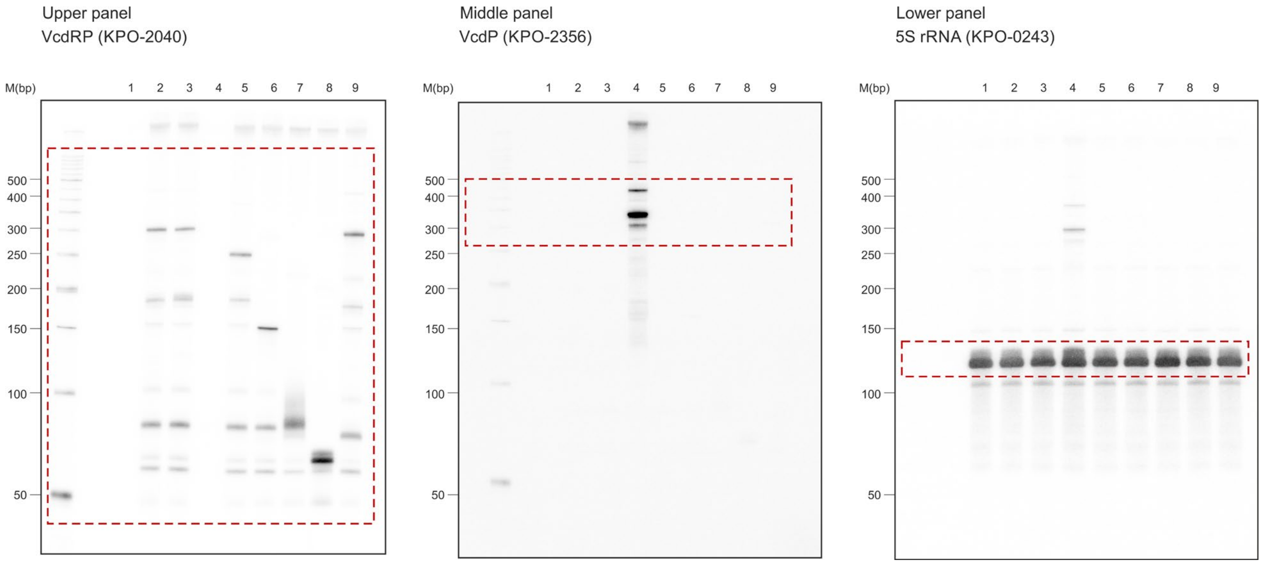

Data related to Fig. EV3G

Upper panel
VcdRP (KPO-2040)

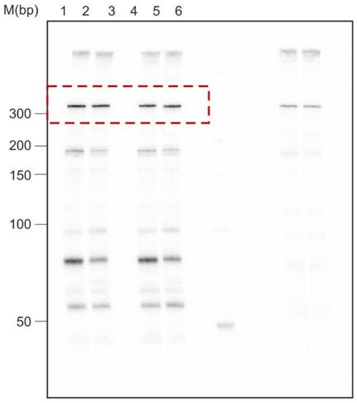

Lower panel
5S rRNA (KPO-0243)

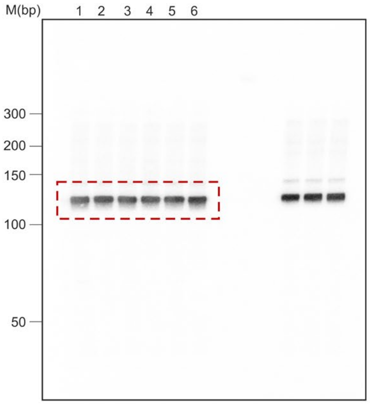

Data related to Fig. EV3H

Upper panel
VcdRP (KPO-2040)

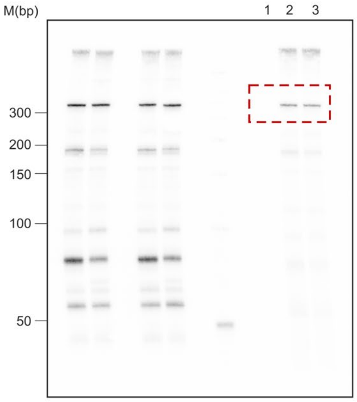

Lower panel
5S rRNA (KPO-0243)

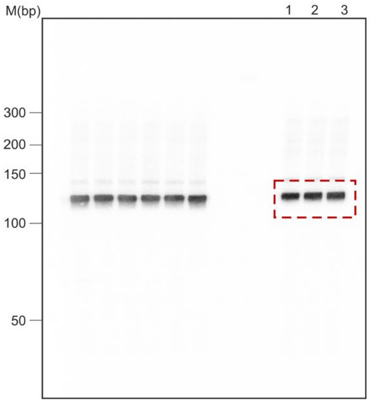

Data related to Fig. EV3I

Data refers to the sfGFP levels of *ptsG, treB and nagE* translational reporter fusions expressed in *E. coli* cells lacking *hfq,* corrected for autofluorescence, calculated as relative fold change w.r.t. pCtrl (set to 1). For the translational reporter of *ptsHI*, the measured fluorescence was below background autofluorescence. Hence, protein samples were collected and analyzed on a Western blot using anti-GFP antibody as shown below. Thus, the data refers to the quantification of sfGFP levels, normalized to its corresponding loading control detected with anti-RNAP antibody, calculated as relative fold change w.r.t. pCtrl (set to 1)

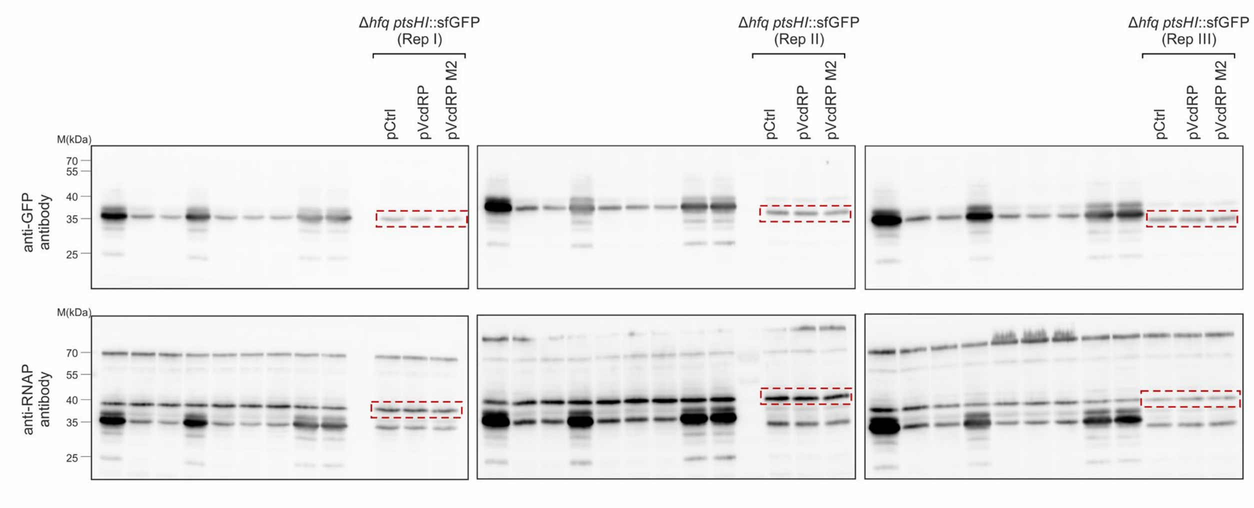

Rel. sfGFP levels [AU]	*Δhfq* pCtrl	*Δhfq* pVcdRP	*Δhfq* pVcdRP M2
ptsG	0.9720	0.9765	0.9758
	1.0215	0.9501	0.9811
	1.0065	0.9881	0.9914
treB	1.0221	1.0462	0.9317
	1.1139	1.1355	1.0996
	1.1573	0.9733	1.0790
nagE	1.0090	0.9197	0.9050
	1.0737	0.9773	0.9011
	0.9173	0.8545	0.9042
ptsHI	1.0000	1.1573	1.0075
	1.0000	0.9727	1.0363
	1.0000	1.0419	0.9714

Statistical analysis related to Fig. EV3I

**ANOVA table**	**SS**	**DF**	**MS**	**F (DFn, DFd)**	**P value**
Interaction	0.01180	6	0.003133	F (6, 24) = 0.9272	P=0.4933
Row factor	0.01091	2	0.005457	F (2, 24) = 1.615	P=0.2197
Column factor	0.07268	3	0.02423	F (3, 24) = 7.171	P=0.0013
Residual	0.08109	24	0.003379		

Normality test (Shapiro-Wilk)
Passed normality test (alpha=0.05)? Yes

Multiple comparisons
Number of families 4
Number of comparisons per family 2
Alpha 0.05

Dunnett's multiple comparisons test	Mean Diff.	95.00% CI of diff.	Below threshold?	Summary	Adjusted P Value
ptsG					
Δhfq pCtrl vs. *Δhfq* pVcdRP	0.02844	-0.08307 to 0.1399	No	ns	0.7755
Δhfq pCtrl vs. *Δhfq* pVcdRP M2	0.0172	-0.09430 to 0.1287	No	ns	0.9092
treB					
Δhfq pCtrl vs. *Δhfq* pVcdRP	0.04608	-0.06543 to 0.1576	No	ns	0.5304
Δhfq pCtrl vs. *Δhfq* pVcdRP M2	0.06099	-0.05052 to 0.1725	No	ns	0.3473
nagE					
Δhfq pCtrl vs. *Δhfq* pVcdRP	0.0828	-0.02870 to 0.1943	No	ns	0.1635
Δhfq pCtrl vs. *Δhfq* pVcdRP M2	0.09657	-0.01493 to 0.2081	No	ns	0.0949
ptsHI					
Δhfq pCtrl vs. *Δhfq* pVcdRP	-0.05732	-0.1688 to 0.05419	No	ns	0.3885
Δhfq pCtrl vs. *Δhfq* pVcdRP M2	-0.005064	-0.1166 to 0.1064	No	ns	0.9917

Data related to Fig. EV3J

Data refers to the absorbance measured at OD_{600}

αMG stress	Ara induction		pCtrl	pVcdRP	pVcdR	pVcdP
-	-	Rep I	0.3104	0.3120	0.3366	0.3166
		Rep II	0.3185	0.3138	0.3297	0.3396
		Rep III	0.3211	0.3165	0.3275	0.3530
+	-	Rep I	0.1627	0.1783	0.1841	0.1736
		Rep II	0.1677	0.1777	0.1817	0.1724
		Rep III	0.1664	0.1743	0.1823	0.1730
-	+	Rep I	0.1673	0.2770	0.2812	0.1858
		Rep II	0.2078	0.2746	0.2773	0.1930
		Rep III	0.1760	0.2738	0.2594	0.1903
+	+	Rep I	0.3204	0.3456	0.3398	0.3425
		Rep II	0.3609	0.3633	0.3668	0.3771
		Rep III	0.3500	0.3714	0.3469	0.3815

Statistical analysis related to Fig. EV3J

**ANOVA table**	**SS**	**DF**	**MS**	**F (DFn, DFd)**	**P value**
Interaction	0.01746	9	0.001940	F (9, 32) = 13.60	P<0.0001
Row Factor	0.007871	3	0.002624	F (3, 32) = 18.39	P<0.0001
Column Factor	0.2518	3	0.08393	F (3, 32) = 588.4	P<0.0001
Residual	0.004564	32	0.0001426		

Equal variance test (Brown-Forsythe)

F (DFn, DFd)	20.77 (11, 36)
P value	<0.0001
P value summary	****
Are SDs significantly different (P<0.05)?	Yes

Normality test (Shapiro-Wilk)

Passed normality test (alpha=0.05)?	Yes

Multiple comparisons

Number of families	4
Number of comparisons per family	3
Alpha	0.05

Dunnett's multiple comparisons test	Mean Diff.	95.00% CI of diff.	Below threshold?	Summary	Adjusted P Value
αMG / arabinose					
- / -					
pCtrl vs. pVcdRP	0.002567	-0.02148 to 0.02661	No	ns	0.9874
pCtrl vs. pVcdR	-0.01460	-0.03864 to 0.009445	No	ns	0.3229
pCtrl vs. pVcdP	-0.01973	-0.04378 to 0.004311	No	ns	0.1267
+ / -					
pCtrl vs. pVcdRP	-0.01117	-0.03521 to 0.01288	No	ns	0.5315
pCtrl vs. pVcdR	-0.01710	-0.04114 to 0.006945	No	ns	0.2100
pCtrl vs. pVcdP	-0.007400	-0.03144 to 0.01664	No	ns	0.7897
- / +					
pCtrl vs. pVcdRP	-0.01633	-0.04038 to 0.007711	No	ns	0.2410
pCtrl vs. pVcdR	-0.007400	-0.03144 to 0.01664	No	ns	0.7897
pCtrl vs. pVcdP	-0.02327	-0.04731 to 0.0007780	No	ns	0.0597
+ / +					
pCtrl vs. pVcdRP	-0.09143	-0.1155 to -0.06739	Yes	****	<0.0001
pCtrl vs. pVcdR	-0.08893	-0.1130 to -0.06489	Yes	****	<0.0001
pCtrl vs. pVcdP	-0.006000	-0.03004 to 0.01804	No	ns	0.8717
