# Supplementary material for: A dual‐function RNA balances carbon uptake and central metabolism in Vibrio cholerae
Source: EMBO J. 2021 Oct 6;40(24):e108542. doi: 10.15252/embj.2021108542 (PMC8672173; doi:10.15252/embj.2021108542)

Source Data Fig. EV4

Data related to Fig. EV4A

Data refers to the sfGFP levels of *vca0053* translational reporter fusion corrected for autofluorescence, calculated as relative fold change w.r.t. pCtrl (set to 1)

Rel. sfGFP levels [AU]	Rep I	Rep II	Rep III
pCtrl	1.0921	0.9939	0.9940
pVcdRP	1.0729	1.0646	1.1579
pVcdR	1.1270	1.1188	1.0101
pVcdP	1.0334	1.1306	1.0348
pVcdP-SPA	1.1253	1.1234	1.1389

Statistical analysis related to Fig. EV4A

ANOVA table	SS	DF	MS	F (DFn, DFd)	P value
Treatment (between columns)	0.01744	4	0.004360	F (4, 10) = 1.639	P=0.2397
Residual (within columns)	0.02661	10	0.002661		
Total	0.04404	14			

**Equal variance test (Brown-Forsythe)**	
F (DFn, DFd)	0.2139 (4, 10)
P value	0.9248
P value summary	ns
Are SDs significantly different (P<0.05)?	No

**Normality test (Shapiro-Wilk)**	
Passed normality test (alpha=0.05)?	Yes

**Multiple comparisons**	
Number of families	1
Number of comparisons per family	4
Alpha	0.05

Dunnett's multiple comparisons test	Mean Diff.	95.00% CI of diff.	Below threshold?	Summary	Adjusted P Value
pCtrl vs. pVcdRP	-0.07181	-0.1936 to 0.04992	No	ns	0.3151
pCtrl vs. pVcdR	-0.05864	-0.1804 to 0.06309	No	ns	0.4744
pCtrl vs. pVcdP	-0.03960	-0.1613 to 0.08214	No	ns	0.7561
pCtrl vs. pVcdP-SPA	-0.1025	-0.2242 to 0.01924	No	ns	0.1048

Data related to Fig. EV4B

Data refers to the mKate2 levels of *vca0053* transcriptional reporter fusion corrected for autofluorescence, calculated as relative fold change w.r.t. pCtrl (set to 1)

Rel. mKate2 levels [AU]	Rep I	Rep II	Rep III
pCtrl	0.9640	1.1175	0.9185
pVcdRP	0.5887	0.6788	0.6670
pVcdR	1.0401	0.9243	0.8685
pVcdP	0.3125	0.2756	0.2354
pVcdP-SPA	0.3594	0.3109	0.3210

Statistical analysis related to Fig. EV4B

ANOVA table	SS	DF	MS	F (DFn, DFd)	P value
Treatment (between columns)	1.355	4	0.3387	F (4, 10) = 73.39	P<0.0001
Residual (within columns)	0.04616	10	0.004616		
Total	1.401	14			

Equal variance test (Brown-Forsythe)

F (DFn, DFd)	0.5684 (4, 10)
P value	0.6916
P value summary	ns
Are SDs significantly different (P<0.05)?	No

Normality test (Shapiro-Wilk)

Passed normality test (alpha=0.05)?	Yes

Multiple comparisons

Number of families	1
Number of comparisons per family	4
Alpha	0.05

Dunnett's multiple comparisons test	Mean Diff.	95.00% CI of diff.	Below threshold?	Summary	Adjusted P Value
pCtrl vs. pVcdRP	0.3552	0.1948 to 0.5155	Yes	***	0.0003
pCtrl vs. pVcdR	0.05570	-0.1046 to 0.2160	No	ns	0.7161
pCtrl vs. pVcdP	0.7255	0.5652 to 0.8858	Yes	****	<0.0001
pCtrl vs. pVcdP-SPA	0.6696	0.5092 to 0.8299	Yes	****	<0.0001

Data related to Fig. EV4C

Upper panel
Anti-Flag antibody

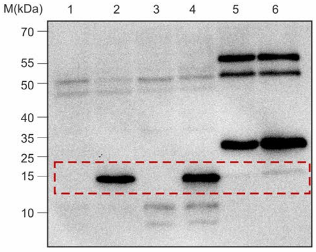

Middle panel
Anti-HA antibody

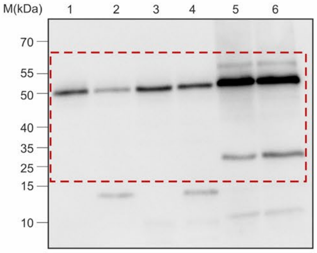

Lower panel
Anti-RNAP antibody

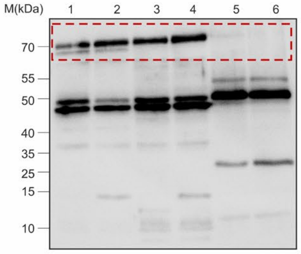

Data related to Fig. EV4D

Upper panel
Anti-His antibody

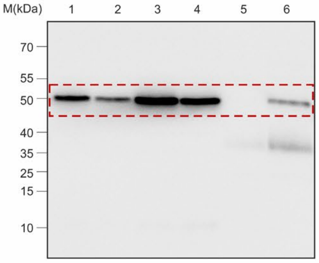

Middle panel
Anti-Flag antibody

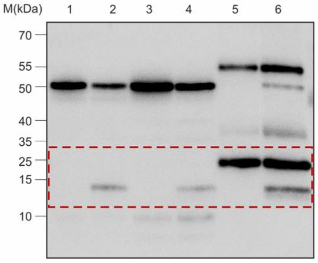

Lower panel
Anti-RNAP antibody

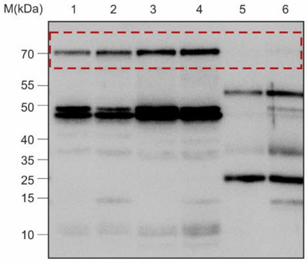

Data related to Fig. EV4E

Upper panel
Anti-Flag antibody

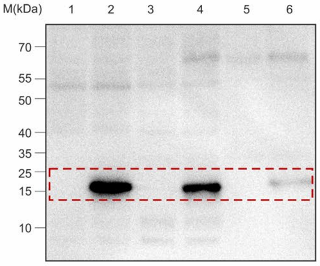

Middle panel
Anti-His antibody

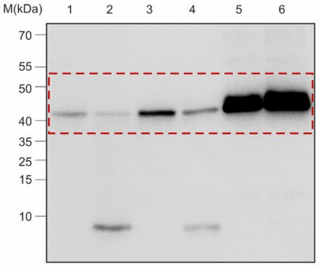

Lower panel
Anti-RNAP antibody

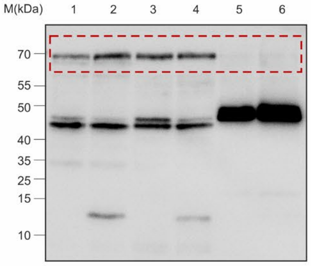

Data related to Fig. EV4F

Upper panel
Anti-HA antibody

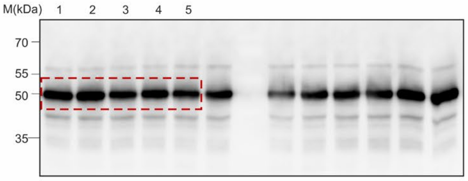

Lower panel
Anti-RNAP antibody

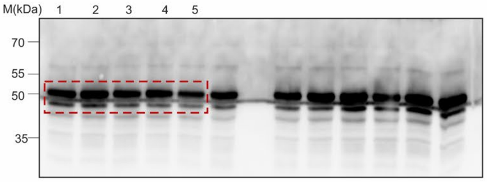

Supplement: Supplementary file 3 — Source Data for Expanded View and Appendix [file EMBJ-40-e108542-s004.zip › EMBOJ-2021-108542R_SourceDataForFigureEV4A-F.pdf]
