# Supplementary material for: A dual‐function RNA balances carbon uptake and central metabolism in Vibrio cholerae
Source: EMBO J. 2021 Oct 6;40(24):e108542. doi: 10.15252/embj.2021108542 (PMC8672173; doi:10.15252/embj.2021108542)
Supplement: Supplementary file 3 — Source Data for Expanded View and Appendix [file EMBJ-40-e108542-s004.zip › EMBOJ-2021-108542R_SourceDataForFigureEV5.pdf]

Source data Fig. EV5

Data refers to the normalized transcript levels of *ctxA* gene (w.r.t. the housekeeping gene *recA*). The mRNA levels are calculated as relative fold changes w.r.t. the levels of WT (-) set to 1.

(-) refers to treatment without and (+) refers to treatment with 5mM cAMP.

Rel. mRNA transcript levels	*ctxA*			*ctxB*		
WT (-)	0.998746	0.993657	1.003215	0.935226	0.960759	1.129281
WT (+)	0.547318	0.453012	0.530582	0.402792	0.392087	0.326905
Δ*cyaA*(-)	5.068698	5.034681	4.867989	6.038551	6.245325	5.963767
Δ*cyaA*(+)	1.809208	1.725906	1.725906	2.15397	2.054794	2.242779

Statistical analysis related to Fig. EV5

ANOVA table	SS	DF	MS	F (DFn, DFd)	P value
Interaction	1.357	3	0.4524	F (3, 16) = 61.01	P<0.0001
Row Factor	94.27	3	31.42	F (3, 16) = 4237	P<0.0001
Column Factor	0.6961	1	0.6961	F (1, 16) = 93.87	P<0.0001
Residual	0.1186	16	0.007416		

Normality test (Shapiro-Wilk)
Passed normality test (alpha=0.05)? Yes

Multiple comparisons
Number of families 2
Number of comparisons per family 6
Alpha 0.05

Tukey's multiple comparisons test	Mean Diff.	95.00% CI of diff.	Below threshold?	Summary	Adjusted P Value
ctxA					
WT (-) vs. WT (+)	0.4882	0.2871 to 0.6894	Yes	****	<0.0001
WT (-) vs. Δ*cyaA*(-)	-3.992	-4.193 to -3.791	Yes	****	<0.0001
WT (-) vs. Δ*cyaA*(+)	-0.7551	-0.9563 to -0.5540	Yes	****	<0.0001
WT (+) vs. Δ*cyaA*(-)	-4.48	-4.681 to -4.279	Yes	****	<0.0001
WT (+) vs. Δ*cyaA*(+)	-1.243	-1.445 to -1.042	Yes	****	<0.0001
Δ*cyaA*(-) vs. Δ*cyaA*(+)	3.237	3.036 to 3.438	Yes	****	<0.0001
ctxB					
WT (-) vs. WT (+)	0.6345	0.4333 to 0.8357	Yes	****	<0.0001
WT (-) vs. Δ*cyaA*(-)	-5.074	-5.275 to -4.873	Yes	****	<0.0001
WT (-) vs. Δ*cyaA*(+)	-1.142	-1.343 to -0.9409	Yes	****	<0.0001
WT (+) vs. Δ*cyaA*(-)	-5.709	-5.910 to -5.507	Yes	****	<0.0001
WT (+) vs. Δ*cyaA*(+)	-1.777	-1.978 to -1.575	Yes	****	<0.0001
Δ*cyaA*(-) vs. Δ*cyaA*(+)	3.932	3.731 to 4.133	Yes	****	<0.0001
