# Supplementary material for: A dual‐function RNA balances carbon uptake and central metabolism in Vibrio cholerae
Source: EMBO J. 2021 Oct 6;40(24):e108542. doi: 10.15252/embj.2021108542 (PMC8672173; doi:10.15252/embj.2021108542)

Source Data Fig. 1

Fig. 1A

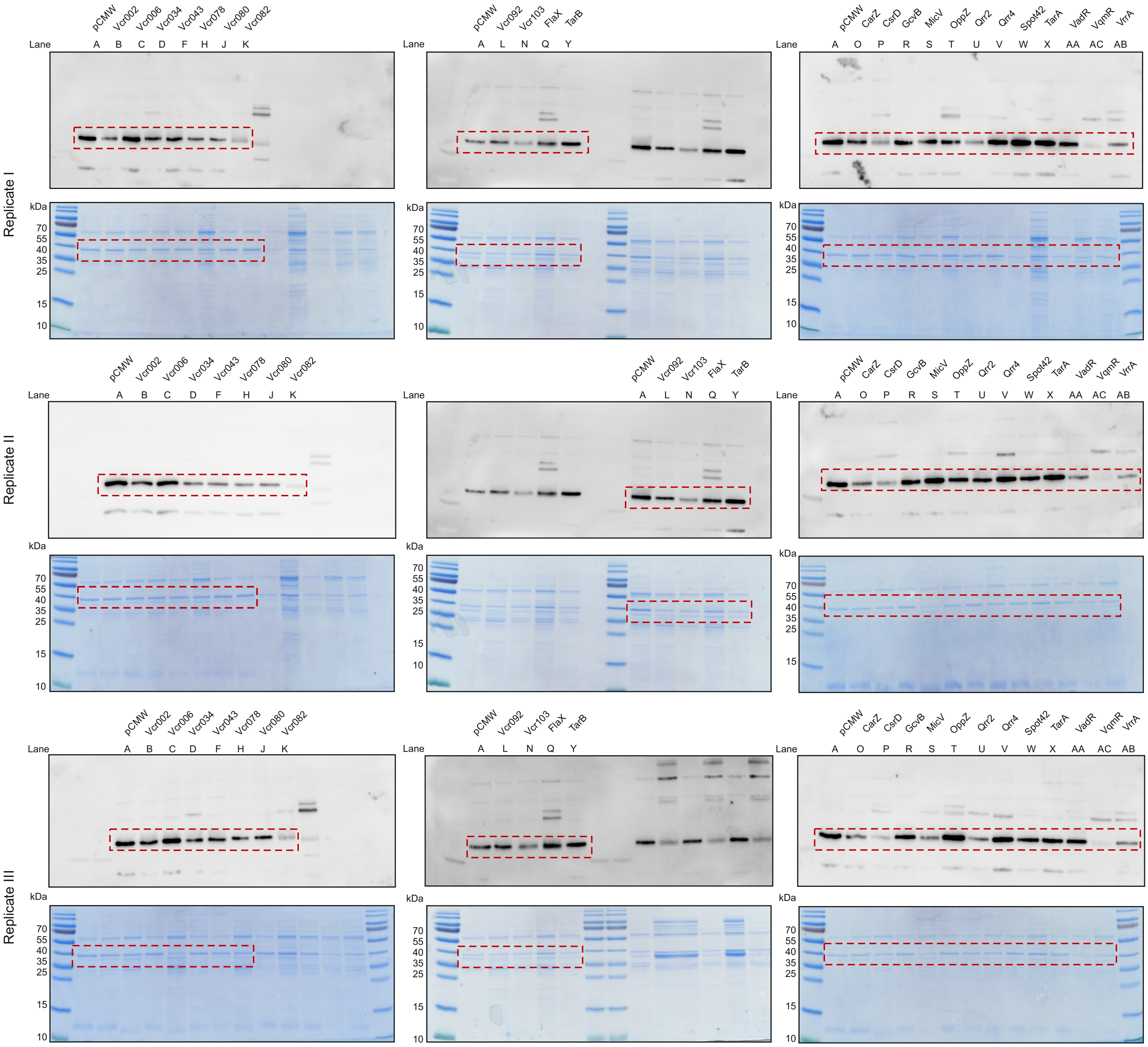

Data related to Fig. 1A

Data refers to the CTX band intensities normalized to the corresponding Coomassie-stained loading control, calculated as relative fold-repression w.r.t. pCMW

Lane	A	B	C	D	E	F	G	H	I	J	K	L	M	N	O
Sample	pCMW	Vcr002	Vcr006	Vcr034	Vcr036	Vcr043	Vcr065	Vcr078	Vcr079	Vcr080	Vcr082	Vcr092	Vcr098	Vcr103	CarZ
Rep I	0.974125	1.39913	0.693063	0.672545	1.137773	0.546278	1.505004	0.764649	1.333351	0.858001	5.329638	0.683653	1.072099	1.402072	1.694283
Rep II	1.214137	1.639963	0.930613	0.631034	1.018818	0.530631	2.335248	0.630672	1.925892	1.104021	5.817674	0.532382	1.347237	1.838675	2.150121
Rep III	0.994317	1.355895	0.968148	0.595107	1.078935	0.591926	3.135681	0.600078	1.871538	1.05639	4.268032	0.603653	1.1788	2.548875	1.279607

Lane	P	Q	R	S	T	U	V	W	X	Y	Z	AA	AB	AC
Sample	CsrD	FlaX	GcvB	MicV	OppZ	Qrr2	Qrr4	Spot42	TarA	TarB	TfoR	VadR	VrrA	VqmR
Rep I	2.480651	0.564812	0.718071	1.009734	0.713175	2.114791	0.869875	0.698724	0.708235	0.545941	1.967807	1.015868	1.791601	8.141996
Rep II	2.498438	0.55605	0.615207	1.157153	0.593511	1.444899	1.070767	0.624274	0.607485	0.585038	1.873466	1.528374	2.666027	8.704735
Rep III	2.401998	0.640746	0.671326	1.174688	0.520383	2.700586	1.157823	0.610907	0.578637	0.548276	1.551181	2.418263	2.104864	7.507508

Statistical analysis related to Fig. 1A

ANOVA summary

F	59.50
P value	<0.0001
P value summary	****
Significant diff. among means (P<0.005)?	Yes

Equal variance test (Brown-Forsythe)

F (Dfn, Dfd)	1.729 (28, 58)
P value	0.0397
P value summary	*
Are SDs significantly different (P<0.05)?	Yes

Normality test (Shapiro-Wilk)

Passed normality test (alpha =0.05)?	Yes

Multiple comparisons

Number of families	1
Number of comparisons per family	28
Alpha	0.05

Dunnett's multiple comparisons test	Mean Diff.	95.00% CI of diff.	Below threshold?	Summary	Adjusted P Value	A-?
pCMW vs. Vcr002	-0.4041	-1.296 to 0.4879	No	ns	0.8973	B
pCMW vs. Vcr006	0.1969	-0.6951 to 1.089	No	ns	0.999	C
pCMW vs. Vcr034	0.428	-0.4641 to 1.320	No	ns	0.8512	D
pCMW vs. Vcr036	-0.01765	-0.9097 to 0.8744	No	ns	>0.9999	E
pCMW vs. Vcr043	0.5046	-0.3875 to 1.397	No	ns	0.6613	F
pCMW vs. Vcr065	-1.264	-2.156 to -0.3724	Yes	**	0.0011	G
pCMW vs. Vcr078	0.3957	-0.4963 to 1.288	No	ns	0.9114	H
pCMW vs. Vcr079	-0.6494	-1.541 to 0.2426	No	ns	0.312	I
pCMW vs. Vcr080	0.05472	-0.8373 to 0.9468	No	ns	0.9997	J
pCMW vs. Vcr082	-4.078	-4.970 to -3.186	Yes	****	<0.0001	K
pCMW vs. Vcr092	0.4543	-0.4377 to 1.346	No	ns	0.7912	L
pCMW vs. Vcr098	-0.1385	-1.031 to 0.7535	No	ns	0.9994	M
pCMW vs. Vcr103	-0.869	-1.761 to 0.02303	No	ns	0.0611	N
pCMW vs. CarZ	-0.6471	-1.539 to 0.2449	No	ns	0.3164	O
pCMW vs. CsrD	-1.4	-2.292 to -0.5075	Yes	***	0.0003	P
pCMW vs. FlaX	0.4737	-0.4184 to 1.366	No	ns	0.7429	Q
pCMW vs. GcvB	0.3927	-0.4994 to 1.285	No	ns	0.9163	R
pCMW vs. MicV	-0.053	-0.9450 to 0.8390	No	ns	0.9998	S
pCMW vs. OppZ	0.4518	-0.4402 to 1.344	No	ns	0.7971	T
pCMW vs. Qrr2	-1.026	-1.918 to -0.1339	Yes	*	0.0143	U
pCMW vs. Qrr4	0.02804	-0.8640 to 0.9201	No	ns	0.9999	V
pCMW vs. Spot42	0.4162	-0.4758 to 1.308	No	ns	0.8749	W
pCMW vs. TarA	0.4294	-0.4626 to 1.321	No	ns	0.8481	X
pCMW vs. TarB	0.5011	-0.3909 to 1.393	No	ns	0.6706	Y
pCMW vs. TfoR	-0.7366	-1.629 to 0.1554	No	ns	0.1739	Z
pCMW vs. VadR	-0.5933	-1.485 to 0.2987	No	ns	0.4324	AA
pCMW vs. VrrA	-1.127	-2.019 to -0.2346	Yes	**	0.0051	AB
pCMW vs. VqmR	-7.057	-7.949 to -6.165	Yes	****	<0.0001	AC

Fig. 1D

Upper panel
VcdRP (KPO-2040)

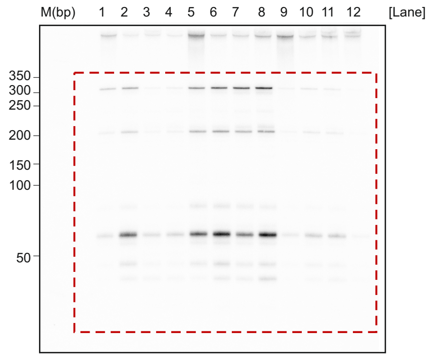

Lower panel
5S rRNA (KPO-0243)

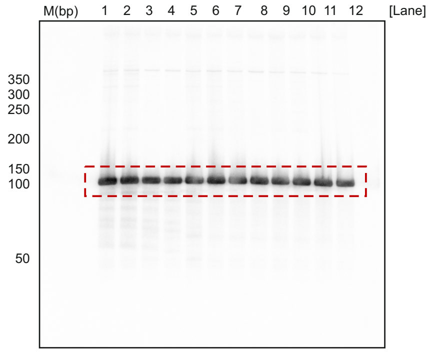

Fig. 1F

Upper panel
anti-FLAG

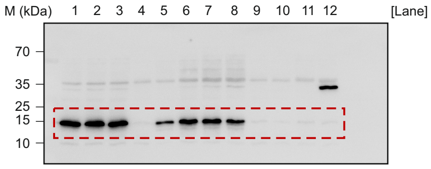

Lower panel
anti-RNAP

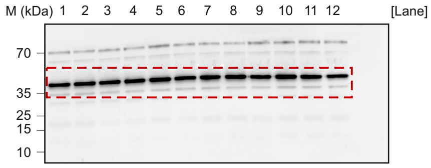

Supplement: Supplementary file 4 — Source Data for Figure 1 [file EMBJ-40-e108542-s006.pdf]
