# Supplementary material for: A dual‐function RNA balances carbon uptake and central metabolism in Vibrio cholerae
Source: EMBO J. 2021 Oct 6;40(24):e108542. doi: 10.15252/embj.2021108542 (PMC8672173; doi:10.15252/embj.2021108542)

Source Data Fig. 2

Data related to Fig. 2A

Upper panel
VcdRP (KPO-2040)

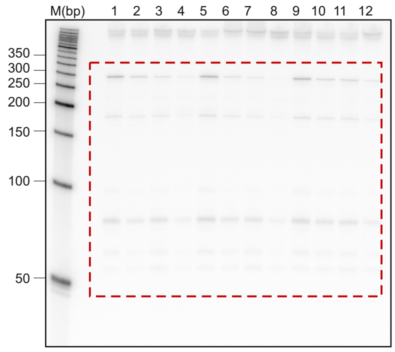

Middle panel
Spot42 (KPO-3726)

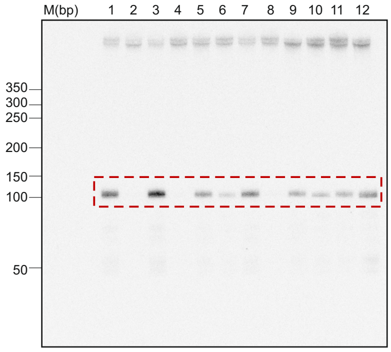

Lower panel
5S (KPO-0243)

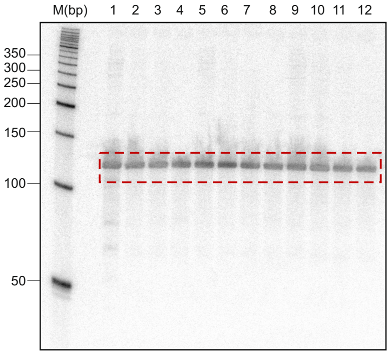

Data related to Fig. 2B

Data refers to the mKate2 levels for each reporter gene fusion corrected for autofluorescence, calculated as relative fold change w.r.t. WT (set to 1)

Rel. mKate2 levels [AU]	WT	Δ*crp*	Δ*cyaA*
Rep I	1.06869	3.332737	2.376526
Rep II	0.93131	2.685386	2.531491
Rep III	0.991557	2.747536	2.332966

Statistical analysis related to Fig. 2B

Normality test (Shapiro-Wilk)
Passed normality test (alpha =0.05)? Yes

Multiple comparisons test
Number of families 1
Number of comparisons per family 2
Alpha 0.05

Dunnett's multiple comparisons test	Mean Diff.	95.00% CI of diff.	Below threshold?	Summary	Adjusted P Value
WT vs. Δ*crp*	-1.925	-2.331 to -1.518	Yes	****	<0.0001
WT vs. Δ*cyaA*	-1.416	-1.823 to -1.010	Yes	****	<0.0001

Data related to Fig. 2C

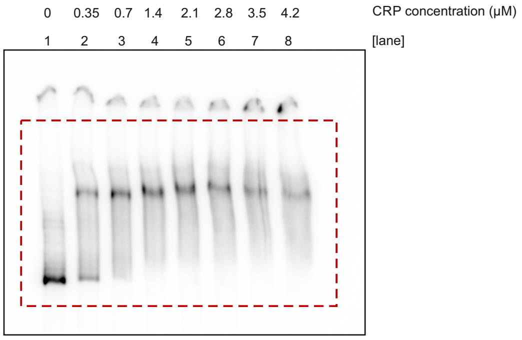

Supplement: Supplementary file 5 — Source Data for Figure 2 [file EMBJ-40-e108542-s002.pdf]
