# Supplementary figures and images for: A dual‐function RNA balances carbon uptake and central metabolism in Vibrio cholerae
Source: EMBO J. 2021 Oct 6;40(24):e108542. doi: 10.15252/embj.2021108542 (PMC8672173; doi:10.15252/embj.2021108542)

Source Data Fig. 3

Fig. 3B

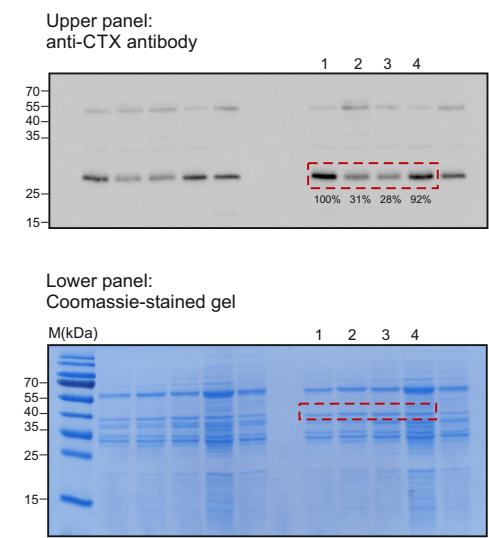

Supplement: Supplementary file 6 — Source Data for Figure 3 [file EMBJ-40-e108542-s010.pdf]
