# Supplementary material for: A dual‐function RNA balances carbon uptake and central metabolism in Vibrio cholerae
Source: EMBO J. 2021 Oct 6;40(24):e108542. doi: 10.15252/embj.2021108542 (PMC8672173; doi:10.15252/embj.2021108542)
Supplement: Supplementary file 7 — Source Data for Figure 4 [file EMBJ-40-e108542-s005.pdf]

Source Data Fig. 4

Data related to Fig. 4A-D

Data refers to the sfGFP levels for each reporter gene fusion corrected for autofluorescence, calculated as relative fold change w.r.t. pCtrl (set to 1)

Gene fusion	*treB*::sfGFP			*nagE*::sfGFP		
Rel. sfGFP levels [AU]	Rep I	Rep II	Rep III	Rep I	Rep II	Rep III
pCtrl	1.0220	1.0134	0.9645	1.0127	0.9804	1.0069
pVcdRP	0.0617	0.0622	0.0615	0.1378	0.1358	0.1388
pVcdR	0.0629	0.0613	0.0629	0.1434	0.1422	0.1426
pVcdP	1.1559	1.1574	1.1564	1.0987	1.0999	1.0939
pVcdRP Δ4C	1.2873	1.2932	1.3004	1.0955	1.1150	1.0935

Gene fusion	*ptsG*::sfGFP			*ptsHI*::sfGFP		
Rel. sfGFP levels [AU]	Rep I	Rep II	Rep III	Rep I	Rep II	Rep III
pCtrl	1.0056	0.9979	0.9965	1.0010	0.9911	1.0078
pVcdRP	0.4110	0.4056	0.4035	0.0462	0.0455	0.0470
pVcdR	0.3729	0.3776	0.3810	0.0481	0.0472	0.0474
pVcdP	1.1922	1.1710	1.1745	1.0557	1.0536	1.0454
pVcdRP Δ4C	1.0885	1.0998	1.1024	0.7990	0.8022	0.8031

Statistical analysis related to Fig. 4A-D

**ANOVA table**	**SS**	**DF**	**MS**	**F (DFn, DFd)**	**P value**
Interaction	0.4873	12	0.04060	F (12, 40) = 451.4	P<0.0001
Row Factor	11.87	4	2.968	F (4, 40) = 32976	P<0.0001
Column Factor	0.3749	3	0.1250	F (3, 40) = 1388	P<0.0001
Residual	0.003601	40	9.001e-005		

Normality test (Shapiro-Wilk)
Passed normality test (alpha=0.05)? Yes

Multiple comparisons
Number of families 4
Number of comparisons per family 10
Alpha 0.05

Tukey's multiple comparisons test	Mean Diff.	95.00% CI of diff.	Below threshold?	Summary	Adjusted P Value
treB::sfGFP					
pCtrl vs. pVcdRP	0.9382	0.9161 to 0.9603	Yes	****	<0.0001
pCtrl vs. pVcdR	0.9376	0.9155 to 0.9598	Yes	****	<0.0001
pCtrl vs. pVcdP	-0.1566	-0.1787 to -0.1345	Yes	****	<0.0001
pCtrl vs. pVcdRP Δ4C	-0.2936	-0.3158 to -0.2715	Yes	****	<0.0001
pVcdRP vs. pVcdR	-0.0005	-0.0227 to 0.0215	No	ns	>0.9999
pVcdRP vs. pVcdP	-1.095	-1.117 to -1.073	Yes	****	<0.0001
pVcdRP vs. pVcdRP Δ4C	-1.232	-1.254 to -1.210	Yes	****	<0.0001
pVcdR vs. pVcdP	-1.094	-1.116 to -1.072	Yes	****	<0.0001
pVcdR vs. pVcdRP Δ4C	-1.231	-1.253 to -1.209	Yes	****	<0.0001
pVcdP vs. pVcdRP Δ4C	-0.137	-0.1592 to -0.1149	Yes	****	<0.0001

nagE::sfGFP					
pCtrl vs. pVcdRP	0.8625	0.8404 to 0.8847	Yes	****	<0.0001
pCtrl vs. pVcdR	0.8572	0.8351 to 0.8794	Yes	****	<0.0001
pCtrl vs. pVcdP	-0.0975	-0.1196 to -0.0753	Yes	****	<0.0001
pCtrl vs. pVcdRP Δ4C	-0.1013	-0.1235 to -0.0792	Yes	****	<0.0001
pVcdRP vs. pVcdR	-0.0053	-0.0274 to 0.0168	No	ns	0.9587
pVcdRP vs. pVcdP	-0.96	-0.9822 to -0.9379	Yes	****	<0.0001
pVcdRP vs. pVcdRP Δ4C	-0.9639	-0.9860 to -0.9417	Yes	****	<0.0001
pVcdR vs. pVcdP	-0.9547	-0.9769 to -0.9326	Yes	****	<0.0001
pVcdR vs. pVcdRP Δ4C	-0.9586	-0.9807 to -0.9364	Yes	****	<0.0001
pVcdP vs. pVcdRP Δ4C	-0.0038	-0.0259 to 0.0183	No	ns	0.9875
ptsG::sfGFP					
pCtrl vs. pVcdRP	0.5933	0.5712 to 0.6154	Yes	****	<0.0001
pCtrl vs. pVcdR	0.6228	0.6007 to 0.6449	Yes	****	<0.0001
pCtrl vs. pVcdP	-0.1792	-0.2014 to -0.1571	Yes	****	<0.0001
pCtrl vs. pVcdRP Δ4C	-0.0969	-0.1190 to -0.0747	Yes	****	<0.0001
pVcdRP vs. pVcdR	0.0294	0.0073 to 0.0516	Yes	**	0.0041
pVcdRP vs. pVcdP	-0.7726	-0.7947 to -0.7504	Yes	****	<0.0001
pVcdRP vs. pVcdRP Δ4C	-0.6902	-0.7124 to -0.6681	Yes	****	<0.0001
pVcdR vs. pVcdP	-0.802	-0.8242 to -0.7799	Yes	****	<0.0001
pVcdR vs. pVcdRP Δ4C	-0.7197	-0.7418 to -0.6976	Yes	****	<0.0001
pVcdP vs. pVcdRP Δ4C	0.0823	0.0601 to 0.1044	Yes	****	<0.0001
ptsHI::sfGFP					
pCtrl vs. pVcdRP	0.9538	0.9317 to 0.9759	Yes	****	<0.0001
pCtrl vs. pVcdR	0.9524	0.9303 to 0.9746	Yes	****	<0.0001
pCtrl vs. pVcdP	-0.0515	-0.0737 to -0.0294	Yes	****	<0.0001
pCtrl vs. pVcdRP Δ4C	0.1986	0.1764 to 0.2207	Yes	****	<0.0001
pVcdRP vs. pVcdR	-0.0013	-0.0234 to 0.0207	No	ns	0.9998
pVcdRP vs. pVcdP	-1.005	-1.027 to -0.9832	Yes	****	<0.0001
pVcdRP vs. pVcdRP Δ4C	-0.7552	-0.7773 to -0.7331	Yes	****	<0.0001
pVcdR vs. pVcdP	-1.004	-1.026 to -0.9819	Yes	****	<0.0001
pVcdR vs. pVcdRP Δ4C	-0.7539	-0.7760 to -0.7318	Yes	****	<0.0001
pVcdP vs. pVcdRP Δ4C	0.2501	0.2280 to 0.2723	Yes	****	<0.0001

Data related to Fig. 4I-4K

Data refers to the sfGFP levels for each reporter gene fusion corrected for autofluorescence, calculated as relative fold change w.r.t. pCtrl (set to 1)

Gene fusion	*treB*::sfGFP			*treB M2**::sfGFP		
Rel. sfGFP levels [AU]	Rep I	Rep II	Rep III	Rep I	Rep II	Rep III
pCtrl	0.9989	1.0109	0.9902	0.9948	1.0207	0.9845
pVcdRP	0.0683	0.0693	0.0689	1.0951	1.1278	1.1284
pVcdR M2	0.6950	0.7437	0.7353	0.1109	0.1115	0.1147

Gene fusion	*ptsG*::sfGFP			*ptsG M2**::sfGFP		
Rel. sfGFP levels [AU]	Rep I	Rep II	Rep III	Rep I	Rep II	Rep III
pCtrl	0.9514	1.0345	1.0141	1.0033	0.9804	1.0163
pVcdRP	0.3559	0.3690	0.3708	1.1943	1.1841	1.2529
pVcdR M2	1.0749	1.0879	1.1421	0.7032	0.6726	0.7652

Gene fusion	*nagE*::sfGFP			*nagE M2**::sfGFP		
Rel. sfGFP levels [AU]	Rep I	Rep II	Rep III	Rep I	Rep II	Rep III
pCtrl	1.1539	0.7962	1.0499	0.9830	0.9709	1.0460
pVcdRP	0.1195	0.1154	0.1132	0.9194	0.9718	0.9526
pVcdR M2	1.0619	1.0366	1.0091	0.5367	0.6368	0.4955

Statistical analysis related to Fig. 4I-4K

**ANOVA table**	**SS**	**DF**	**MS**	**F (DFn, DFd)**	**P value**
Interaction	5.163	10	0.5163	F (10, 36) = 182.9	P<0.0001
Row Factor	1.331	2	0.6654	F (2, 36) = 235.7	P<0.0001
Column Factor	0.7303	5	0.1461	F (5, 36) = 51.73	P<0.0001
Residual	0.1016	36	0.002824		

Normality test (Shapiro-Wilk)
Passed normality test (alpha=0.05)? Yes

Multiple comparisons

Number of families 6
Number of comparisons per family 3
Alpha 0.05

Tukey's multiple comparisons test	Mean Diff.	95.00% CI of diff.	Below threshold?	Summary	Adjusted P Value
treB::sfGFP					
pCtrl vs. pVcdRP	0.9312	0.8213 to 1.041	Yes	****	<0.0001
pCtrl vs. pVcdR M2	0.2753	0.1655 to 0.3852	Yes	****	<0.0001
pVcdRP vs. pVcdR M2	-0.6558	-0.7657 to -0.5460	Yes	****	<0.0001
*treB M2**::sfGFP					
pCtrl vs. pVcdRP	-0.1171	-0.2269 to -0.007255	Yes	*	0.0343
pCtrl vs. pVcdR M2	0.8876	0.7778 to 0.9975	Yes	****	<0.0001
pVcdRP vs. pVcdR M2	1.0050	0.8949 to 1.115	Yes	****	<0.0001
ptsG::sfGFP					
pCtrl vs. pVcdRP	0.6347	0.5249 to 0.7446	Yes	****	<0.0001
pCtrl vs. pVcdR M2	-0.1016	-0.2115 to 0.008203	No	ns	0.0750
pVcdRP vs. pVcdR M2	-0.7364	-0.8462 to -0.6265	Yes	****	<0.0001
*ptsG M2**::sfGFP					
pCtrl vs. pVcdRP	-0.2104	-0.3203 to -0.1006	Yes	****	<0.0001
pCtrl vs. pVcdR M2	0.2863	0.1765 to 0.3962	Yes	****	<0.0001

pVcdRP vs. pVcdR M2	0.4968	0.3869 to 0.6066	Yes	****	<0.0001
nagE::sfGFP					
pCtrl vs. pVcdRP	0.8839	0.7741 to 0.9938	Yes	****	<0.0001
pCtrl vs. pVcdR M2	-0.0359	-0.1457 to 0.07397	No	ns	0.7110
pVcdRP vs. pVcdR M2	-0.9198	-1.030 to -0.8100	Yes	****	<0.0001
*nagE M2**::sfGFP					
pCtrl vs. pVcdRP	0.0521	-0.05778 to 0.1619	No	ns	0.4908
pCtrl vs. pVcdR M2	0.4437	0.3339 to 0.5535	Yes	****	<0.0001
pVcdRP vs. pVcdR M2	0.3916	0.2818 to 0.5015	Yes	****	<0.0001

Data related to Fig. 4L

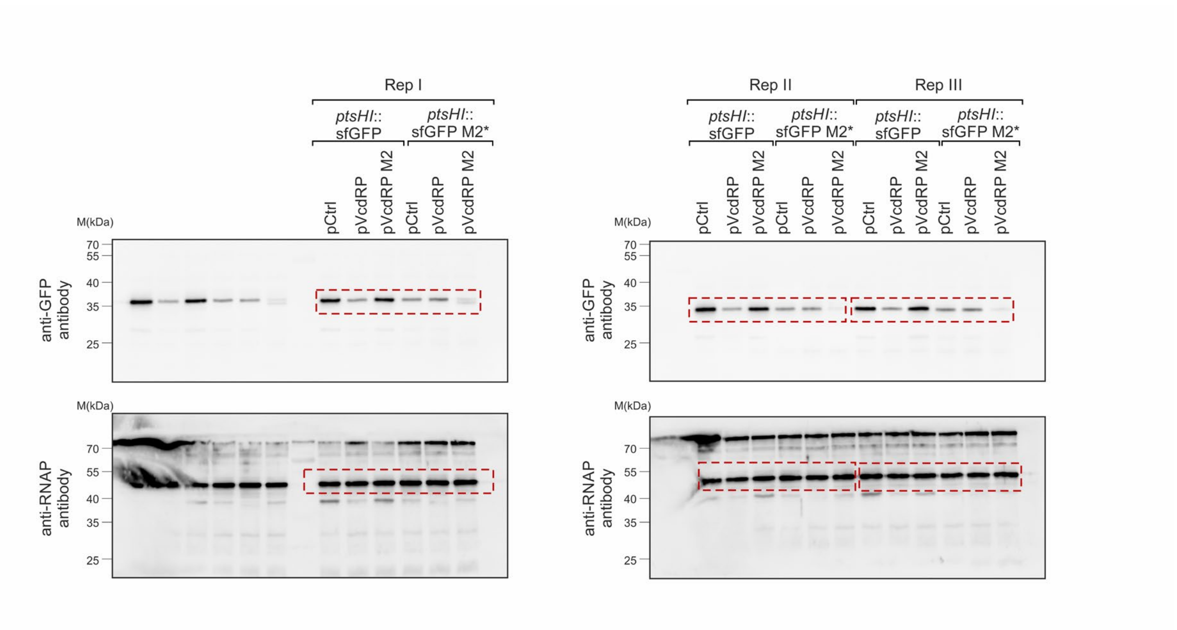

Data refers to the quantification of sfGFP levels from a Western blot analysis, normalized to its corresponding loading control detected with anti-RNAP antibody, calculated as relative fold change w.r.t. pCtrl (set to 1)

Gene fusion	*ptsHI::sfGFP*			*ptsHI M2**::sfGFP		
Rel. sfGFP levels [AU]	Rep I	Rep II	Rep III	Rep I	Rep II	Rep III
pCtrl	1.0000	1.0000	1.0000	0.9472	1.0874	1.0812
pVcdRP	0.1603	0.2476	0.1845	0.8373	1.0953	1.0586
pVcdR M2	0.8157	0.9387	0.8991	0.1587	0.1169	0.1022

Statistical analysis related to Fig. 4L

**ANOVA table**	**SS**	**DF**	**MS**	**F (DFn, DFd)**	**P value**
Interaction	1.821	2	0.9106	F (2, 12) = 167.7	P<0.0001
Row Factor	0.9017	2	0.4509	F (2, 12) = 83.01	P<0.0001
Column Factor	0.003168	1	0.003168	F (1, 12) = 0.5833	P=0.4598
Residual	0.06518	12	0.006432		

Normality test (Shapiro-Wilk)
Passed normality test (alpha=0.05)? Yes

Multiple comparisons

Number of families 2
Number of comparisons per family 3
Alpha 0.05

Tukey's multiple comparisons test	Mean Diff.	95.00% CI of diff.	Below threshold?	Summary	Adjusted P Value
ptsHI::sfGFP					
pCtrl vs. pVcdR	0.8025	0.6420 to 0.9631	Yes	****	<0.0001
pCtrl vs. pVcdR M2	0.1155	-0.04508 to 0.2760	No	ns	0.1758
pVcdR vs. pVcdR M2	-0.687	-0.8476 to -0.5265	Yes	****	<0.0001
ptsHI M2::sfGFP					
pCtrl vs. pVcdR	0.04156	-0.1190 to 0.2021	No	ns	0.7733
pCtrl vs. pVcdR M2	0.9127	0.7522 to 1.073	Yes	****	<0.0001
pVcdR vs. pVcdR M2	0.8711	0.7106 to 1.032	Yes	****	<0.0001
