# Supplementary material for: A dual‐function RNA balances carbon uptake and central metabolism in Vibrio cholerae
Source: EMBO J. 2021 Oct 6;40(24):e108542. doi: 10.15252/embj.2021108542 (PMC8672173; doi:10.15252/embj.2021108542)
Supplement: Supplementary file 8 — Source Data for Figure 5 [file EMBJ-40-e108542-s001.pdf]

Source Data Fig. 5

Data related to Fig. 5A

Upper panel
Anti-HA antibody

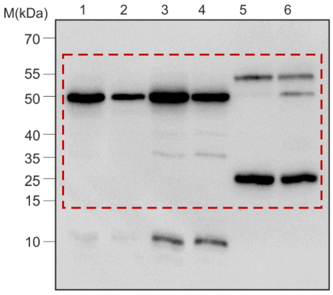

Middle panel
Anti-Flag antibody

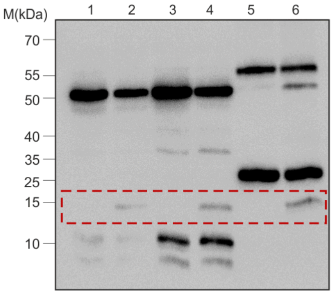

Lower panel
Anti-RNAP antibody

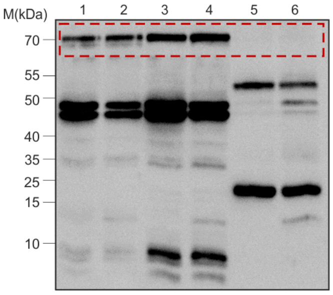

Data related to Fig. 5B

Data refers to the citrate synthase activity levels calculated from the initial velocities, expressed as nmol $min^{-1}mg^{-1}$ of protein input of each lysate sample

	Citrate synthase activity (nmol $min^{-1}mg^{-1}$)		
Lysates extracted from:	Rep I	Rep II	Rep III
WT pCtrl	60.343686	62.858006	59.505579
WT pVcdP	95.228663	103.69343	94.170567
WT pVcdP*	55.2501	50.33898	54.02232
Δ*vcdRP* pCtrl	51.00782	47.819832	43.56918
Δ*vcdRP* pVcdP	92.073491	76.555487	92.073491
Δ*vcdRP* pVcdP*	52.647524	50.198801	45.301357
Δ*gltA* pCtrl	17.027593	21.568285	20.433112
Δ*gltA* pVcdP	13.46072	15.704173	17.947626
Δ*gltA* pVcdP*	13.944615	16.089941	20.380592
F383A pCtrl	71.203929	71.203929	70.156813
F383A pVcdP	61.940757	58.349989	61.940757
F383A pVcdP*	67.013073	67.013073	70.255641

Statistical analysis related to Fig. 5B

ANOVA summary

F	143.6
P value	<0.0001
P value summary	****
Significant diff. among means (P<0.05)?	Yes
R squared	0.9850

Equal variance test (Brown-Forsythe)

F (DFn, DFd)	0.4244 (11, 24)
P value	0.9300
P value summary	ns
Are SDs significantly different (P<0.05)?	No

Normality test (Shapiro-Wilk)

Passed normality test (alpha=0.05)?	Yes

Multiple comparisons

Number of families	1
Number of comparisons per family	66
Alpha	0.05

Tukey's multiple comparisons test	Mean Diff.	95.00% CI of diff.	Below threshold?	Summary	Adjusted P Value
WT pCtrl vs. WT pVcdP	-36.8	-48.02 to -25.57	Yes	****	<0.0001
WT pCtrl vs. WT pVcdP*	7.699	-3.529 to 18.93	No	ns	0.4003
WT pCtrl vs. Δ*vcdRP* pCtrl	13.44	2.209 to 24.66	Yes	*	0.01
WT pCtrl vs. Δ*vcdRP* pVcdP	-26	-37.23 to -14.77	Yes	****	<0.0001
WT pCtrl vs. Δ*vcdRP* pVcdP*	11.52	0.2924 to 22.75	Yes	*	0.0408
WT pCtrl vs. Δ*gltA* pCtrl	41.23	30.00 to 52.45	Yes	****	<0.0001
WT pCtrl vs. Δ*gltA* pVcdP	45.2	33.97 to 56.43	Yes	****	<0.0001
WT pCtrl vs. Δ*gltA* pVcdP*	44.1	32.87 to 55.32	Yes	****	<0.0001
WT pCtrl vs. F383A pCtrl	-9.952	-21.18 to 1.275	No	ns	0.1164
WT pCtrl vs. F383A pVcdP	0.1586	-11.07 to 11.39	No	ns	>0.9999
WT pCtrl vs. F383A pVcdP*	-7.192	-18.42 to 4.036	No	ns	0.4963
WT pVcdP vs. WT pVcdP*	44.49	33.27 to 55.72	Yes	****	<0.0001
WT pVcdP vs. Δ*vcdRP* pCtrl	50.23	39.00 to 61.46	Yes	****	<0.0001
WT pVcdP vs. Δ*vcdRP* pVcdP	10.8	-0.4307 to 22.02	No	ns	0.0671
WT pVcdP vs. Δ*vcdRP* pVcdP*	48.31	37.09 to 59.54	Yes	****	<0.0001
WT pVcdP vs. Δ*gltA* pCtrl	78.02	66.79 to 89.25	Yes	****	<0.0001
WT pVcdP vs. Δ*gltA* pVcdP	81.99	70.77 to 93.22	Yes	****	<0.0001
WT pVcdP vs. Δ*gltA* pVcdP*	80.89	69.67 to 92.12	Yes	****	<0.0001
WT pVcdP vs. F383A pCtrl	26.84	15.62 to 38.07	Yes	****	<0.0001
WT pVcdP vs. F383A pVcdP	36.95	25.73 to 48.18	Yes	****	<0.0001
WT pVcdP vs. F383A pVcdP*	29.6	18.38 to 40.83	Yes	****	<0.0001
WT pVcdP* vs. Δ*vcdRP* pCtrl	5.738	-5.489 to 16.97	No	ns	0.7803
WT pVcdP* vs. Δ*vcdRP* pVcdP	-33.7	-44.92 to -22.47	Yes	****	<0.0001
WT pVcdP* vs. Δ*vcdRP* pVcdP*	3.821	-7.406 to 15.05	No	ns	0.9809
WT pVcdP* vs. Δ*gltA* pCtrl	33.53	22.30 to 44.75	Yes	****	<0.0001
WT pVcdP* vs. Δ*gltA* pVcdP	37.5	26.27 to 48.73	Yes	****	<0.0001
WT pVcdP* vs. Δ*gltA* pVcdP*	36.4	25.17 to 47.63	Yes	****	<0.0001
WT pVcdP* vs. F383A pCtrl	-17.65	-28.88 to -6.424	Yes	***	0.0004
WT pVcdP* vs. F383A pVcdP	-7.54	-18.77 to 3.687	No	ns	0.4294
WT pVcdP* vs. F383A pVcdP*	-14.89	-26.12 to -3.663	Yes	**	0.0033
Δ*vcdRP* pCtrl vs. Δ*vcdRP* pVcdP	-39.44	-50.66 to -28.21	Yes	****	<0.0001
Δ*vcdRP* pCtrl vs. Δ*vcdRP* pVcdP*	-1.917	-13.14 to 9.311	No	ns	>0.9999
Δ*vcdRP* pCtrl vs. Δ*gltA* pCtrl	27.79	16.56 to 39.02	Yes	****	<0.0001
Δ*vcdRP* pCtrl vs. Δ*gltA* pVcdP	31.76	20.53 to 42.99	Yes	****	<0.0001
Δ*vcdRP* pCtrl vs. Δ*gltA* pVcdP*	30.66	19.43 to 41.89	Yes	****	<0.0001
Δ*vcdRP* pCtrl vs. F383A pCtrl	-23.39	-34.62 to -12.16	Yes	****	<0.0001
Δ*vcdRP* pCtrl vs. F383A pVcdP	-13.28	-24.51 to -2.051	Yes	*	0.0113
Δ*vcdRP* pCtrl vs. F383A pVcdP*	-20.63	-31.86 to -9.401	Yes	****	<0.0001
Δ*vcdRP* pVcdP vs. Δ*vcdRP* pVcdP*	37.52	26.29 to 48.75	Yes	****	<0.0001
Δ*vcdRP* pVcdP vs. Δ*gltA* pCtrl	67.22	56.00 to 78.45	Yes	****	<0.0001

Δ*vcdRP* pVcdP vs. Δ*gltA* pVcdP	71.2	59.97 to 82.42	Yes	****	<0.0001
Δ*vcdRP* pVcdP vs. Δ*gltA* pVcdP*	70.1	58.87 to 81.32	Yes	****	<0.0001
Δ*vcdRP* pVcdP vs. F383A pCtrl	16.05	4.818 to 27.27	Yes	**	0.0014
Δ*vcdRP* pVcdP vs. F383A pVcdP	26.16	14.93 to 37.38	Yes	****	<0.0001
Δ*vcdRP* pVcdP vs. F383A pVcdP*	18.81	7.579 to 30.03	Yes	***	0.0002
Δ*vcdRP* pVcdP* vs. Δ*gltA* pCtrl	29.71	18.48 to 40.93	Yes	****	<0.0001
Δ*vcdRP* pVcdP* vs. Δ*gltA* pVcdP	33.68	22.45 to 44.91	Yes	****	<0.0001
Δ*vcdRP* pVcdP* vs. Δ*gltA* pVcdP*	32.58	21.35 to 43.80	Yes	****	<0.0001
Δ*vcdRP* pVcdP* vs. F383A pCtrl	-21.47	-32.70 to -10.24	Yes	****	<0.0001
Δ*vcdRP* pVcdP* vs. F383A pVcdP	-11.36	-22.59 to -0.1338	Yes	*	0.0456
Δ*vcdRP* pVcdP* vs. F383A pVcdP*	-18.71	-29.94 to -7.484	Yes	***	0.0002
Δ*gltA* pCtrl vs. Δ*gltA* pVcdP	3.972	-7.255 to 15.20	No	ns	0.9748
Δ*gltA* pCtrl vs. Δ*gltA* pVcdP*	2.871	-8.356 to 14.10	No	ns	0.9981
Δ*gltA* pCtrl vs. F383A pCtrl	-51.18	-62.41 to -39.95	Yes	****	<0.0001
Δ*gltA* pCtrl vs. F383A pVcdP	-41.07	-52.29 to -29.84	Yes	****	<0.0001
Δ*gltA* pCtrl vs. F383A pVcdP*	-48.42	-59.65 to -37.19	Yes	****	<0.0001
Δ*gltA* pVcdP vs. Δ*gltA* pVcdP*	-1.101	-12.33 to 10.13	No	ns	>0.9999
Δ*gltA* pVcdP vs. F383A pCtrl	-55.15	-66.38 to -43.92	Yes	****	<0.0001
Δ*gltA* pVcdP vs. F383A pVcdP	-45.04	-56.27 to -33.81	Yes	****	<0.0001
Δ*gltA* pVcdP vs. F383A pVcdP*	-52.39	-63.62 to -41.16	Yes	****	<0.0001
Δ*gltA* pVcdP* vs. F383A pCtrl	-54.05	-65.28 to -42.82	Yes	****	<0.0001
Δ*gltA* pVcdP* vs. F383A pVcdP	-43.94	-55.17 to -32.71	Yes	****	<0.0001
Δ*gltA* pVcdP* vs. F383A pVcdP*	-51.29	-62.52 to -40.06	Yes	****	<0.0001
F383A pCtrl vs. F383A pVcdP	10.11	-1.116 to 21.34	No	ns	0.1052
F383A pCtrl vs. F383A pVcdP*	2.761	-8.466 to 13.99	No	ns	0.9987
F383A pVcdP vs. F383A pVcdP*	-7.35	-18.58 to 3.877	No	ns	0.4654

Data related to Fig. 5C

Data refers to the citrate synthase activity levels calculated from the initial velocities, expressed as nmol min^{-1}mg^{-1} of protein input

	Citrate synthase activity (nmol min^{-1}mg^{-1})					
	GltA			F383A		
	Rep I	Rep II	Rep III	Rep I	Rep II	Rep III
Protein only	56.47225	54.77808	52.51919	51.29562	54.9596	56.42519
1x VcdP	67.7667	62.11947	62.11947	58.62357	59.35637	62.28754
2x VcdP	79.06115	76.23753	70.59031	62.28754	63.02034	65.21872
5x VcdP	84.70837	83.0142	85.83782	80.60741	73.27946	72.54667
10x VcdP	100.4128	99.24175	95.14713	85.41284	84.12974	83.17425
15x VcdP	122.475	121.7439	111.7437	89.1754	88.71235	91.5412
20xVcdP	137.4852	139.4126	141.2743	98.74125	99.97123	103.1746
20x VcdP*	65.23488	66.30371	69.50209	57.57752	63.44807	61.44807

Statistical analysis related to Fig. 5C

ANOVA table	SS	DF	MS	F (DFn, DFd)	P value
Interaction	1817	7	259.6	F (7, 32) = 30.11	P<0.0001
Row Factor	21956	7	3137	F (7, 32) = 363.8	P<0.0001
Column Factor	2391	1	2391	F (7, 32) = 277.3	P<0.0001
Residual	275.9	32	8.622		

Normality test (Shapiro-Wilk)
Passed normality test (alpha=0.05)? Yes

Multiple comparisons

Number of families	2
Number of comparisons per family	7
Alpha	0.05

Dunnett's multiple comparisons test	Mean Diff.	95.00% CI of diff.	Below threshold?	Summary	Adjusted P Value
GltA					
Protein only vs. 1x VcdP	-9.412	-16.03 to -2.791	Yes	**	0.0026
Protein only vs. 2x VcdP	-20.71	-27.33 to -14.09	Yes	****	<0.0001
Protein only vs. 5x VcdP	-29.93	-36.55 to -23.31	Yes	****	<0.0001
Protein only vs. 10x VcdP	-43.68	-50.30 to -37.06	Yes	****	<0.0001
Protein only vs. 15x VcdP	-64.06	-70.69 to -57.44	Yes	****	<0.0001
Protein only vs. 20xVcdP	-84.8	-91.42 to -78.18	Yes	****	<0.0001
Protein only vs. 20x VcdP*	-12.42	-19.05 to -5.802	Yes	**	0.0057
F383A					
Protein only vs. 1x VcdP	-5.862	-12.48 to 0.7589	No	ns	0.0999
Protein only vs. 2x VcdP	-9.282	-15.90 to -2.661	Yes	**	0.0031
Protein only vs. 5x VcdP	-21.25	-27.87 to -14.63	Yes	****	<0.0001
Protein only vs. 10x VcdP	-30.01	-36.63 to -23.39	Yes	****	<0.0001
Protein only vs. 15x VcdP	-35.58	-42.20 to -28.96	Yes	****	<0.0001
Protein only vs. 20xVcdP	-46.4	-53.02 to -39.78	Yes	**	0.0033
Protein only vs. 20x VcdP*	-6.598	-13.22 to 0.02354	No	ns	0.511
