# Supplementary material for: A dual‐function RNA balances carbon uptake and central metabolism in Vibrio cholerae
Source: EMBO J. 2021 Oct 6;40(24):e108542. doi: 10.15252/embj.2021108542 (PMC8672173; doi:10.15252/embj.2021108542)
Supplement: Supplementary file 10 [file EMBJ-40-e108542-s003.pdf]

THE EMBO JOURNAL

A dual-function RNA balances carbon uptake and central metabolism in *Vibrio cholerae*

Kai Papenfort, Kavyaa Venkat, Mona Hoyos, James Haycocks, Liam Cassidy, Beatrice Engelmann, Ulrike Rolle-Kampczyk, Martin von Bergen, Andreas Tholey, and David Grainger
DOI: 10.15252/embj.2021108542

Corresponding author: Kai Papenfort (kai.papenfort@uni-jena.de)

Review Timeline:

Submission Date:	26th Apr 21
Editorial Decision:	9th Jun 21
Revision Received:	16th Jul 21
Editorial Decision:	31st Aug 21
Revision Received:	31st Aug 21
Accepted:	2nd Sep 21

Editor: Stefanie Boehm

Transaction Report:

(Note: With the exception of the correction of typographical or spelling errors that could be a source of ambiguity, letters and reports are not edited. Depending on transfer agreements, referee reports obtained elsewhere may or may not be included in this compilation. Referee reports are anonymous unless the Referee chooses to sign their reports.)

1st Editorial Decision 9th Jun 2021

9th Jun 2021

Re: EMBOJ-2021-108542
A dual-function RNA balancing carbon uptake and central metabolism in Vibrio cholerae

Dear Kai,

Thank you again for submitting your manuscript to The EMBO Journal. We have now received three referee reports on your study, which are included below for your information. In light of the referees' comments, I am happy to say that we would like to invite you to prepare and submit a revised manuscript.

As you will see, the reviewers are overall positive and appreciate the study and the interest of the findings to the field. However, they do raise some concerns, which will need to be addressed in a revised version. Specifically, in particular referee #1 and referee #3, express concerns regarding the small effect of the VcdP peptide on GltA activity and the proposed model of it counteracting the inhibition by NADH. Please carefully consider the referees' arguments and suggestions, and provide additional supportive data and revise the manuscript accordingly. This point must be addressed. In addition, all referees are not fully convinced that an effect on cholera toxin production has been directly shown (ref #1- summary; ref #2- point 5; ref #3- paragraph 2, 6), please revise the conclusions where necessary. In addition, please also respond to all other referee comments and add the request additional information on methods and controls, as well as revising the manuscript and figures where applicable.

Please note that it is our policy to allow only a single round of major revision. Acceptance depends on a positive outcome of the second round of review and therefore on the completeness of your responses included in the next, final version of the manuscript. We realize that lab work worldwide is currently affected by the COVID-19/SARS-CoV-2 pandemic and that an experimental revision may be delayed. We can extend the revision time when needed, and we have extended our 'scooping protection policy' to cover the period required for a full revision. However, it is nonetheless important to clarify any questions and concerns at this stage, and we encourage you to discuss a revision plan or any potential issues you may foresee as soon as possible.

Please also feel free to contact me should you have any other further questions. Thank you for the opportunity to consider your work for publication. I look forward to receiving your revised manuscript.

Kind regards,

Stefanie

Stefanie Boehm
Editor
The EMBO Journal

Referee #1:

This exceptionally thorough and well-done study provides interesting new insight into how dual function small RNAs can tweak central metabolism in Vibrio. The strengths of this study are that the authors start with an open screen for sRNAs that inhibit cholera toxin production that leads to the identification of VcdRP, and then they follow up the strongest candidates with very thorough tests for the proposed regulatory interaction. This strategy (general to specific) is followed at each step of the story, building an impressive level of support for the interactions shown in Fig. 7. The data and the analysis are very high quality, and the manuscript is well written with attractive figures.

The findings themselves are quite interesting - the sRNA targets glycolytic enzymes whereas the peptide interacts with citrate synthase in the TCA cycle. One has to be cautious about interpreting how this ultimately contributes to cholera toxin production - although the shifts in metabolite concentrations in Fig. 6 are fascinating, the feedback control mechanisms are complex.

My only concern is that the VcdP peptide has quite a modest effect on citrate synthase activity. It's highly encouraging that the authors get a linear dose-dependent increase in activity with increasing amounts of peptide, but 5X amount results in only 20% higher activity, and the effect doesn't seem to be anywhere near saturation. The authors may want to consider whether VcdP is expressed at sufficient levels in V. cholerae to directly affect the activity of a such an abundant enzyme, if the interaction is so weak (or ineffective).

Apart from these caveats and some specific points below, this very excellent study adds considerably to our current understanding of how dual function RNAs can optimize bacterial gene expression in different growth conditions and in ways that affect virulence.

Specific suggestions for improvement:

1. Methods and Fig. 1A - please provide more information on how CTX levels were detected (what type of antibody, for example?) and quantified, so the reader doesn't have to refer back to earlier papers.

2. Why do the authors think they see shorter fragments of VcdRP in Fig. 2 - are they just degradation products, or is the RNA being processed? Can they comment on this?

3. In Fig. 3C and line 204, could the authors please indicate somehow the magnitudes of the regulatory effects of VcdRP overexpression (perhaps as a range)? I would have found this more useful than the Venn diagram. It would also help put in perspective the targets they select to follow up.

4. Please provide more information about the activity assays shown in Fig. 5B,C, perhaps in a supplementary figure. Are these measurements from single endpoints? Initial velocities? Was the increase in product linear over time? How do these measurements compare to values from the literature?

5. Although the VcdP* mutant does not increase the activity of citrate synthase in extracts (Fig. 5A), I am not sure the authors can conclude this has to do with NADH inhibition, because in the purified system, the mutant peptide is able to activate citrate synthase.

6. The light blue, green and gray symbols and lines are hard to see, especially on shaded backgrounds. Consider using heavier lines or slightly darker hues.

Referee #2:

I enjoyed reading two manuscripts which deals with dual function sRNAs in E. coli and V. cholerae though it was challenging for me to review the papers together.

The Papenfort group showed previously that the quorum sensing-controlled VqmR sRNA inhibits the production of the cholera toxin (CTX) in V. cholerae. In the present work, Venkat et al. find first that overexpression of an Hfq-binding sRNA (called VcdRP) significantly reduces CTX production and the sRNA encodes a small protein (VcdP). They show that the VcdRP transcription is repressed by CRP-cAMP. Then, the authors demonstrate that overexpression of VcdRP affects the expression of a large number of genes. A major finding is that VcdRP RNA inhibits translation of mRNAs encoding sugar transport proteins by bae-pairing mechanism. In addition, they have found that VcdP binds to GltA and enhances its activity both in vivo and in vitro. Finally, the levels of metabolic intermediates of glycolysis and citric acid cycle have been shown to vary inversely by the deletion of vcdRP or VcdRP overexpression. Taken together, the authors conclude that VcdRP acts as a dual function sRNA to modulate carbohydrate uptake and utilization, affecting the CTX production.
This work clearly shows that VcdRP modulates carbon metabolism extensively acting as a dual function RNA in V. cholerae. The data are well documented by a variety of experiments to support the conclusion and most arguments sound. I have no serious criticisms on this manuscript except several questions and comments which might be useful to improve the manuscript.
1) Shorter RNA species in addition to the full-length VcdRP are generated in cells (Fig. 1). It is interesting to investigate how this occurs and whether the shorter species are functional.
2) Transcriptomic analyses have shown that expression of a large number of genes varies in

response to overexpression of VcdRP (Fig. 3). The readers also want to know how the vcdRP null-mutation affects the expression of these genes.
3) Is the translational repression of PTS-coding mRNAs by VcdRP (VcdR) dependent on Hfq (Fig. 4)?
4) The observation that the 3' end of VcdRP contains the base pairing region and is sufficient for translational repression of ptsG suggests that the shorter species of VcdRP would be also functional to inhibit translational repression of PTS-coding mRNA.
5) It is an interesting question how the translational repression of PTS-coding mRNAs by VcdRP leads to the reduction of CTX production. The authors claim that the increased cAMP levels caused by the inhibition of ptsHI expression through VcdRP overexpression is responsible for the reduction of CTX production. If this is the case, addition of a high concentration of external cAMP is expected to lower CTX level without VcdRP overexpression.
6) The authors argue that VcdRP (VcdP) affects the metabolic profile through enhancing GltA activity because VcdRP effect is no longer observed in the absence of GltA. It is also interesting to examine whether the similar effect on the metabolic profile occurs when GltA activity is enhanced by other ways, for example by GltA overexpression.

Referee #3:

The Papenfort group presents an interesting story concerning the biological roles of, and mechanistic insights into, a dual function RNA in Vibrio cholerae, called VcdRP. Even though a first example in RNAIII (S. aureus) has been published a long time ago, only more recently have many more cases shown up where an RNA functions as both an antisense (regulatory) RNA and simultaneously as an mRNA that encodes a small protein. The back-to-back paper by the Storz group describes another new example.

Virulence is greatly affected by production of the cholera toxin CTX. Using a forward genetic screen, the authors tested a battery of ectopically expressed sRNAs for effects on CTX expression. One of these, called VcdR, inhibited CTX accumulation. This sRNA subsequently turned out to both a riboregulator and an mRNA encoding a small protein. Expression studies revealed transcriptional repression by CRP-cAMP, suggestion effects related to glucose levels/ transport. Inspection of the sRNA sequence indicated the presence of a short protein reading frame which was translated in vivo (tagging/ fusions).

Mutant variants of the sRNA suggested early on that CTX was controlled by the RNA rather than the protein element. The separation of RNA versus protein effects of the dual-function RNA was further analyzed. In its riboregulator mode, VcdR (the RNA-alone version, stop codon in ORF) appears to use primarily a C-4 region near its 3'end to base-pair to the translation initiation sites of the ptsG, treB, and nagE mRNAs, that produce the glucose-, trehalose-, and N-acetylglucosamine-specific components of the phosphotransferase carbohydrate transporter (PTS). In addition, VcdR also represses the ptsHI mRNA that encodes two sugar non-specific phosphocarrier proteins required for carbohydrate uptake. All in all, these targets are centered around PTS and carbon uptake systems, and negative regulation is nicely validated by reporter assays with appropriate specificity controls (mutations in the interaction sites).

Transcriptomics experiments were used to map RNA-only versus protein-only effects, and gave few initial leads as to what VcdP does. The most highly repressed gene, vca0053, encodes purine

nucleoside phosphorylase. Ultimately, the authors chose to overexpress VcdP to find interaction partners by immunoprecipitation. This gave one single clear hit which by MS was identified as GltA (citrate synthase). GltA is the first enzyme of the citric acid cycle and catalyzes the condensation of oxaloacetate and acetyl coenzyme A to produce citrate and coenzyme A. Interaction was validated by reciprocal pulldown. Interestingly, overexpression of VcdP did not affect GltA levels, but rather boosted GltA activity in cell extracts, and in vitro. A mutant VcdP failed to active.

The authors consider a possible mechanism for activation that involves VcdP counteracting the known inhibitory effect of NADH on GltA activity. Possibly in support of this proposal, a mutant of GltA which is insensitive to NADH is unaffected by VcdP. In any case, in vitro experiments do show a fairly small dose-dependent effect of VcdP binding on GltA activity.

Finally, a mass spec analysis was conducted on wt/ ΔvcdRP cells, with empty vectors or R or P ectopically expressed. This resulted in a complex picture, but essentially reaffirmed that the R element affects carbohydrate uptake/ consumption, whereas P does not. Thus, the protein element likely acts downstream, and probably most effects of P are therefore via GltA. This is summarized in Fig 6 (I must admit, this Figure was a bit overwhelming).

As nicely indicated in the model (Fig 7), the combined data set makes a strong case for a scenario in which the dual-function RNA VcdRP regulates/ modulates the citric acid cycle and glycolysis pathway.

The paper is well-written, asks clear and important questions, provides generally well-conducted experiments, and has a strong message. The Discussion part is enjoyable (I also liked the nod to the Lenski experiments).

Minor points:

*Add size marker to Fig 2A

*I was a bit lost where the vca0053 trail was heading (might have been my ignorance), around line 280 or so. Maybe this could be made a bit clearer.

*Line 277: I understand that VcdP SPA-tagging (sequential peptide affinity) appears to work well for your co-immunoprecipitation experiments. For many readers, the information given is cryptic. E.g. anti-FLAG antibodies were used, which only makes sense if one knows that the tag has FLAG sequences. Please add more information, Incidentally, the methods paper referred to could not be downloaded. M&M also is a bit cryptic, lines 522 ff. Unclear how the interactants were identified. Should be easy to write some explanatory lines.

*A mistake that should be fixed for the sake of family peace: Fröhlich not Frohlich

Referee #1:

This exceptionally thorough and well-done study provides interesting new insight into how dual function small RNAs can tweak central metabolism in Vibrio. The strengths of this study are that the authors start with an open screen for sRNAs that inhibit cholera toxin production that leads to the identification of VcdRP, and then they follow up the strongest candidates with very thorough tests for the proposed regulatory interaction. This strategy (general to specific) is followed at each step of the story, building an impressive level of support for the interactions shown in Fig. 7. The data and the analysis are very high quality, and the manuscript is well written with attractive figures.

The findings themselves are quite interesting - the sRNA targets glycolytic enzymes whereas the peptide interacts with citrate synthase in the TCA cycle. One has to be cautious about interpreting how this ultimately contributes to cholera toxin production - although the shifts in metabolite concentrations in Fig. 6 are fascinating, the feedback control mechanisms are complex.

My only concern is that the VcdP peptide has quite a modest effect on citrate synthase activity. It's highly encouraging that the authors get a linear dose-dependent increase in activity with increasing amounts of peptide, but 5X amount results in only 20% higher activity, and the effect doesn't seem to be anywhere near saturation. The authors may want to consider whether VcdP is expressed at sufficient levels in V. cholerae to directly affect the activity of a such an abundant enzyme, if the interaction is so weak (or ineffective).

Apart from these caveats and some specific points below, this very excellent study adds considerably to our current understanding of how dual function RNAs can optimize bacterial gene expression in different growth conditions and in ways that affect virulence.

We thank the reviewer for her/his positive assessment of our work. We have now tested GltA activity in the presence of 10-, 15-, and 20- times higher VcdP concentrations, which allowed us to score up to ~250% higher GltA activity (at 20-fold VcdP excess). In line with our previous data, we did not observe efficient activation of GltA when we added the mutated VcdP* variant (also provided in 20-fold excess). These new data are presented in Fig. 5C of our revised manuscript.

Specific suggestions for improvement:

1. Methods and Fig. 1A - please provide more information on how CTX levels were detected (what type of antibody, for example?) and quantified, so the reader doesn't have to refer back to earlier papers.

As requested by the referee, we now provided a detailed description of these experiments in the Materials and Methods section (Page 18, lines 610-635).

2. Why do the authors think they see shorter fragments of VcdRP in Fig. 2 - are they just degradation products, or is the RNA being processed? Can they comment on this?

Our previous work on ribonuclease E (RNase E, see Hoyos et al 2020, eLIFE) in *V. cholerae* suggested that VcdRP might be subject to RNase E-mediated degradation. In our revised manuscript, we thus tested the expression of VcdRP in a temperature-sensitive RNase E (*rne*TS) strain (see new Fig. EV1A). Indeed, the new data supports the idea that VcdRP is subject to RNase E-mediated decay, which we now also discuss in the results section of the revised manuscript (Page 5, lines 130-135).

3. In Fig. 3C and line 204, could the authors please indicate somehow the magnitudes of the regulatory

effects of VcdRP overexpression (perhaps as a range)? I would have found this more useful than the Venn diagram. It would also help put in perspective the targets they select to follow up.

We thank the reviewer for this comment and agree that Venn diagrams are not always intuitive and may not provide all the information contained in the results of the experiment. However, since found it difficult to add more information into the Venn diagram, we decided to expand and improve Appendix Table S1 so that it matches the color-code of Fig. 3A. We hope that this will enhance the accessibility of the results and help the reader to navigate through the data.

4. Please provide more information about the activity assays shown in Fig. 5B,C, perhaps in a supplementary figure. Are these measurements from single endpoints? Initial velocities? Was the increase in product linear over time? How do these measurements compare to values from the literature?

As requested, we now provide a detailed description of the method and the data analysis in our revised materials and methods section (Page 20, lines 695-720). In addition, we expanded the source data file associated with these experiments, which will be published together with the manuscript.

5. Although the VcdP* mutant does not increase the activity of citrate synthase in extracts (Fig. 5A), I am not sure the authors can conclude this has to do with NADH inhibition, because in the purified system, the mutant peptide is able to activate citrate synthase.

As pointed out above, we have now revised Fig. 5C and discovered that even when provided at 20-fold excess, VcdP* only mildly affected GltA activity and we did not observe any significant effect of VcdP* on GltA activity in cell lysates (Fig. 5B). Nevertheless, given that we still find significant activation of the GltA F383 variant by VcdP (although to a much weaker extent when compared to wild-type GltA), we agree with the referee that modulation of the interaction of GltA with NADH by VcdP is likely insufficient to explain the underlying regulatory mechanism. We therefore softened our interpretation of these results in the corresponding section of the results (Page 10, lines 335-350). We now write:" Together, we conclude that VcdP binds to and activates GltA in vivo and in vitro, which might also affect the interaction of GltA with NADH."

6. The light blue, green and gray symbols and lines are hard to see, especially on shaded backgrounds. Consider using heavier lines or slightly darker hues.

As requested by the referee, we have improved the coloring in Fig. 6 in our revised manuscript.

Referee #2:
I enjoyed reading two manuscripts which deals with dual function sRNAs in E. coli and V. cholerae though it was challenging for me to review the papers together.

The Papenfort group showed previously that the quorum sensing-controlled VqmR sRNA inhibits the production of the cholera toxin (CTX) in V. cholerae. In the present work, Venkat et al. find first that overexpression of an Hfq-binding sRNA (called VcdRP) significantly reduces CTX production and the sRNA encodes a small protein (VcdP). They show that the VcdRP transcription is repressed by CRP-cAMP. Then, the authors demonstrate that overexpression of VcdRP affects the expression of a large number of genes. A major finding is that VcdRP RNA inhibits translation of mRNAs encoding sugar transport proteins by bae-pairing mechanism. In addition, they have found that VcdP binds to GltA and enhances its activity both in vivo and in vitro. Finally, the levels of metabolic intermediates of

glycolysis and citric acid cycle have been shown to vary inversely by the deletion of vcdRP or VcdRP overexpression. Taken together, the authors conclude that VcdRP acts as a dual function sRNA to modulate carbohydrate uptake and utilization, affecting the CTX production.
This work clearly shows that VcdRP modulates carbon metabolism extensively acting as a dual function RNA in V. cholerae. The data are well documented by a variety of experiments to support the conclusion and most arguments sound. I have no serious criticisms on this manuscript except several questions and comments which might be useful to improve the manuscript.

We would to thank the reviewer for the thorough review of our work. We have addressed all comments and questions in detail below.

1) Shorter RNA species in addition to the full-length VcdRP are generated in cells (Fig. 1). It is interesting to investigate how this occurs and whether the shorter species are functional.

As pointed out in more detail in response to comment #2 of reviewer 1, we now show that RNase E is involved in VcdRP turn-over (new Fig. EV1A). We also tested the functionality of several of these species (see detailed response to comment #4 below).

2) Transcriptomic analyses have shown that expression of a large number of genes varies in response to overexpression of VcdRP (Fig. 3). The readers also want to know how the vcdRP null-mutation affects the expression of these genes.
Following the referee's suggestion, in our revised manuscript, we have now compared the expression of 10 differentially regulated genes in wild-type, Δ*vcdRP*, and VcdRP over-expressing cells. These experiments included 5 genes that are regulated by VcdR (*ptsG*, *treB*, *nagE*, *ptsH*, and *ptsI*) and 5 genes that are regulated by VcdP (*vca0053*, *vc2761*, *vc1953*, *vc1449*, and *lamB*). In all cases, we observed the expected trend in regulation, *i.e.* genes that were down-regulated by VcdR and/or VcdP were up-regulated in the Δ*vcdRP* strain and vice-versa up-regulated genes were repressed. These new data are presented in Fig. EV2C of our revised manuscript.

3) Is the translational repression of PTS-coding mRNAs by VcdRP (VcdR) dependent on Hfq (Fig. 4)?

In our revised manuscript, we have now tested repression of the *pts* mRNAs by VcdR in Δ*hfq* cells and in all cases we discovered that regulation was fully abrogated (see Figs. EV3H-I). Hence, we conclude that VcdR acts together with Hfq to mediate target gene regulation.

4) The observation that the 3' end of VcdRP contains the base pairing region and is sufficient for translational repression of ptsG suggests that the shorter species of VcdRP would be also functional to inhibit translational repression of PTS-coding mRNA.

We thank the reviewer for this comment. Indeed, the 156 bp, 87 bp, and 71 bp variants of VcdRP correspond to processing products discovered in Fig. EV1A and in our previous work on RNase E in *V. cholerae* (see Hoyos et al 2020, eLIFE). Testing these variants for regulation of PtsG::GFP, TreB::GFP, NagE::GFP, and PtsHI::GFP showed that variants 156 bp and 87 bp are capable of target repression, while variant 71bp is not (see Figs. EV3B-F of the revised manuscript). Therefore, we speculate that several of the shorter VcdRP variants are capable of repressing target genes. However, cleavage at position 71 generates an inactive sRNA variant.

5) It is an interesting question how the translational repression of PTS-coding mRNAs by VcdRP leads to the reduction of CTX production. The authors claim that the increased cAMP levels caused by the inhibition of ptsHI expression through VcdRP overexpression is responsible for the reduction of CTX

production. If this is the case, addition of a high concentration of external cAMP is expected to lower CTX level without VcdRP overexpression.

To bolster our previous hypothesis, in our revised manuscript we compared the expression of *ctxA* and *ctxB* mRNAs in wild-type and Δ*cyaA* cells, as well as in the absence or presence of external cAMP. The results of these experiments showed that *ctxA* and *ctxB* mRNA levels were increased ~5-fold and ~6-fold, respectively, in the *cyaA* mutant and reduced in the presence of external cAMP. These new data are presented in Fig. EV5 of our revised manuscript and we have extended the manuscript text accordingly (Page 12, lines 385-400) to accommodate these results.

6) The authors argue that VcdRP (VcdP) affects the metabolic profile through enhancing GltA activity because VcdRP effect is no longer observed in the absence of GltA. It is also interesting to examine whether the similar effect on the metabolic profile occurs when GltA activity is enhanced by other ways, for example by GltA overexpression.

Following the reviewers request, we generated a GltA over-expression plasmid, which we conjugated into *V. cholerae*. Unfortunately, we noticed that this plasmid affected growth of *V. cholerae*. To circumvent this problem, we designed an inducible pBAD-GltA plasmid, which we also moved into *V. cholerae*. We next cultivated this strain in the presence of L-arabinose into exponential growth (OD_{600}=0.5) and measured α-ketoglutarate and glutamate levels using mass spectrometry. When compared to our control strain (carrying an empty vector control), we discovered that pBAD-GltA expression resulted in increased α-ketoglutarate levels (analogous to VcdP over-expression, Fig. 6), whereas glutamate levels did not change significantly. We thus conclude that the effects of GltA and VcdP over-expression partially overlap. However, given the problems encountered with constitutive GltA over-expression and the differences in growth conditions between this new experiment and the experiment presented in Fig. 6, we prefer to not include the results from the pBAD-GltA plasmid in the revised manuscript as the data is not strictly comparable. Nevertheless, we have added these data below for the reviewer's approval.

[Unpublished data removed at authors' request]

Referee #3:
The Papenfort group presents an interesting story concerning the biological roles of, and mechanistic insights into, a dual function RNA in Vibrio cholerae, called VcdRP. Even though a first example in RNAIII (S. aureus) has been published a long time ago, only more recently have many more cases shown up where an RNA functions as both an antisense (regulatory) RNA and simultaneously as an mRNA that encodes a small protein. The back-to-back paper by the Storz group describes another new example.

Virulence is greatly affected by production of the cholera toxin CTX. Using a forward genetic screen, the authors tested a battery of ectopically expressed sRNAs for effects on CTX expression. One of these, called VcdR, inhibited CTX accumulation. This sRNA subsequently turned out to both a riboregulator and an mRNA encoding a small protein. Expression studies revealed transcriptional repression by CRP-cAMP, suggestion effects related to glucose levels/ transport. Inspection of the sRNA sequence indicated the presence of a short protein reading frame which was translated in vivo (tagging/ fusions).

Mutant variants of the sRNA suggested early on that CTX was controlled by the RNA rather than the protein element. The separation of RNA versus protein effects of the dual-function RNA was further analyzed. In its riboregulator mode, VcdR (the RNA-alone version, stop codon in ORF) appears to use primarily a C-4 region near its 3'end to base-pair to the translation initiation sites of the ptsG, treB, and nagE mRNAs, that produce the glucose-, trehalose-, and N-acetylglucosamine-specific components of the phosphotransferase carbohydrate transporter (PTS). In addition, VcdR also represses the ptsHI mRNA that encodes two sugar non-specific phosphocarrier proteins required for carbohydrate uptake. All in all, these targets are centered around PTS and carbon uptake systems, and negative regulation is nicely validated by reporter assays with appropriate specificity controls (mutations in the interaction sites).

Transcriptomics experiments were used to map RNA-only versus protein-only effects, and gave few initial leads as to what VcdP does. The most highly repressed gene, vca0053, encodes purine nucleoside phosphorylase. Ultimately, the authors chose to overexpress VcdP to find interaction partners by immunoprecipitation. This gave one single clear hit which by MS was identified as GltA (citrate synthase). GltA is the first enzyme of the citric acid cycle and catalyzes the condensation of oxaloacetate and acetyl coenzyme A to produce citrate and coenzyme A. Interaction was validated by reciprocal pulldown. Interestingly, overexpression of VcdP did not affect GltA levels, but rather boosted GltA activity in cell extracts, and in vitro. A mutant VcdP failed to active.

The authors consider a possible mechanism for activation that involves VcdP counteracting the known inhibitory effect of NADH on GltA activity. Possibly in support of this proposal, a mutant of GltA which is insensitive to NADH is unaffected by VcdP. In any case, in vitro experiments do show a fairly small dose-dependent effect of VcdP binding on GltA activity.

Finally, a mass spec analysis was conducted on wt/ ΔvcdRP cells, with empty vectors or R or P ectopically expressed. This resulted in a complex picture, but essentially reaffirmed that the R element affects carbohydrate uptake/ consumption, whereas P does not. Thus, the protein element likely acts downstream, and probably most effects of P are therefore via GltA. This is summarized in Fig 6 (I must admit, this Figure was a bit overwhelming).

As nicely indicated in the model (Fig 7), the combined data set makes a strong case for a scenario in which the dual-function RNA VcdRP regulates/ modulates the citric acid cycle and glycolysis pathway.

The paper is well-written, asks clear and important questions, provides generally well-conducted experiments, and has a strong message. The Discussion part is enjoyable (I also liked the nod to the Lenski experiments).

We thank this reviewer for careful reading of our manuscript and helpful suggestions. As pointed out in response to the main comment raised by referee #1, in our revised manuscript, we tested activation of GltA by VcdP at several additional concentrations and now report ~2.5-fold higher GltA activity when a 20X excess of VcdP over GltA is provided (Fig. 5C of our revised manuscript). We also updated Fig. 6 to improve data accessibility (also in accordance with point #6 of reviewer 1).

Minor points:

*Add size marker to Fig 2A

As requested size markers were added to Fig. 2A.

*I was a bit lost where the vca0053 trail was heading (might have been my ignorance), around line 280 or so. Maybe this could be made a bit clearer.

We thank the reviewer for this suggestion and edited the manuscript text accordingly (Page 9, lines 275-290). The purpose of these experiments was to determine if regulation by VcdP occurs at the transcriptional or post-transcriptional level. Therefore, we decided to study *vca0053*, which was strongly repressed by VcdP, but not affected by VcdR in our transcriptomic experiment (Appendix Table S1). Establishing these reporter assays was crucial for the following section of our study, since it allowed us to confirm the functionality of VcdP::SPA, which we used to co-immunoprecipitate interaction partners of VcdP (Figs. 5A and EV4).

*Line 277: I understand that VcdP SPA-tagging (sequential peptide affinity) appears to work well for your co-immunoprecipitation experiments. For many readers, the information given is cryptic. E.g. anti-FLAG antibodies were used, which only makes sense if one knows that the tag has FLAG sequences. Please add more information, Incidentally, the methods paper referred to could not be downloaded. M&M also is a bit cryptic, lines 522 ff. Unclear how the interactants were identified. Should be easy to write some explanatory lines.

We now provide a detailed description of the procedure in our revised materials and methods section (Page 20, lines 675-690).

*A mistake that should be fixed for the sake of family peace: Fröhlich not Frohlich

We thank the reviewer for this comment and corrected this mistake as requested.

1st Revision - Editorial Decision 31st Aug 2021

Thank you again for submitting your revised manuscript. Please excuse the delay in communicating this decision to you, as mentioned, this was also due to delayed referee responses over the summer holiday period. We have now however received the reports from the two of the initial referees (see comments below) and I am pleased to say that they overall find that their comments have been satisfactorily addressed and now support publication. Therefore, I would now

----

Referee #1:

The authors have very thoroughly addressed the comments of the three referees in their revised manuscript. In particular, they added additional controls and experiments that solidify specific aspects of VcdRP regulation, such as the requirement for Hfq and the effect of cyclic AMP on VcdRP expression. They were also able to demonstrate increased activity of GltA (citrate synthase) by a larger range of VcdP peptide concentrations, bolstering the conclusion that the peptide directly activates the enzyme. Overall, this well-done study provides a wealth of information on this dual function sRNA. The data provide an instructive example of how toxin production is integrated with carbon utilization and with central metabolism. As a result, I believe this paper will also appeal to microbiologists outside the sRNA regulation community.

Referee #3:

I am totally happy with the revised manuscript, and suggest to publish the paper in its current state

The authors performed the requested editorial changes.

2nd Revision - Final Decision **2nd Sep 2021**

Thank you again for submitting the final revised version of your manuscript. I am pleased to inform you that we have now accepted it for publication in The EMBO Journal.

EMBO PRESS

YOU MUST COMPLETE ALL CELLS WITH A PINK BACKGROUND ➔

PLEASE NOTE THAT THIS CHECKLIST WILL BE PUBLISHED ALONGSIDE YOUR PAPER

Corresponding Author Name: Kai Papenfort

Journal Submitted to: EMBO Journal

Manuscript Number: EMBOJ-2021-108542R

Reporting Checklist For Life Sciences Articles (Rev. June 2017)

This checklist is used to ensure good reporting standards and to improve the reproducibility of published results. These guidelines are consistent with the Principles and Guidelines for Reporting Preclinical Research issued by the NIH in 2014. Please follow the journal's authorship guidelines in preparing your manuscript.

A- Figures

1. Data

The data shown in figures should satisfy the following conditions:

- the data were obtained and processed according to the field's best practice and are presented to reflect the results of the experiments in an accurate and unbiased manner.
- figure panels include only data points, measurements or observations that can be compared to each other in a scientifically meaningful way.
- graphs include clearly labeled error bars for independent experiments and sample sizes. Unless justified, error bars should not be shown for technical replicates.
- if n< 5, the individual data points from each experiment should be plotted and any statistical test employed should be justified
- Source Data should be included to report the data underlying graphs. Please follow the guidelines set out in the author ship guidelines on Data Presentation.

2. Captions

Each figure caption should contain the following information, for each panel where they are relevant:

- a specification of the experimental system investigated (eg cell line, species name).
- the assay(s) and method(s) used to carry out the reported observations and measurements
- an explicit mention of the biological and chemical entity(ies) that are being measured.
- an explicit mention of the biological and chemical entity(ies) that are altered/varied/perturbed in a controlled manner.

- the exact sample size (n) for each experimental group/condition, given as a number, not a range;
- a description of the sample collection allowing the reader to understand whether the samples represent technical or biological replicates (including how many animals, litters, cultures, etc.).
- a statement of how many times the experiment shown was independently replicated in the laboratory.
- definitions of statistical methods and measures:
  - common tests, such as t-test (please specify whether paired vs. unpaired), simple χ^2 tests, Wilcoxon and Mann-Whitney tests, can be unambiguously identified by name only, but more complex techniques should be described in the methods section;
  - are tests one-sided or two-sided?
  - are there adjustments for multiple comparisons?
  - exact statistical test results, e.g., P values = x but not P values < x;
  - definition of 'center values' as median or average;
  - definition of error bars as s.d. or s.e.m.

Any descriptions too long for the figure legend should be included in the methods section and/or with the source data.

In the pink boxes below, please ensure that the answers to the following questions are reported in the manuscript itself. Every question should be answered. If the question is not relevant to your research, please write NA (non applicable). We encourage you to include a specific subsection in the methods section for statistics, reagents, animal models and human subjects.

USEFUL LINKS FOR COMPLETING THIS FORM

http://www.antibodypedia.com
http://1degreebio.org
http://www.equator-network.org/reporting-guidelines/improving-bioscience-research-repor
http://grants.nih.gov/grants/olaw/olaw.htm
http://www.mrc.ac.uk/Ourresearch/Ethicsresearchguidance/Useofanimals/index.htm
http://ClinicalTrials.gov
http://www.consort-statement.org
http://www.consort-statement.org/checklists/view/32-consort/66-title
http://www.equator-network.org/reporting-guidelines/reporting-recommendations-for-tumo
http://datadryad.org
http://figshare.com
http://www.ncbi.nlm.nih.gov/gap
http://www.ebi.ac.uk/ega
http://biomodels.net/
http://biomodels.net/miriam/
http://jjj.biochem.sun.ac.za
http://oba.od.nih.gov/biosecurity/biosecurity_documents.html
http://www.selectagents.gov/

B- Statistics and general methods

Please fill out these boxes ➔ (Do not worry if you cannot see all your text once you press return)

1.a. How was the sample size chosen to ensure adequate power to detect a pre-specified effect size?	No estimation of statistical power was used to pre-determine sample size
1.b. For animal studies, include a statement about sample size estimate even if no statistical methods were used.	N.A.
2. Describe inclusion/exclusion criteria if samples or animals were excluded from the analysis. Were the criteria pre-established?	N.A.
3. Were any steps taken to minimize the effects of subjective bias when allocating animals/samples to treatment (e.g. randomization procedure)? If yes, please describe.	N.A.
For animal studies, include a statement about randomization even if no randomization was used.	N.A.
4.a. Were any steps taken to minimize the effects of subjective bias during group allocation or/and when assessing results (e.g. blinding of the investigator)? If yes please describe.	N.A.
4.b. For animal studies, include a statement about blinding even if no blinding was done	N.A.
5. For every figure, are statistical tests justified as appropriate?	Yes
Do the data meet the assumptions of the tests (e.g., normal distribution)? Describe any methods used to assess it.	Yes, normal distribution was tested using Shapiro-Wilk test
Is there an estimate of variation within each group of data?	Yes

Is the variance similar between the groups that are being statistically compared?	Yes, equal variance was tested using Brown-Forsythe test

C- Reagents

6. To show that antibodies were profiled for use in the system under study (assay and species), provide a citation, catalog number and/or clone number, supplementary information or reference to an antibody validation profile. e.g., Antibodypedia (see link list at top right), 1DegreeBio (see link list at top right).	The catalog number of the relevant antibodies are provided in the Materials and Methods section
7. Identify the source of cell lines and report if they were recently authenticated (e.g., by STR profiling) and tested for mycoplasma contamination.	N.A.

* for all hyperlinks, please see the table at the top right of the document

D- Animal Models

8. Report species, strain, gender, age of animals and genetic modification status where applicable. Please detail housing and husbandry conditions and the source of animals.	N.A.
9. For experiments involving live vertebrates, include a statement of compliance with ethical regulations and identify the committee(s) approving the experiments.	N.A.
10. We recommend consulting the ARRIVE guidelines (see link list at top right) (PLoS Biol. 8(6), e1000412, 2010) to ensure that other relevant aspects of animal studies are adequately reported. See author guidelines, under 'Reporting Guidelines'. See also: NIH (see link list at top right) and MRC (see link list at top right) recommendations. Please confirm compliance.	N.A.

E- Human Subjects

11. Identify the committee(s) approving the study protocol.	N.A.
12. Include a statement confirming that informed consent was obtained from all subjects and that the experiments conformed to the principles set out in the WMA Declaration of Helsinki and the Department of Health and Human Services Belmont Report.	N.A.
13. For publication of patient photos, include a statement confirming that consent to publish was obtained.	N.A.
14. Report any restrictions on the availability (and/or on the use) of human data or samples.	N.A.
15. Report the clinical trial registration number (at ClinicalTrials.gov or equivalent), where applicable.	N.A.
16. For phase II and III randomized controlled trials, please refer to the CONSORT flow diagram (see link list at top right) and submit the CONSORT checklist (see link list at top right) with your submission. See author guidelines, under 'Reporting Guidelines'. Please confirm you have submitted this list.	N.A.
17. For tumor marker prognostic studies, we recommend that you follow the REMARK reporting guidelines (see link list at top right). See author guidelines, under 'Reporting Guidelines'. Please confirm you have followed these guidelines.	N.A.

F- Data Accessibility

18: Provide a "Data Availability" section at the end of the Materials & Methods, listing the accession codes for data generated in this study and deposited in a public database (e.g. RNA-Seq data: Gene Expression Omnibus GSE39462, Proteomics data: PRIDE PXD000208 etc.) Please refer to our author guidelines for 'Data Deposition'.<br><br>Data deposition in a public repository is mandatory for:<br>a. Protein, DNA and RNA sequences<br>b. Macromolecular structures<br>c. Crystallographic data for small molecules<br>d. Functional genomics data<br>e. Proteomics and molecular interactions	All accession codes are listed in the Data Availability section
19. Deposition is strongly recommended for any datasets that are central and integral to the study; please consider the journal's data policy. If no structured public repository exists for a given data type, we encourage the provision of datasets in the manuscript as a Supplementary Document (see author guidelines under 'Expanded View' or in unstructured repositories such as Dryad (see link list at top right) or Figshare (see link list at top right).	N.A.
20. Access to human clinical and genomic datasets should be provided with as few restrictions as possible while respecting ethical obligations to the patients and relevant medical and legal issues. If practically possible and compatible with the individual consent agreement used in the study, such data should be deposited in one of the major public access-controlled repositories such as dbGAP (see link list at top right) or EGA (see link list at top right).	N.A.
21. Computational models that are central and integral to a study should be shared without restrictions and provided in a machine-readable form. The relevant accession numbers or links should be provided. When possible, standardized format (SBML, CellML) should be used instead of scripts (e.g. MATLAB). Authors are strongly encouraged to follow the MIRIAM guidelines (see link list at top right) and deposit their model in a public database such as Biomodels (see link list at top right) or JWS Online (see link list at top right). If computer source code is provided with the paper, it should be deposited in a public repository or included in supplementary information.	N.A.

G- Dual use research of concern

22. Could your study fall under dual use research restrictions? Please check biosecurity documents (see link list at top right) and list of select agents and toxins (APHIS/CDC) (see link list at top right). According to our biosecurity guidelines, provide a statement only if it could.	N.A.
